# Factors influencing the implementation of interventions for symptoms of posttraumatic stress disorder among hospital-based nurses and physicians during the COVID-19 pandemic: a scoping review

**DOI:** 10.1186/s12913-025-13005-z

**Published:** 2025-07-02

**Authors:** Deliah Katzmarzyk, Daniela Holle, Martina Roes

**Affiliations:** 1 Faculty of Health, School of Nursing Science, Witten/Herdecke University, Alfred-Herrhausen-Straße 50, Witten, 58455 Germany; 2 Hannover Medical School, Institute for Epidemiology, Social Medicine and Health Systems Research, Carl-Neuberg-Straße 1, Hannover, 30625 Germany; 3 Bochum University of Applied Sciences, Department of Nursing-, Midwifery, -and Therapeutic Sciences, Gesundheitscampus 6-8, Bochum, 44801 Germany; 4 German Center of Neurodegenerative Diseases (DZNE), Stockumer Straße 12, Witten, 58453 Germany

**Keywords:** Implementation science, Stress disorders, Post-traumatic, Nurses, Physicians, Hospitals, COVID-19

## Abstract

**Background:**

In the field of posttraumatic stress disorder (PTSD) among nurses and physicians working in an acute hospital setting, various investigations have been conducted on the prevalence of PTSD during the COVID-19 pandemic rather than on the implementation of PTSD-related interventions to improve the mental health of health care workers. It is known that implementation faces challenges, such as social restrictions or the dynamic of the pandemic itself. However, for successful implementation under these conditions, identifying barriers and facilitators is inevitable before using tailored implementation strategies. The following research question was addressed: What are the barriers/facilitators in the implementation of PTSD-related interventions for nurses and physicians working in an acute hospital setting during the COVID-19 pandemic?

**Methods:**

Using a scoping review approach, we conducted systematic literature searches from February to May 2023 in MEDLINE via PubMed and PsychINFO/CINAHL via EBSCO. We included primary studies (protocols), and concept articles focused on influencing factors in the implementation of PTSD-related interventions for nurses and physicians working in an acute hospital setting during the COVID-19 pandemic. We performed data analysis in MaxQDA via evaluative content analysis using the Consolidated Framework of Implementation Research (CFIR).

**Results:**

A total of 19 studies were included. Most of them used an empirical approach to evaluate the intervention during its development or adaptation process. The identified factors were mainly neutral factors that emerged from the inner setting and individuals as the intervention’s target group. The management, the nurses, and the physicians as innovation recipients themselves, and the connection between the inner and outer settings could influence the implementation of PTSD-related interventions.

**Conclusion:**

With these results, decision-makers in organizations in health care systems can be encouraged to implement interventions to improve PTSD among hospital-based nurses and physicians under pandemic conditions. Future research needs to focus on conducting implementation studies to evaluate influencing factors and investigate whether these factors enable or hinder the implementation of PTSD-related interventions.

**Supplementary Information:**

The online version contains supplementary material available at 10.1186/s12913-025-13005-z.

## Introduction

The Coronavirus disease 2019 (COVID-19) was identified for the first time in Wuhan, China [1], and severe acute respiratory syndrome coronavirus type 2 (SARS-CoV-2) has spread throughout the country in a short time worldwide, resulting in an increased prevalence of COVID-19 infections and death [2]. Therefore, the World Health Organization (WHO) declared the outbreak a pandemic [3]. In subsequent months, the number of infected individuals and hospitalized COVID-19 patients has increased globally [2, 4].

In the acute hospital setting, health care workers (HCWs), particularly nurses and physicians [5], are affected by mental health problems with short- and long-term effects, such as symptoms of posttraumatic stress disorder (PTSD) [6, 7].

Lee et al. [8] conducted a systematic review and meta-analysis on the impact of the COVID-19 pandemic on the mental well-being of HCWs in hospitals. Across all analyzed studies, the prevalence of clinical symptoms of PTSD besides depression, insomnia, and anxiety was highest among these professional groups.

Furthermore, Ghaharamani et al. [9] reported in their systematic review and meta-analysis that the aggregate prevalence of insomnia, anxiety, PTSD, depression, and stress among physicians and nurses was higher in comparison to other professional groups of HCWs.

Regarding the prevalence of mental health symptoms during the COVID-19 pandemic at the regional, interregional, or global level, many studies have been conducted [10, 11]. For example, Saragih et al. [5] conducted a systematic review, including a meta-analysis, to map the global prevalence of common mental health problems, like PTSD, anxiety, depression, and distress. The extracted studies included 53,784 participants, 27% of whom were physicians and 43.7% were nurses. The prevalence rate of PTSD was 49%, followed by anxiety (40%), depression (37%) [5].

However, according to the International Statistical Classification of Diseases and Related Health Problems, 10th Revision (ICD-10) – Chapter V, symptoms of PTSD include, for example, intrusive memories, “flashbacks,” nightmares, or “numbness” [12]. In addition, anxiety and depression are commonly associated with PTSD, as well as psychological stress [13]. Risk factors and stress triggers allied with PTSD among HCWs include fear of becoming infected or the family members, high mortality rates, insufficient protection, and high workload [6].

In contrast to the various studies investigating the prevalence and associated factors of mental health issues, only a few studies have reported the evaluation or implementation of interventions for mental health problems among HCWs [14, 15].

It is well known that implementation during the COVID-19 pandemic faces challenges, such as delivering interventions without appropriate approaches that coincide with pandemic conditions, social restrictions, or the dynamics of the pandemic itself [16]. In addition, the management of healthcare organizations came to radical decisions to implement mental health interventions rapidly. Challenges also emerged from the flexible changes in the interventions due to dynamic policy regulations, such as social restrictions [16]. Therefore, considering the individual context in which the implementation is intended, like the acute hospital setting during the COVID-19 pandemic, is crucial to achieve a successful and sustainable use of an intervention [17]. For instance, the compatibility of a PTSD-related intervention within existing hospital structures and working conditions of nurses and physicians could be a facilitator during a non-pandemic context but a barrier in a pandemic context. To face these challenges, further development of existing implementation approaches is required to guide a rapid implementation under pandemic conditions [16, 18].

However, to achieve successful and sustainable implementation of interventions in practice, it is necessary to identify barriers and facilitators that could influence implementation and select tailored implementation strategies [19–22].

Regarding the barriers and facilitators as factors influencing the implementation, the state of the research, described in a systematic review by Pollock et al. [23], revealed insights into the effectiveness of interventions supporting the resilience and mental health of HCWs, as well as barriers and facilitators related to the implementation of these interventions. These results provide recommendations for policy, governments, and decision-makers during the early stages of the COVID-19 pandemic [23]. To analyze barriers to and facilitators of implementation, the authors used the consolidated framework of implementation research (CFIR) by Damschroder et al. [20] to present those factors applicable to different interventions, disease outbreaks, and settings. However, limitations include a lack of synthesis and specific links to interventions, mental disorders, and contexts [23]. This systematic review was also conducted in the earliest stages of the COVID-19 outbreak and included four studies that focused on this context [24–27], published between January and April 2020.

In response to these gaps, our review sought to analyze barriers and facilitators in the implementation of interventions to treat symptoms of PTSD among hospital-based nurses and physicians during the COVID-19 pandemic. The research question guiding our review is as follows:

*What are the barriers/facilitators in the implementation of PTSD-related interventions for nurses and physicians working in an acute hospital setting during the COVID-19 pandemic?*

## Materials and methods

Since investigations on influencing factors in implementing PTSD-related interventions are rare, we conducted a scoping review with less stringent criteria to explore existing evidence on barriers and facilitators. We synthesized beyond a simple descriptive data analysis to produce new evidence on influencing factors [24]. For the elaboration of our research question, the eight steps of the scoping review approach by Peters et al. [25], which are based on the methodology of the Joanna Briggs Institute (JBI), guided our scoping review: [1] developing the research question and objectives; [2] defining the inclusion criteria; [3] planning the systematic literature searches and selection approach; [4] conducting the systematic literature search; [5] performing the evidence screening and selection; [6] conducting the data extraction; [7] performing the data analysis; and [8] presenting the results. For consistency and reporting of this scoping review, the items of the PRISMA Extension for Scoping Reviews (PRISMA-ScR) guided this publication (see Additional File 1) [26].

Two systematic literature searches for this scoping review were conducted.

First, we performed a systematic literature search I to explore interventions for PTSD-symptoms. Based on the knowledge gained from this literature search, we conducted a literature search II to identify further studies investigating barriers and facilitators in implementing the identified interventions. The articles identified in both literature searches (literature corpus) related to the research question were included in this scoping review.

## Defining the inclusion criteria

We employed the PCC-elements (Participants, Concept, and Context) framework to define the inclusion criteria [25]. We determined nurses and physicians as participants who reported symptoms of PTSD based on the documented symptoms associated with PTSD provided by the ICD-10 – Chapter V [12]. Our concept in this publication included barriers against and facilitators for the implementation of PTSD-related interventions in acute somatic hospital settings during the COVID-19 pandemic (2020–2024) (context). All evidence sources that describe or evaluate the process from development to the implementation of PTSD-related interventions were included and were published between 2020 and 2024. For detailed information about the inclusion criteria, see table 1; for the exclusion criteria, see additional file 2 and additional file 3.

**Table 1:** Inclusion criteria defined prior to the scoping review process according to Peters et al. [25]

Criteria	Definition
Participants	• Nurses and physicians showing one of the following symptoms of posttraumatic stress disorder (PTSD) as recipients of the interventions: *Distress*,* Intrusive memories*,* Flashbacks*,* Disturbing dreams*,* Nightmares*,* Emotional blunting*,* Coldness*,* Social distancing*,* Disturbing memories*,* Reminiscence*,* Anhedonia*,* Avoidance of activity*,* Insomnia*,* Anxiety*,* Depression*,* Suicidal ideations*,* Acute stress disorder* [12, 28]
Concept of interest	• Barriers against and facilitators for the implementation of interventions for the treatment of PTSD symptoms
Context	• Acute somatic hospitals during the COVID-19 pandemic
Types of evidence sources	• Empirical/concept publication that present the interventions description and delivery and/or the evaluation of the intervention development or implementation or daily practice (e.g., evaluation studies, implementation studies, study protocol, feasibility studies)
Other	• Languages: German and English • Publication years: 2020–2024

We excluded articles if they dealt with non-PTSD-related interventions and directed nurses and physicians as target groups working in different contexts, such as psychiatric or ambulant settings. Moreover, we examined the references list of all review articles using backward citation tracking to identify potential articles. Subsequently, the reviews were excluded from the literature corpus and not used in the analysis. Additionally, articles published before 2020 that did not address the COVID-19 pandemic were removed.

### Planning and conducting the systematic literature search

To identify barriers and facilitators in implementing PTSD-related interventions for nurses and physicians working in an acute hospital setting during the COVID-19 pandemic, two literature searches were performed in MEDLINE via PubMed and PsychINFO/CINAHL via EBSCO between February and May 2023, with a research update in May and July 2024.

Before we developed the search string for the first database, the researcher (DK) conducted an initial hand search on MEDLINE, PsychINFO, CINAHL, and Google Scholar to identify synonyms for the PCC elements. For consistency, the identified terms were discussed with one researcher (MR) to specify the search string and to narrow down potential records. First, one search string was developed for MEDLINE by one researcher (DK) with a nursing background and verified by two other researchers (DH, MR) used the peer review of electronic search strategies (PRESS) [27].

Second, DK modified the search string for PsychINFO based on the database’s specifications. Nordhausen and Hirt’s Ref Hunter in web format was a general guide [29] for transparent and comprehensive process communication.

The search strings were stored online, with a daily reminder for new articles. Furthermore, we conducted supplementary searches following the recommendation of Cooper et al. [30]. We also performed backward citation tracking via a reference list to search for potential publications and forward citation tracking via Google Scholar. Additionally, a search in trial registers and a hand search via Google Scholar was performed. The data collection process of both systematic literature searches is available as a research protocol in additional file 2 for interventions and additional file 3 for influencing factors.

### Performing the evidence screening and selection

For the study selection, the records were transferred to EndNote 20.5 by DK to search for duplicates and uploaded to the online tool Rayyan [31]. Then, title-abstract and full-text screening took place in Rayyan by DK in two iterations to minimize potential bias. Additionally, four studies were examined independently by two researchers (DH, MR) according to the predefined inclusion criteria. At the end of the process, discrepancies were discussed in exchange with the three researchers (DK, DH, MR).

### Conducting data extraction

One researcher (DK) used MaxQDA 2022 to extract general information about the characteristics of the included studies: publication, year, intervention, and type of evidence. Knowledge about the kind of evidence is needed to interpret the results of the analyzed influencing factors correctly. Since most of the included studies were effectiveness studies, only one investigated factor affecting implementation. However, the type of evidence is crucial regarding the interpretation and usefulness of results.

### Performing data analysis

Since the included studies did not explicitly present barriers and facilitators, simple data extraction could not be performed. Therefore, we chose an interpretative approach using evaluative qualitative content analysis [32], which took place in MaxQDA 2022 by one researcher (DK). We employed the CFIR to investigate barriers and facilitators [20]. This framework contains an accumulation of five domains that could facilitate the process of theory development as well as the verification of which approach operates for the implementation of intervention in a defined context and for which reasons [20].

We decided to focus our investigation on three domains of the CFIR—(a) the outer setting, (b) the individuals, and (c) the inner setting—because of the deliberations of Blake et al. [33]. They noted that mental health interventions for HCWs should address organizational and individual characteristics, and past pandemics have demonstrated the significant impact of settings and organizations on the psychological outcomes of workers [33]. We also know that the pandemic has caused legal and social changes [16]; therefore, the external environment may also have influenced the implementation of interventions.

To define our main categories and dedicate the analyzed influencing factor to the appropriate domain, we used the definitions of the three domains and their subdomains of the CFIR [20]. Each influencing factor was set as an evaluative category so that we could analyze whether it was a barrier, facilitator, or neutral factor in the implementation. Thus, table 2 presents our definitions from the evaluative categories.

**Table 2:** Category definitions of the evaluative categories in the evaluative content analysis of the influencing factors

Evaluative category	Definition applied in this review
Barrier	A barrier or hindering factor is defined when data indicate that for example: ▪ Managers, like department heads mentioned, that an intervention is not necessarily ▪ Innovation recipients, like nurses and physicians voice misgivings about using a digital intervention without the appropriate technical skills ▪ Nurses reported time limitations using the intervention, based on lack motivation through the high workload during shifts
Facilitator	A facilitator or promoting factor is defined when data indicate for example: ▪ The compatibility between the PTSD-related interventions and the implemented context, based on how the intervention is conceptualized. For instance, with a digitally created intervention, nurses and physicians could use the intervention flexible ▪ The partnership and connection between the inner setting, like the hospital and the outer setting, for example a collaborative university. Through this partnership, the development and adaptation process could be scientifically monitored ▪ The innovation recipients, like nurses and physicians are aware of their capabilities using the learned skills in terms of the intervention in their daily work-life
Neutral factors	A neutral factor or a factor with an unclear impact is defined as when data did not indicate whether a factor hindered or promote the implementation. Thus, from the data we could not clearly analyze a barrier or facilitator.

For a transparent reporting of our analytical process, we present an example of coding for each domain of the CFIR in table 3. The data analysis was performed in two iterations by DK. First, the influencing factors were analyzed using the deductive category system we created before, using the domains and subdomains of the CFIR. Second, the influencing factors were evaluated, and an evaluative content analysis was applied (see Table 2). After the first step, the results were discussed among the three researchers (DK, DH, MR) to clarify and remove ambiguity.

**Table 3:** Example of codings for each identifed domain of the CFIR and the analyzed influencing factors

Domains of the CFIR	Example of Codings of the influencing factor	Example of analyzed barrier, facilitator or neutral factor
*Inner Setting*		
Work infrastructure	*“Participants described work as chaotic and stressful […]”* [34]	Neutral factor
Compatibility	*“We shortened sessions from 90 to 60 min to fit better within a work setting and tailored case examples to HCWs.”* [35]	Neutral factor
*Outer Setting *		
Critical Incidents	*“Due to physical distancing rules*,* the possibility of home confinement during the pandemic”* [36]	Neutral factor
Partnership & Connections	*“A Steering Committee of key faculty with expertise in these areas was formed with representatives from Departments of Psychiatry & Behavioral Sciences*,* Anesthesiology*,* and Risk Management*,* including a faculty member currently serving as a Colonel in the Army Medical Reserve.”* [37]	Neutral factor
*Individuals Roles*		
Innovation recipients	*“‘I thought it was really good*,* I mean*,* like*,* for us who don’t get on so well with counselling it’s a really good thing.”* [34]	Facilitator
*Characteristics *		
Capability	*“Sessions were delivered remotely by HCW peers […]”* [36]	Neutral factor
Opportunity	*“The greatest barrier to use of the app […] was time constraints.”* [38]	Barrier

The results are presented in a table with color coding of the source of evidence (e.g., RCT or conceptual publication) for a straightforward interpretation. They can be seen in additional file 4. In the results sections, we report the most frequently coded external and internal environmental and individual factors and whether they were identified as facilitating, hindering, or neutral. Additionally, we created a diagram to visualize the percentage distribution of identified influencing factors per analyzed domain of the CFIR [20], which can be seen in Fig. 2.

## Presenting results

**Fig. 1:**
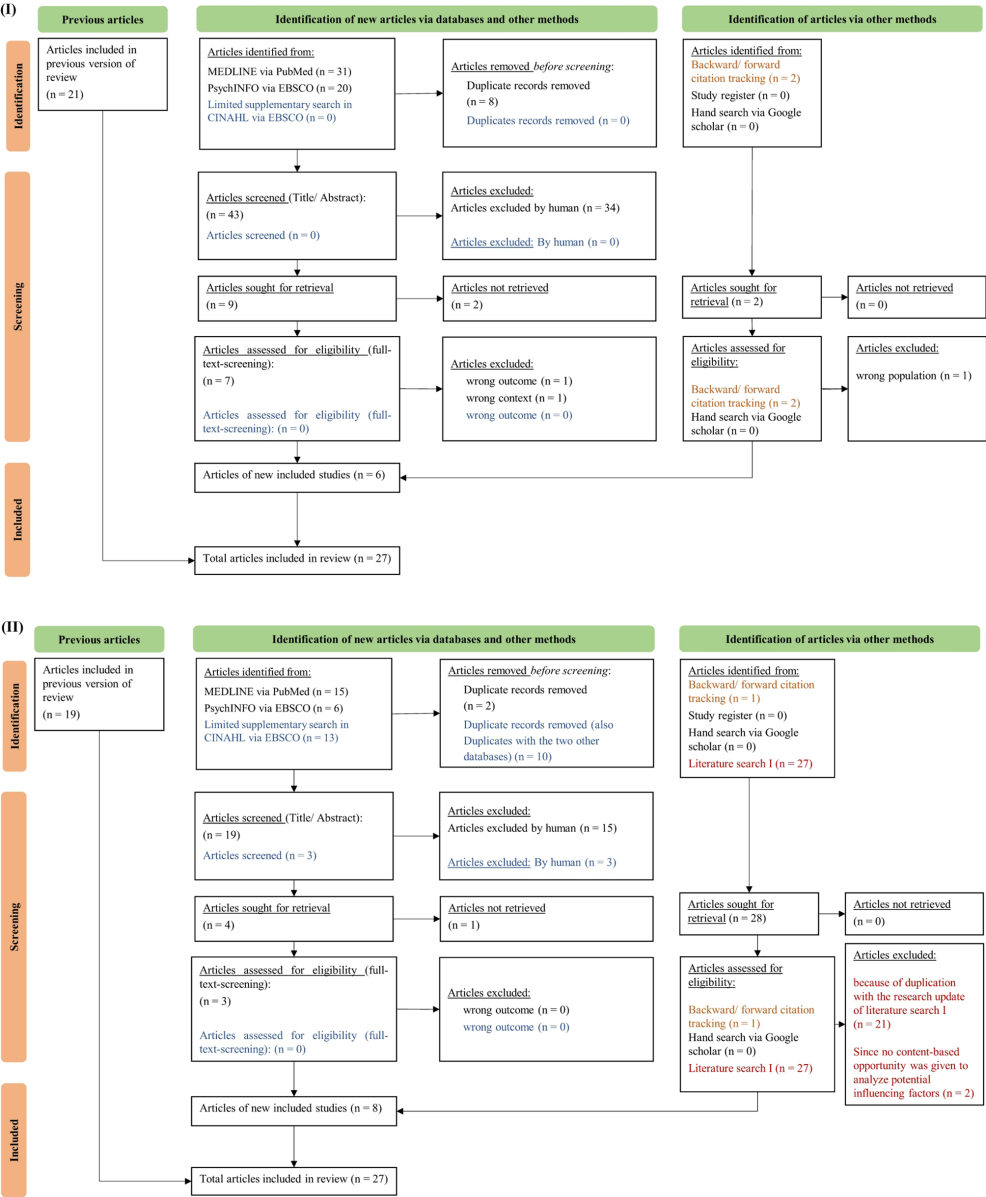
PRISMA flowchart of both literature searches: (I) literature search I and (II) literature search II according to (Page et al. [39])

Since both systematic literature searches used one literature corpus, we present each flow chart, including the research update, in Fig. 1.

As shown in the flow chart (I), 21 articles resulted from the systematic literature search in the previous version of the review. With the research update, we identified another eight articles, which resulted in 27 articles for inclusion in the review. In flow chart (II), we present the process of literature search II in MEDLINE, PsychINFO, and CINAHL. This resulted in 19 articles from the previous version of the review. Notably, we screened the 27 articles from the research update of the literature search I for eligibility since, in some articles, no content-based opportunity was given to analyze potential influencing factors (*n* = 2), and we identified duplicates with the research update of the literature search I (*n* = 21). Finally, we included 27 articles for our data analysis (see Fig. 1).

### Study characteristics

Most articles were published in 2021 (*n* = 7), followed by six in 2023. Twelve publications were identified in 2020 and 2022; two were published in 2024.

Most of the extracted articles (*n* = 20) reported the results of conducting a study via an empirical approach, such as an RCT or feasibility study. Six publications also present a study protocol for conducting a trial. Five of these six study protocols have published their results and are listed below (see Table 4). Finally, one publication was a concept study.

**Table 4:** Characteristics of the included studies (*n* = 27) according to the necessity for the research question

Publication	Year	Intervention (digital/in-person)	Study participants	Context
**Type of article: Study protocol**				
Wang, L. et al.	2020	‚Preparing ME’ based on Psychological First Aid (PFA) and the RAPID-Model (in-person)	▪ Frontline HCWs in all professions (e.g., nurses, physicians, pharmacists) ▪ > 18 years old ▪ Employed in departments that would be called to emergency response (e.g., emergency departments, intensive care unit) ▪ Non-mental health frontline HCWs	▪ Second Xiangya Hosital of Central South University as part of the national and regional emergeny rescue service in China
Weiner, L. et al.	2020	‘My Health Too’ based on Cognitive behavioral Therapy (CBT), Psychoeducation by Lazarus and Folkman´s transactional stress model (digital)	▪ Medical doctor, nurses, physiotherapists, psychologists, orderlies, hospital porters, ambulance drivers, nursing and medical students working in the hospital ▪ 18–70 years old ▪ Fluent in French language	▪ Six hospitals of the East region of France, i.e., Hôpitaux Universitaires de Strasbourg, Hôpitaux Civils de Colmar, Groupe Hospitalier Régional de Mulhouse Sud-Alsace, Centre Hospitalier Universi-taire de Nancy, Centre Hospitalier Universitaire de Besançon, and Centre Hospitalier Universitaire de Dijon; the East region was the region that was the most affected by the COVID-19 in France
Jovarauskaite, L. et al.	2021	Cognitive‒behavioral Therapy and mindfulness-based internet-delivered stress recovery intervention (FOREST) (digital)	▪ Licensed nurses working in the healthcare system throughout the country ▪ ≥ 18 years old ▪ Comprehend Lithuanian ▪ Possesses a computer, tablet, smartphone, or similar device with Internet access	▪ Healthcare institutions in Lithuania (e.g., hospitals, primary care centers)
Morina, N. et al.	2021	RECHARGE based on Psychoedcuation (digital)	▪ HCWs (e.g., nurses, physicians) ▪ ≥ 18 years old ▪ German-speaking ▪ Access to a teleconferencing platform ▪ Moderate level of distress as defined by a score of ≥ 16 on the Kessler Psychological Distress Scale	▪ Hospitals in Switzerland during the COVID-19 pandemic
Dong, L. et al.	2022	Stress First Aid (SFA) based on Stress continuum and Psychological First Aid (PFA) (digital)	*From* *ambulatory* *centers* ▪ HCWs ▪ Supporting staff, who are patient-facing (e.g., front desk staff) *From* *hospitals* ▪ Only HCWs from different teams or units	▪ Ambulatory centers ▪ Hospitals in multiple teams or units
Singh, L. et al.	2022	Remotely delivered guided brief intervention (digital)	▪ ≥ 18 years old ▪ Hospital and care facilities (e.g., ICU, ambulance, intermediate care, ward) ▪ Experienced at least one traumatic event in relation to their clinical work during the pandemic ▪ Traumatic event must satisfy the DSM-5-PTSD Criterion A definition of trauma (i.e., exposure to actual or threatened death, serious injury, or sexual violence by “Directly experiencing the traumatic event(s)” or “Witnessing, in person, the event(S) as it occurred to others”) and must have occurred since the star of the COVID-19 pandemic ▪ Report of distressing intrusive memories in the previous week ▪ Participants must also be able and willing to briefly write down these memories ▪ Participants must be alert and orientated ▪ Having access to an internet enabled smartphone and sufficient mobility use ▪ Be fluent in spoken and written Swedish	▪ Hospital and care facilities (e.g., ICU, ambulance, intermediate care, ward)
**Type of article: Empirical publication**				
Blake, H. et al.	2020	Digital learning package (digital)	*Three Stakeholder groups* 1. Healthcare students 2. Registered nurses 3. HCWs from nursing and the allied health professions	▪ Different healthcare settings
Mellins, C. et al.	2020	CopeColumbia’ based on Cognitive‒behavioral therapy (CBT), Acceptance and Commitment Therapy (ACT) (digital)	▪ HCWs (e.g., physicians, leaders, non-clinical staff) of the Columbia University Irving Medical Center (CUIMC)	▪ CUIMC
Sulaiman, A. et al.	2020	Remote Psychological First Aid (PFA) (digital)	▪ HCWs (e.g., physicians, medical officers, nurses, disinfectant teams, cleaners, and others)	▪ University Malaya Medical Centre
Bureau, R. et al.	2021	My Health Too’ based on Cognitive behavioral Therapy (CBT), Psychoeducation by Lazarus and Folkman´s transactional stress model (digital)	*Profession* ▪ Registered nurses (*n* = 3) ▪ Practicing students (*n* = 2) ▪ Special need educator (*n* = 2) ▪ Paramedic (*n* = 1) ▪ Social worker (*n* = 1) ▪ Administration position (*n* = 1)	▪ Hôpitaux Universitaires de Strasbourg (*n* = 8) ▪ Centre Hospitalier de Rouffach (*n* = 2)
Hannig, C. et al.	2021	Hamburger concept with peer approach (in-person)	▪ Nurses (*n* = 26)	▪ University Hospital
Kanellopoulos, D. et al.	2021	Psychological First Aid intervention (PFA) (CopeNYP) (digital)	▪ Registered and Nursing practitioners ▪ Patient Support Staff (e.g., Mental Health Workers, Unit clerks, Medical Assistants, Speech and Occupational therapists) ▪ Administrative Support Staff (Administrators, finance, research support, development, information technology, Human Resources) ▪ Physicians/Doctoral level Faculty and Trainees	▪ Hospital
Lefevre, H. et al.	2021	The Port Royal Bubble’ (La Bulle de Port Royal) (in-person)	*In total 800 staff visits were counted:* ▪ Nurses (57%) ▪ Physicians (11%) ▪ Technical (11%) and administrative staff (11%) ▪ Nurses´aides (10%)	Cochin Hospital (APHP, Paris) ▪ Staff from principal departments admitting patients with COVID-19 ▪ Staff from the medical department (25%) ▪ Staff from the emergency department (25%) ▪ Staff from the ICU (17%)
Trottier, K. et al.	2021	Recovering from Extreme Stressors Trough Online Resources and E-health (RESTORE) based on Cognitive Processing Therapy (CPT) (digital)	▪ Exposure to a COVID-19-related traumatic or extreme stressor ▪ A score above clinical threshold on either the Patient Health Questionnaire-9, Generalized Anxiety Disorder Scale-7, or PTSD Checklist Scale-5 ▪ Fluent in English ▪ Access to high-speed internet and a computer or tablet	▪ Not specified
Sagaltici, E. et al.	2022	Online format of the Recent Event and Eye Movement Desensitization (EMDR) (digital)	▪ Physicians (*n* = 2) ▪ Nurses (*n* = 2) ▪ Other HCWs/medical staff (*n* = 4) ▪ Other HCWs/non-medical staff (*n* = 6) ▪ Family members who have been diagnosed with COVID-19 (*n* = 5) ▪ Being quarantined (*n* = 7) ▪ Diagnosed with COVID-19 (*n* = 5)	▪ Bağcılar Training And Research Hospital
Solomonov, N. et al.	2022	CopeNYP based on Psychological First Aid (PFA) (digital)	*A total of 534 HCWs participated in the program*: ▪ Nursing staff (*n* = 188) ▪ Patient support staff (*n* = 130) ▪ Administrative support staff (*n* = 122) ▪ Physicians/doctoral level faculty trainees (*n* = 74) ▪ Maintenance workers (*n* = 13) ▪ Employees´ family members (*n* = 7)	▪ In Hospitals
Trottier, K. et al.	2022	Recovering from Extreme Stressors Trough Online Resources and E-health (RESTORE) based on Cognitive Processing Therapy (CPT) (digital)	*In total 21 participated in the study:* ▪ Nursing (*n* = 11) ▪ Administrative (*n* = 4) ▪ Personal support (*n* = 3) ▪ Respiratory therapist (*n* = 2) ▪ Security (*n* = 1)	▪ Hospital (*n* = 16) ▪ Long-term care (*n* = 2) ▪ Declined to provide (*n* = 3)
Fogliato, E. et al.	2022	Eye Movement Desensitization and Preprocessing Therapy (EMDR) (in-person)	▪ Doctors ▪ Nurses ▪ Nursing Assistant ▪ Other HCWs from places other than COVID-wards	▪ Hospital ▪ Critical area ▪ COVID Department ▪ Other
Dumarkaite, A. et al.	2023	Cognitive‒behavioral Therapy and mindfulness-based internet-delivered stress recovery intervention (FOREST) (digital)	*Intervention group *(*n* = 77) *Control group *(*n* = 91) ▪ Nurse (I**I**: *n* = 72 /: *n* = 88) ▪ Assistant nurse (I: *n* = 5 / C: *n* = 3) *Work experience:* ▪ < 2 years (I: *n* = 10 / C: *n* = 6) ▪ 2–5 years (I: *n* = 12 / C: *n* = 12) ▪ 6–10 years (I: *n* = 12 / C: *n* = 7) ▪ > 10 years (I: *n* = 43 / C: *n* = 66)	Hospital, Department ▪ Surgical ▪ Therapy ▪ Anesthesiology and intensive care ▪ Outpatient care ▪ Emergency ▪ Other
Morina, N. et al.	2023	RECHARGE based on Psychoedcuation (digital)	*Intervention group [I]* (*n* = 82) *Control group *[C] (*n* = 78) ▪ Physicians (I: *n* = 29 / C: *n* = 37) ▪ Nurses (I: *n* = 35/C: *n* = 26) ▪ Allied health (I: *n* = 18 / C: *n* = 15) ▪ Professional experience from 0 years to 49 years (I: *n* = 12.59/C: *n* = 15.44)	Not reported
Pratt, E.H. et al.	2023	LIFT mindfulness app (digital)	*Intervention group [I]* (*n* = 69) *Control group [C]* (*n* = 33) *Work location* ▪ Emergency department (I: *n* = 15 / C: *n* = 10) ▪ Surgical ICU (I: *n* = 8 / C: *n* = 5) ▪ Medical ICU (I: *n* = 39 / *n* = 17) ▪ Medicine stepdown (I: *n* = 6 / C: *n* = 1) ▪ Duke Raleigh Hospital (I: *n* = 1 / C: *n* = 0) *Years as a nurse* ▪ < 1 year (I: *n* = 16 / C: *n* = 8) ▪ 1–5 years (I: *n* = 33 / C: *n* = 17) ▪ 6–10 years (I: *n* = 9 / C: *n* = 3) ▪ > 10 years (I: *n* = 11 / C: *n* = 5)	▪ COVID-19 units at Duke University Hospital, including • a medical/surgical stepdown unit, two ICUs, and the emergency department
Mediavilla, R. et al.	2023	Stepped-care programme based on a combination from stress management course Self Help Plus (SH+) and a brief intervention based on cognitive-behavioral a problem-solving strategies called Problem Management Plus (PM+) (digital)	*Intervention group [I]* (*n* = 115) *Control group [C]* (*n* = 117) *Type of job:* ▪ Physician (I: *n* = 22 / C: *n* = 28) ▪ Nurse (I: *n* = 64 / C: *n* = 66) ▪ Nurse technician (I: *n* = 17 / C: *n* = 12) ▪ Administration (I: *n* = 5/C: *n* = 1) ▪ Other (I: *n* = 7/C: *n* = 9) *Job facility:* ▪ Hospital (I: *n* = 75 / C: *n* = 72) ▪ Primary care facilities (I: *n* = 33/C: *n* = 35) ▪ Specialized care facilities (I: *n* = 2 / C: *n* = 3) ▪ Emergencies (I: *n* = 4 / C: *n* = 6) ▪ Other (I: *n* = 1/C: *n* = 1)	▪ Madrilenian or the Catalan Department of Health (doctors, psychologists, nurses, nursing technicians, orderlies and administrative staff)
Kirykowski, K. et al.	2023	COVID Coach a self-management app (digital)	*Active group [A]* (*n* = 16) *Waitlist group [W] *(*n* = 18) ▪ Profession not reported ▪ Years of working (A: *n* = 9.9/W: *n* = 6.5)	▪ Government healthcare facilities in the Western Cape of South Africa during the COVID-19 pandemic
Iyadurai, L. et al.	2023	Brief, mechanistically informed behavioral intervention (digital)	▪ clinical role in a National Health Service (NHS) ICU or equivalent during the COVID-19 pandemic Intervention group [I] (total of *n* = 43) Control group [C] (total of *n* = 43) Time as HCW (years): ▪ I: *n* = 16.4 ▪ C: *n* = 13	▪ Not specified
Meredith, L.S. et al.	2024	Stress First Aid (SFA) based on Stress continuum and Psychological First Aid (PFA) (digital)	FQHCs *Intervention group [I]* (*n* = 245) *Control group [C]* (*n* = 183) *Professional role:* ▪ Clinician (I: *n* = 31 / C: *n* = 59) ▪ Nurse (I: *n* = 18/ C: *n* = 8) ▪ Assistant or technician (I: *n* = 111/ C: *n* = 43) ▪ Administrative or other (I: *n* = 85 / C: *n* = 73) ▪ ≤ 5 years employed at the site (I: *n* = 178 / C: *n* = 130) ▪ ≤ 5 years in the profession (I: *n* = 118 / C: *n* = 67) *Hospitals* Intervention group [I] (*n* = 617) Control group [C] (*n* = 1032) *Professional role:* ▪ Clinician (I: *n* = 46 / C: *n* = 160) ▪ Nurse (I: *n* = 287/C: *n* = 532) ▪ Assistant or technician (I: *n* = 189/C: *n* = 242) ▪ Administrative or other (I: *n* = 95 / C: *n* = 98) ▪ ≤ 5 years employed at the site (I: *n* = 357/C: *n* = 566) ▪ ≤ 5 years in the profession (I: *n* = 230 / C: *n* = 352)	▪ Hospitals and federally qualified health centers (FQHCs) during the pandemic
Pihlgren, S.A. et al.	2024	Simple cognitive task intervention	▪ Seven participants ▪ Varying types of workplace (e.g., intensive care unit, elderly care, and pediatrics) ▪ Participants had worked for 3–26 years in healthcare	▪ Hospital and care facilities (e.g., ICU, ambulance, intermediate care, ward)
**Type of article: Concept publication**				
Albott, C. et al.	2020	Battle Buddies’– Psychological Resilience intervention based on Anticipate-Plan-Deter (APD) (in-person)	▪ HCWs	▪ Not specified

### Influencing factors of the implementation of PTSD-related interventions

**Fig. 2:**
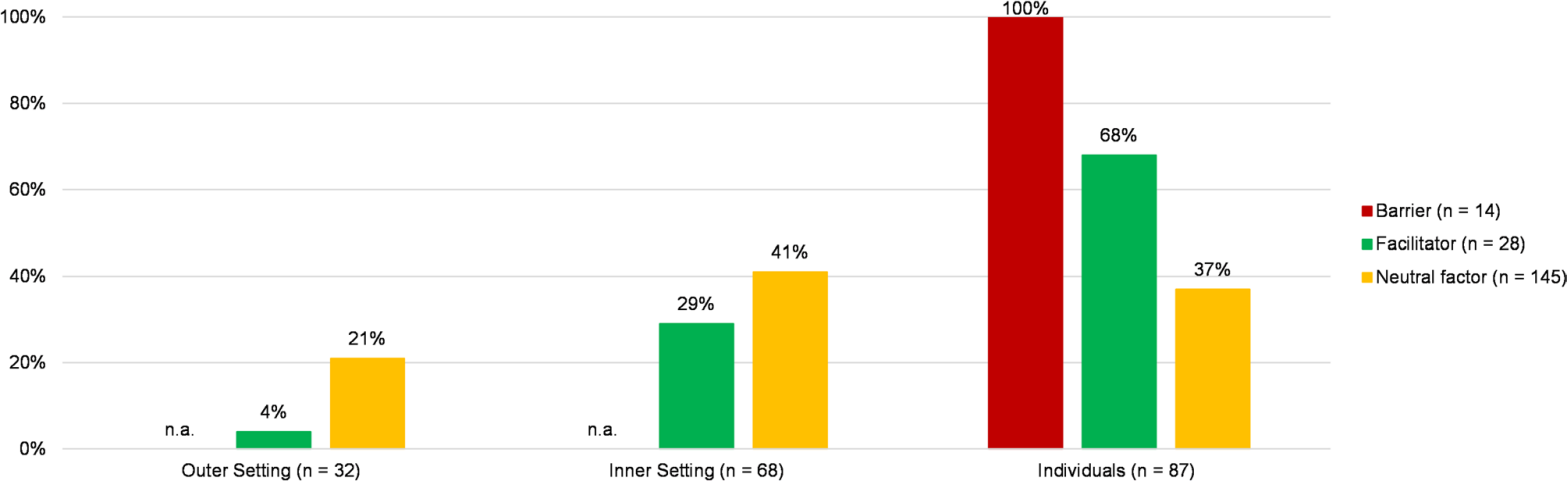
Quantitative distribution of the total coded number of barriers (*n* = 14), facilitators (*n* = 28), and neutral factors (*n* = 145) within the domains of the CFIR [20]. We determined the percentage distribution of the total coded number of factors. If factors were not coded, the description was labeled with ‘n.a.’ (*n* = 0) (own visualization)

The results indicate that the outer setting, such as policy, governance, or even the COVID-19 pandemic as a critical incidence, was analyzed the least frequently (*n* = 32) as the other domains. The inner settings, e.g., the hospitals themselves and their staff, such as nurses and physicians, as the target groups of PTSD-related interventions, were analyzed most frequently (*n* = 155) (see Fig. 2).

According to the total number of barriers analyzed (*n* = 14), all were identified as related to the individuals (100%); thus, none were from the inner or outer setting. Most analyzed facilitators originate from the inner setting (29%) and individuals (68%). Additionally, the neutral factors with an unclear impact mainly were identified from the inner setting (41%) and the individuals (37%). The smallest number of neutral factors (21%) and facilitators (4%) originate from the outer setting.

In the subsequent section, we have presented the most frequently analyzed influencing factors from our scoping review across the three domains of the CFIR (outer setting, inner setting, and individuals).

#### Outer setting

##### Facilitator

A partnership and connection between the inner setting and outer setting could facilitate the implementation of PTSD-related interventions [40]. Blake et al. [40] report that the users liked to disseminate the intervention with an external professional network to call attention to the developed digital intervention because they were confident in its usefulness:

*“They reported sharing the information in the following ways: circulating the package link around their clinical teams*,* colleagues*,* and students; sharing the resource with external professional networks via email*,* print media*,* websites*,* and social media […]”* [40].

##### Neutral factors

The COVID-19 pandemic itself, as a condition, could influence the implementation of PTSD-related interventions. Six empirical studies report the difficulty of applying those interventions based on the abovementioned pandemic-related conditions [36, 41–44]. Additionally, using interventions privately at home seems impossible, even if the intervention is digitally created [41–44]. Besides, this potential factor is mentioned within four study protocols [45–48]:

*“Importantly*,* such interventions have to address extraordinary circumstances due to the crisis*,* which*,* in the case of the COVID-19 pandemic*,* include home confinement*,* social distancing*,* and workloads potentially much higher than usual.”* [45].

The external stigma of HCWs seeking help for mental well-being is another neutral factor with unclear impact. Five empirical studies report that making use of help based on mental health problems often correlates with stigma resulting from the local attitudes of society. Healthcare professionals are particularly affected by this stigma, as they have an attitude of putting patients first and should not show any mental health issues [34, 43, 44, 49]. Trottier et al. [44] aim to reduce the likelihood of stigma with their developed intervention. Additionally, this potential factor is mentioned in the study protocol for conducting a trial by Wang et al. [50].

#### Inner setting

##### Facilitators

The compatibility between PTSD-related interventions and the implemented context could facilitate the implementation. One empirical study reports that their developed digital intervention is compatible with nurses’ and physicians’ highly flexible and busy schedules [41]. In addition, if an intervention could be applied in different settings, like at home or during a break at work, nurses and physicians are more likely to adopt it [40, 41]. In their study protocol, Singh et al. [48] mention that their intervention is easily adaptable to everyday life.

Tension for change has been identified as another facilitator. In their empirical study, Hannig et al. [51] report that awareness of the current situation and associated problems might promote the implementation. Therefore, the tension of feeling better in HCWs could facilitate the implementation of PTSD-related interventions.

*“The impetus for its development came from a survey of staff in the Emergency Department at the University Medical Centre*,* Hamburg-Eppendorf*,* which revealed a great need to deal with the consequences of violence and other stressful experiences.”* [51].

The culture of deliverer-centeredness in a hospital could also increase the likelihood of using PTSD-related interventions. One empirical study reports that providing support from the institution demonstrates estimation [40]. Another study reveals that nurses and physicians are more likely to adopt a PTSD-related intervention if the opportunity is provided at work [51]. Blake et al. [40] conclude the following in their empirical study:

*“Both healthcare students and registered healthcare professionals mentioned that providing materials to support psychological wellbeing*, *alongside other support mechanisms*, *would demonstrate that their employer (or university) valued them as individuals.”* [40].

##### Neutral factors

Relational connections within the inner settings in terms of formal/informal relationships, teamwork [52], or interprofessional collaboration [37, 53] for disseminating and implementing PTSD-related interventions could influence implementation. Moreover, the relational connections formed through networking with external institutions, like research institutes or universities, could be a potential influencing factor [37, 43]. Kanellopoulos et al. [42] report in their empirical study that study participants would recommend the intervention to their fellows:

*“[…] word-of-mouth referrals grew as employees with positive experiences began to recommend the service to their coworkers.”* [42].

Structural characteristics such as ‘physical,’ ‘information technology,’ and ‘work’ could also influence the implementation of PTSD-related interventions in a hospital. One study protocol mentions a general characteristic, such as the type of hospital as a designated emergency response, that caused medical rescue teams to act as soon as possible under pandemic conditions [50]. Kanellopoulos et al. [42] highlight the strong administrative infrastructure in their empirical study. Regarding the implementation of face-to-face interventions, two empirical studies describe the flexibility of the implemented institutions in relocating space and creating new opportunities for providing the intervention [52, 53]. Regarding digital interventions, two empirical studies refer to the relevance of having preconditions of information technology infrastructure, data storage [53], and an adapted technology system for telecommunication [42]. Another study protocol mentions difficulty accessing hardware such as a desktop computer [48]. Additionally, the study protocol of Wang et al. [50] mentions the existing work infrastructure as a potential influencing factor, which includes, for example, regular critical incident response training or a centralized emergency response system for employees.

Access to knowledge and information, such as having the opportunity to conduct ongoing training or improving knowledge and skills from the trainers themselves, is mentioned in two study protocols [45, 50]. Four empirical publications report the use of concurrent trainers for intervention providers, with recipients such as nurses and physicians receiving access to guidance and training and guidance to promote engagement to increase adherence [42, 51, 53].

#### Individuals

##### Barriers

High-level leaders, such as management with department heads who believe that PTSD-related intervention is not needed, were identified as barriers mentioned in a concept publication [37]:

*“[…] Although some providers may feel they do not need this program […]”* [37].

Innovation recipients, such as nurses and physicians with concerns about not having the technical skills [38, 40] or, in general, the opportunity to use a digital intervention, could hinder the adoption and implementation of PTSD-related interventions, as reported in four empirical studies [34, 38, 40, 41]. Three empirical studies show that HCWs are concerned about not having enough time to use the intervention completely [38, 41, 44]. Additionally, Ahmed Pihlgren et al. [34] report in their empirical study that nurses and physicians cannot use the intervention when needed [34]. Furthermore, the lack of motivation to use PTSD-related interventions resulted in an estimate of a hindering factor and is pointed out by one empirical study [41]:

*“[…] because ‘telling myself’ ‘I am going to take 25 min now to watch or listen to this’ seems impossible’.”* [41].

In addition, one empirical study reports that nurses and physicians found the intervention inappropriate when it required support [34]. In another empirical study by Kirykowisk et al. [38], innovation recipients mentioned that they could not generate impacted engagement with the intervention.

##### Facilitators

Innovation recipients, such as nurses and physicians, could also facilitate the implementation of PTSD-related interventions with high confidence in their capabilities, such as using the learned skills in terms of the intervention in their daily work-life [41, 52]. Additionally, three empirical studies report that innovation recipients have time and confidence to act as facilitators to promote the adoption of the intervention [34, 40, 41]. Another empirical study refers to the following:

*“She had tried a breathing and mindfulness exercise in the evening and found it very soothing. The following morning*,* she was called after numerous emergencies at work*,* and she was stressed by what awaited her. During the commute*,* she remembered the exercise*,* did it*,* and described arriving at work ‘calmer and feeling more capable of facing the day.’”* [41].

Furthermore, one empirical study [52] indicates that nurses and physicians know what they need, when, and how.

Innovation deliverers, who can also be nurses, physicians, or other HCWs [20], have the ability and opportunity to provide interventions [40, 42]. One empirical study reports that the motivation to drive the implementation of the intervention, however, improved when the intervention aligned with the personal values of the innovation deliverers, resulting in a meaningful experience [42]. Additionally, two empirical studies refer to the opportunity to deliver innovation within the inner setting because nurses and physicians are familiar with their workplace characteristics [42]. Besides, the deliverers must first be introduced into the intervention to gain an understanding [34]. Finally, one concept study [37] and three empirical studies [34, 40, 41] indicate that if the deliverers had a positive attitude toward the digital intervention, they could increase the enthusiasm of other colleagues to use and recommend the intervention.

##### Neutral factors

All high-level leaders in general, such as department heads [37], site leaders, chief medical/nursing officers [54], or hospital leaders [42], could influence the implementation of PTSD-related interventions and were identified in one concept study [37], one study protocols [54] and one empirical study [42].

Implementation facilitators, such as mental health consultants [35, 37], site champions [54], or trained clinicians [42] with expertise in this area, could also influence implementation. Two empirical studies [42, 43] and one study protocol [54] report skills and capacities based on experience.

Implementation leads, such as peers [51] and ‘champions’ [54], could lead to local implementation as a human factor in the acute healthcare setting. One study protocol [54] mentions that to fulfill the role of an implementation leader, knowledge or competence is necessary:

*“Once trained*,* the champions implement their training plan at their organizations for their HCW peers.”* [54].

Peers might also fulfill their roles since they have experience with the intervention and trust in its effectiveness and usefulness. Therefore, they can recommend the intervention to other team members [36, 41, 42, 55].

## Discussion

Between 2020 and 2023, little or no implementation research on interventions treating symptoms of PTSD in the target population was conducted, even if planned (study protocol). No other implementation studies or general studies focused specifically on investigating barriers and facilitators could be included in our scoping review. However, the most identified factors did not show a clear positive/negative impact on the implementation process. This significant knowledge gap needs to be addressed in future research, especially with the aim of sustainable implementation during the COVID-19 pandemic to prevent HCWs’ mental well-being beyond critical incidents.

Contrary to the assumption that the outer setting, including policy, government, or institutions such as the WHO, would be essential and, therefore, a significant factor influencing the implementation of interventions or even an incentive during the pandemic, had a minimal impact. Many studies described the intervention’s rationale as coming from the motivation or the tension for change in the inner setting [20]. The inner setting, such as the hospitals, included an awareness of the burden for HCWs, the associated mental health issues, and the need for support. Pollock et al. [23] reported a contrary finding. The organizations that employed the HCWs were unaware of the problems, needs, or tension for change. This lack was considered a barrier to implementation [23]. A possible explanation is the time the review was conducted, which was in the earliest phases of the pandemic [23]. The awareness of the urgency and importance of the issue has potentially increased recently [6, 56–58]. Therefore, the organization initiated these interventions’ development, adaptation, and implementation more effectively [51]. According to the reported rationale for conducting or planning studies, in addition to the COVID-19 pandemic, there was a need for support in mental health issues for the target group [37, 51]. Therefore, our results indicate that the internal setting seems to have a significant influence on the implementation of interventions for symptoms of PTSD worldwide, independent of regional pandemic occurrence. Therefore, organizations should use this kind of influence from their employees to focus on the sustainability of these interventions, not only to prepare for future disease outbreaks but also to prevent mental well-being and reduce mental illness in general among their employees.

However, as an influencing factor from the outside setting, the COVID-19 pandemic, as a critical incident, has been an essential component of the implementation process. The pandemic was the reason for the increased burden on HCWs and their mental well-being and, therefore, for the development of interventions or the adaptation of existing interventions [59–61]. For example, cognitive behavioral therapy (CBT), an evidence-based intervention [62], has been adapted to digital formats to support mental health [59–61]. In contrast, this respiratory virus outbreak could also be considered a factor from the outer setting, influencing the implementation for several reasons. The increased number of infected patients resulted in higher supply requirements, increasing the workload and stressful conditions. Therefore, the employees have less time for themselves and the use of interventions for their mental well-being [45]. Compared with the findings from the systematic review by Pollock et al. [23], none of those studies reported the pandemic as a potential influencing factor. This might suggest that in implementation efforts within past disease outbreaks, the changes in and challenges associated with these pandemic-related conditions did not affect the implementation of those interventions. Further research is needed to investigate how this factor might influence the implementation of mental health interventions from nurses’ and physicians´ perspectives.

In our scoping review, the most significant facilitators were identified from individuals, such as nurses and physicians, and the inner setting, such as hospitals. Our results support the argument of Greenhalgh et al. [63] that recipients of innovations play an active role in the implementation and adoption process. However, our findings showed that during the COVID-19 pandemic, individuals, whether they are potential recipients such as nurses, physicians, or decision-makers, strongly influence the adoption of the intervention in and for the inner setting. Therefore, deliverer-centeredness was considered a facilitator, essential for the institution to esteem the intervention’s potential providers and offer them support. This result is reflected in the findings of Pollock et al. [23] and the implementation strategies, where stakeholder involvement should be considered a critical strategy, primarily for evaluating those interventions [64].

Furthermore, individuals with mental health issues who need support through their organization face an accomplished phenomenon. Stigmatization was another factor in the analysis of influencing factors. Mental health issues were not addressed by society. Interestingly, Pollock et al. [23] did not report a factor that dealt with this topic. In contrast, Graham et al. [18] noted that stigmatization of mental health issues and seeking help were considered barriers to the implementation of digital mental health interventions. Further research is needed on how this factor could be addressed with an appropriate strategy to make nurses and physicians comfortable with interventions that support their mental well-being.

Besides, the implications of these findings for non-pandemic contexts are crucial to achieving not only a sustainable implementation and, thus, utility of PTSD-related interventions for HCWs but also the prevention of their mental well-being. The following implications can be inferred from our research findings:

- Previous analog interventions, like CBT or PFA, were digitalized.
- Interventions had to be adapted rapidly to changing conditions.
- Most adapted interventions were not investigated for effectiveness due to their rapid development and implementation.
- Strategies need to be developed based on the knowledge of which factors could influence the implementation of PTSD-related interventions.
- The management needs to be aware of the mental health problems and needs of employed HCWs.
- The target group of the intervention has to participate in the implementation based on their impact on an efficient implementation.
- In adopting and implementing a PTSD-related intervention, stigmatization as a potential factor has to be considered.

Suppose the management team is aware of the implications and develop tailored implementation strategies to mitigate the potential factors associated with implementing PTSD-related interventions, they might be prepared for future disease outbreaks.

### Limitations

Several strengths and limitations in this scoping review, in terms of the methodology and research results, have to be mentioned.

Since the primary aim of our scoping review was not to investigate the effectiveness of PTSD-related interventions nor the efficacy of their implementation, we did not perform a quality assessment using a risk of bias tool. Furthermore, a research protocol was not developed according to the recommendations of the JBI [24, 25], and it was not registered in the Open Science Framework (OSF). Instead, the eight steps were followed, and prior deliberations were made with other researchers (DH/MR). This highly transparent procedure using the PRISMA-ScR [25] and the Ref Hunter in Web Format [29] offers reproducibility and replicability of the study.

In addition, the results of our scoping review are based on a predefined population and context, as well as interventions, and may not reflect the general population. However, contrary to the assumption that the population and context have to be predefined for identifying potential influencing factors [20], these results might have the opportunity to be transmitted to other healthcare settings or HCWs.

Because of time and other constraints, we used three databases: MEDLINE via PubMed, PsychINFO, and CINAHL via EBSCO. Thus, potentially relevant studies may not have been identified, but they may be minimized by applying supplementary search options such as backward and forward citation tracking and trial registry searches.

Owing to the lack of implementation studies or those that investigated barriers and facilitators, the analysis had to be performed by analyzing themes rather than qualitative quotes or quantitative data. Therefore, the third category for assigning factors that could not be classified as barriers or facilitators was developed. In interpreting the results, attention must be given to the articles from which the influencing factors were analyzed. One factor might have been studied as a facilitator but, from another article, as a neutral factor. Therefore, we presented the results in detail, assigned them to the article, and labeled them according to the respective type of article.

Finally, we did not distinguish between digital and in-person interventions before defining our inclusion criteria. Therefore, the analysis was not designed to identify differences between the factors associated with digital or face-to-face interventions. This could be investigated in further research.

## Conclusion

This study aimed to identify barriers and facilitators that could influence the implementation of interventions for treating PTSD in hospitals. We have been able to answer our research question: *What are the barriers/facilitators in the implementation of PTSD-related interventions for nurses and physicians working in an acute hospital setting during the COVID-19 pandemic? *Several potential factors were identified that might influence the implementation of interventions under pandemic-related conditions. Still, only a few hindering or promoting factors could be determined based on the lack of investigations and empirical data. However, individuals and their inner settings play a crucial role in influencing the adoption of interventions due to pandemic-related challenges. Conceptualized studies with a qualitative or quantitative approach in a formative or summative evaluation are required to investigate the barriers and facilitators in implementing those interventions. Furthermore, we strongly recommend integrating the perspectives of nurses, physicians, and other stakeholders who could influence the implementation of our findings in future research. This approach may enhance the transferability of our findings into the real-world setting.

Besides, future research should focus on identifying tailored implementation strategies to mitigate the barriers and promote the facilitating factors in the implementation of PTSD-related interventions. Accordingly, focusing on methodological investigations by developing rapid and available implementation strategies applicable to a pandemic context might enhance successful implementation. Finally, decision-makers, especially those in healthcare institutions, must evolve an active and planned attitude and use implementation knowledge to implement mental health interventions in their organizations. In addition to being prepared for future disease outbreaks, a focus should be placed on the already high prevalence of PTSD among HCWs. This meant that decision-makers in healthcare institutions had to implement mental health interventions sustainably to support mental well-being and prevent mental health problems.

## Supplementary Information

Additional File 1. Checklist of the PRISMA extension for scoping reviews.

Additional File 2. Research protocol_Interventions.

Additional File 3. Research protocol_Influencing factors.

Additional File 4. Influencing factors.

## Data Availability

No datasets were generated or analysed during the current study.

## Abbreviations

COVID-19: Coronavirus 2019
SARS-CoV-2: Severe acute respiratory syndrome coronavirus type 2
WHO: World Health Organization
HCW: Health care workers
PTSD: Posttraumatic stress disorder
CFIR: Consolidated Framework of Implementation Research
JBI: Joanna Briggs Institute
PRISMA-ScR: PRISMA Extension for Scoping Reviews
ICD-10: International Statistical Classification of Diseases and Related Health Problems 10^th^ Revision
PRESS: Peer Review of Electronic Search Strategies
CBT: Cognitive Behavioral Therapy
OSF: Open Science Framework
