# Supplementary material for: Factors influencing the implementation of interventions for symptoms of posttraumatic stress disorder among hospital-based nurses and physicians during the COVID-19 pandemic: a scoping review
Source: BMC Health Serv Res. 2025 Jul 2;25:885. doi: 10.1186/s12913-025-13005-z (PMC12225526; doi:10.1186/s12913-025-13005-z)
Supplement: Supplementary file 2 — Additional File 2. Research protocol_Interventions. [file 12913_2025_13005_MOESM2_ESM.docx]

***Additional File 2: Research protocol I***

**Research protocol**

**Interventions treating symptoms of post-traumatic stress disorder among hospital-based nurses and physicians during the COVID-19 pandemic**

Content

[List of Tables 5](#_Toc173659940)

[List of Figures 6](#_Toc173659941)

[1. Research question 7](#_Toc173659942)

[2. Inclusion and exclusion criteria 7](#_Toc173659943)

[3. Search strategies 9](#_Toc173659944)

[4. Identification of synonym search terms 9](#_Toc173659945)

[5. Identification of keywords 11](#_Toc173659946)

[6. Search string development 13](#_Toc173659947)

[7. Review search strings 21](#_Toc173659948)

[8. Conducting and documentation of the search 25](#_Toc173659949)

[9. Supplementary search options 35](#_Toc173659950)

[10. Title-Abstract- and full-text-screening 44](#_Toc173659951)

[11. Supplement I Flowchart 45](#_Toc173659952)

[13 Supplement II Bibliographic information of the articles included for data extraction 46](#_Toc173659953)

[14. Research update 50](#_Toc173659954)

[15 References 53](#_Toc173659955)

# List of Tables

[Table 1: Inclusion and exclusion criteria (own visualization) 7](#_Toc173659110)

[Table 2: Synonym search terms (own presentation based on Nordhausen and Hirt (2022)) 9](#_Toc173659111)

[Table 3: Identified key words (own representation based on Nordhausen and Hirt (2022)) 11](#_Toc173659112)

[Table 4: Search string for the database MEDLINE via PubMed (own representation based on Nordhausen and Hirt (2022)) 13](#_Toc173659113)

[Table 5: Search filters (own representation based on Nordhausen and Hirt (2022)) 16](#_Toc173659114)

[Table 6: Rehearsal with the generated search string performed in MEDLINE via PubMed based on Nordhausen and Hirt (2022)) 16](#_Toc173659115)

[Table 7: Search string syntax for MEDLINE via PubMed and Psych INFO via EBSCO (own representation based on Nordhausen and Hirt (2022)) 18](#_Toc173659116)

[Table 8: Search filters (own representation based on Nordhausen and Hirt (2022)) 25](#_Toc173659117)

[Table 9: Documentation of the search (own representation based on Nordhausen and Hirt (2022)) 26](#_Toc173659118)

[Table 10: Documentation of the search in PsychINFO via EBSCO (own representation based on Nordhausen and Hirt (2022)) 30](#_Toc173659119)

[Table 11: Presentation of research findings from the supplementary search in study register (own visualization). 35](#_Toc173659120)

[Table 12: Presentation of results from the backward citation tracking (own visualization). 37](#_Toc173659121)

[Table 13: Results from a 'hand search' in Google scholar based on resulted psychological interventions from a systematic review by Hooper et al. (2021) (own visualization). 37](#_Toc173659122)

[Table 14: Documentation of the search in CINAHL via EBSCO (own representation based on Nordhausen and Hirt (2022)) 39](#_Toc173659123)

[Table 15.Search filters (own representation based on Nordhausen and Hirt (2022)) 50](#_Toc173659124)

# List of Figures

[Figure 1: PRISMA-Flow Chart of the systematic literature search in databases according to Page et al., 2020. 45](#_Toc173659125)

[Figure 2: PRISMA-Flow Chart including the research update according to Page et al., 2020. 50](#_Toc173659126)

# Research question

The aim of this systematic literature research is to identify interventions treating symptoms of post-traumatic stress disorder (PTSD) among nurses and physicians working in an acute hospital setting during the COVID-19 pandemic.

The central research question guiding this systematic literature search is:

*What are the interventions that address symptoms of post-traumatic stress disorder in hospital-based nurses and physicians during the COVID-19 pandemic?*

# Inclusion and exclusion criteria

Based on the **PCC**-elements (**P**opulation, **C**oncept of interest, **C**ontext) (Peters et al., 2022) the in- and exclusion criteria are defined in Table 1 below:

Table 1: Inclusion and exclusion criteria (own visualization)

| Criteria | Definition (inclusion) | Definition (exclusion) |
| --- | --- | --- |
| Population | - Nurses and physicians with symptoms of post-traumatic stress disorder (PTSD) as target population for the evidence-based interventions | - Other professions (e.g., Community Health Nurses, physiotherapist, respiratory therapist) |
| Concept of interest | - Implementation of evidence-based interventions addressing PTSD, specifically one of these symptoms showing in nurses and physicians working at a hospital^1^: - **Intrusion** (e.g., intrusive thoughts, involuntary memories, distressing dreams, flashbacks) - **Avoidance** (e.g., avoiding people, places, activities, objects and situations) - **Alterations in cognition and mood** (e.g., feeling detached or estranged from others, unable to experience positive emotions) - **Alterations in arousal and reactivity** (e.g., insomnia) - Distress (psychological) - Anhedonia - Anxiety - Depression - Suicidal ideations - Acute stress (disorder) - Implementation of evidence-based interventions preventing PTSD by addressing symptoms of PTSD, psychological distress and acute stress disorder | - Non-PTSD related interventions |
| Context | - Studies in acute somatic hospitals during the COVID-19 pandemic period | - Specialized clinics such as mental/ psychiatric hospital |
| Types of evidence sources | - Any kind of study that describes or evaluate evidence-based interventions addressing symptoms of PTSD: - Evaluation studies - Implementation studies - Study protocol - Feasibility studies - Concept articles | - Reviews |
| Other | - Languages: German and English - Year: 2020- 2023 | - Not published before 2020 |

^1^ (Taylor-Desir, 2022; WHO, 2019)

Rationale for in- and exclusion criteria:

*Types of participants*

Physicians and nurses who were working/ work during the COVID-19 pandemic in period from 2019-2023. Couarraze et al. (2021) stated in an international survey that the levels of stress from healthcare professionals was 25.8% higher during the pandemic than the general population.

*Concept*

Saragih et al. (2021) ascertain that during the pandemic globally the prevalence of PTSD in physicians and nurses is as a result from a pooled analysis 49% (95% CI: 22-75%). The researchers included 38 studies in the year 2020, which provided a comprehensive overview of the effects of the pandemic, globally. Across all studies, a total of 53,784 participants were included. Of it, 27,9% were doctors and 43,7% nurses.

*Context*

Context of interest is the COVID-19 pandemic, because this and any kind of disease outbreak could increase risk of mental health problems among healthcare workers in short or long term (Stuijfzand et al., 2020). Only acute somatic hospital-based physicians and nurses are the focus of this study, because they have been caring to a larger extent for patients infected with COVID-19. This kind of environment is associated with a higher prevalence of stress, anxiety, and burnout among staff (Evanoff et al., 2020)

# Search strategies

Based on the PCC-elements, MEDLINE (via PubMed) is chosen because of the wide range of topics in the field of health and the use of research methods. In addition, PsychINFO (via EBSCO) is specialized in psychiatric/ mental health issues, which is necessary for getting all relevant literature related to the objective.

An initial limited search will be conducted in the two databases to identify synonym search terms and key words for each. Second, search strings for each database will be developed to conduct systematic literature research. Third, the search string for MEDLINE (via PubMed), will be verify using the Peer Review of Electronic Search Strategies (PRESS) (McGowan et al., 2016). Fourth, search string for PsychINFO (via EBSCO) will be developed. Finally, further search possibilities as a supplemental limited search in CINAHL (via EBSCO), citation tracking will be conducted (Peters et al., 2020).

# Identification of synonym search terms

Table 2: Synonym search terms (own presentation based on (Nordhausen & Hirt, 2022))

| **Criteria/ search component** | **Search terms** |
| --- | --- |
| **Population** | Physician/Physicians  Medical Doctor  Nurse/Nurses  Nursing staff  Medical staff  Professional Caregivers  Registered nurse  Nurse practitioner  Advanced practice nurse  health care workers  health workers  health-care workers  health care professionals  frontline healthcare workers |
| **Concept of interest** | intervention  post-traumatic stress disorder  PTSD  Acute stress disorder  Psychological Distress |
| **Context** | hospital  acute hospital  clinic  acute setting  hospital setting  COVID-19  COVID-19 pandemic |

Rationale:

*Concept of interest:*

Liang et al. (2020) shows that psychological distress could lead to symptoms of PTSD.

# Identification of keywords

Table 3: Identified key words (own representation based on Nordhausen and Hirt (2022))

| **Search terms** | **Search component** | **keyword (MEDLINE via PubMed)** | **keyword (Psych INFO via EBSCO)** |
| --- | --- | --- | --- |
| Physician  Physicians  Doctor  Physician Assistant  Nurse  Nurses  Nursing staff  Caregivers  Registered nurse  Nurse practitioner  Advanced practice nurse  health care workers  health workers  health-care workers  health care professionals  frontline healthcare workers | **Population** | Nurse Clinicians  Nurse Specialists  Physician Assistants  Nurse Specialists  Nursing Staff, Hospital  Health Personnel | Physicians  Medical Personnel  Clinicians  Nurses  Caregivers  Professional Personnel  Medical Personnel  Frontline Employees  Health Personnel |
| intervention  post-traumatic stress disorder  PTSD  Acute stress disorder  Psychological Distress | **Concept of interest** | Stress Disorders, Post-Traumatic Psychological Distress  Stress Disorders, Traumatic, Acute | Posttraumatic Stress Disorder  Posttraumatic Stress  Stress and Trauma related disorders  Acute Stress Disorder [Acute Stress](javascript:XslPostBack('ctl00$ctl00$MainContentArea$MainContentArea$xslResults','ThesaurusLink','LinkTarget%7CauthorityList%24LinkTerm%7CDE%2B%2522Acute%2BStress%2522');)  Caregiver burden |
| hospital  hospitals  acute hospital  clinic  acute setting  hospital setting  COVID-19  COVID-19 pandemic | **Context** | Hospitals  Subacute care  COVID-19 | Hospitals  Clinics  Treatment Facilities |

**Rationale for keywords in MEDLINE via PubMed**

Context:

*Subacute care* is defined as: *“Medical and skilled nursing services provided to patients who are not in an acute phase of an illness but who require a level of care higher than that provided in a long-term care setting. (JCAHO, Lexikon, 1994)”*. Based on that, in relation to the objective this keyword is included.

# Search string development

According to the recommendation of Nordhausen and Hirt (2022) first the search string for the database MEDLINE via PubMED will be developed. In the subsequent step, this will be tested in a trial run following the recommendation so that an adjustment can be made and thus the effort for the PsychINFO via EBSCO database is minimized.

Table 4: Search string for the database MEDLINE via PubMed (own representation based on Nordhausen and Hirt (2022))

| **Search components** | **Searchstring MEDLINE via PubMed** | **Searchstring PsychINFO via EBSCO** |
| --- | --- | --- |
| **Population** | Physician* [TIAB]  OR  Doctor [TIAB]  OR  “Physician Assistant” [TIAB]  OR  Nurs* [TIAB]  OR  “Nursing staff” [TIAB]  OR  Caregiver* [TIAB]  OR  “Registered nurse” [TIAB]  OR  “Nurse practitioner” [TIAB]  OR  “Advanced practice nurse” [TIAB]  OR  “health care workers” [TIAB]  OR  “health workers” [TIAB]  OR  “health-care workers” [TIAB]  OR  “health care professionals” [TIAB]  OR  “frontline healthcare workers” [TIAB]  OR  “Nurse Clinicians” [MH]  OR  “Nurse Specialists” [MH]  OR  “Physician Assistants” [MH]  OR  “Nurse Specialists” [MH]  OR  “Nursing Staff, Hospital” [MH]  OR  “Health Personnel” [MH] |  |
|  | **AND** | **AND** |
| **Search component 2** | Intervention* [TIAB] |  |
|  | **AND** | **AND** |
|  | “post-traumatic stress disorder” [TIAB]  OR  PTSD [TIAB]  OR  “Acute Stress Disorder” [TIAB]  OR  “Stress Disorders, Post-Traumatic” [MH]  OR  “Psychological Distress” [MH]  OR  “Stress Disorders, Traumatic, Acute” [MH] |  |
|  | **AND** | **AND** |
| **Search component 3** | Hospital* [TIAB]  OR  “acute hospital” [TIAB]  OR  clinic* [TIAB]  OR  “acute setting” [TIAB]  OR  “hospital setting” [TIAB]  OR  Hospitals [MH]  OR  “subacute care” [MH] |  |
|  | **AND** | **AND** |
|  | COVID-19 [TIAB]  OR  “COVID-19 pandemic” [TIAB]  OR  COVID-19 [MH] |  |

The rehearsal with the generated search string was performed on 14^th^ April 2023 in MEDLINE via PubMed (the search string was stored online).

The following search filters were used:

Table 5: Search filters (own representation based on Nordhausen and Hirt (2022))

| **Search filters:** |  |
| --- | --- |
| **Publication Date** | 2020-2023 |
| **Language** | German, English |
| **Spezies** | Humans |

Table 6: Rehearsal with the generated search string performed in MEDLINE via PubMed based on Nordhausen and Hirt (2022))

| Search number | Input | Number of hits |
| --- | --- | --- |
| 1 | Physician* [TIAB] | 455,043 |
| 2 | Doctor [TIAB] | 66,984 |
| 3 | "Physician Assistant" [TIAB] | 1,953 |
| 4 | Nurs* [TIAB] | 527,820 |
| 5 | "Nursing staff" [TIAB] | 14,536 |
| 6 | Caregiver* [TIAB] | 89,622 |
| 7 | "Registered nurse" [TIAB] | 4,290 |
| 8 | "Nurse practitioner" [TIAB] | 6,987 |
| 9 | "Advanced practice nurse" [TIAB] | 1,124 |
| 10 | "health care workers" [TIAB] | 17,148 |
| 11 | "health workers" [TIAB] | 21,641 |
| 12 | "health-care workers" [TIAB] | 17,148 |
| 13 | "health care professionals" [TIAB] | 28,117 |
| 14 | “frontline healthcare workers” [TIAB] | 506 |
| 15 | "Nurse Clinicians" [MH] | 8,506 |
| 16 | "Nurse Specialists" [MH] | 19,488 |
| 17 | "Physician Assistants" [MH] | 6,403 |
| 18 | "Nursing Staff, Hospital" [MH] | 47,860 |
| 19 | "Health Personnel" [MH] | 606,399 |
| 20 | #1 OR #2 OR #3 OR #4 OR #5 OR #6 OR #7 OR #8 OR #9 OR #10 OR #11 OR #12 OR #13 OR #14 OR #15 OR #16 OR #17 OR #18 OR #19 | 1,448,565 |

| Search number | Input | Number of hits |
| --- | --- | --- |
| 21 | Intervention* [TIAB] | 1,303,362 |
| 22 | "post-traumatic stress disorder" [TIAB] | 15,956 |
| 23 | PTSD [TIAB] | 32,920 |
| 24 | "Acute Stress Disorder" [TIAB] | 779 |
| 25 | “Psychological Distress” [TIAB] | 27,502 |
| 26 | “Stress Disorders, Post-Traumatic” [MH] | 40,679 |
| 27 | “Psychological Distress” [MH] | 6,603 |
| 28 | “Stress Disorders, Traumatic, Acute” [MH] | 536 |
| 29 | #22 OR #24 OR #25 OR #26 OR #27 OR #28 | 83,870 |
| 30 | #21 AND #29 | 17,259 |
| 31 | Hospital* [TIAB] | 1,605,107 |
| 32 | "acute hospital" [TIAB] | 4,303 |
| 33 | clinic* [TIAB] | 5,160,437 |
| 34 | "acute setting" [TIAB] | 2,693 |
| 35 | "hospital setting" [TIAB] | 14,161 |
| 36 | Hospitals [MH] | 314,785 |
| 37 | “Subacute care” [MH] | 1,411 |
| 38 | #31 OR #32 OR #33 OR #34 OR #35 OR #36 OR #37 | 6,264,657 |
| 39 | COVID-19 [TIAB] | 307,395 |
| 40 | "COVID-19 pandemic" [TIAB] | 115,894 |
| 41 | COVID-19 [MH] | 217,887 |
| 42 | #39 OR #40 OR #41 | 335,265 |
| 43 | #20 AND #30 OR #38 OR #42 | **192** |
| *Filter* | *Humans, English, German* | **131** |

Adjustment of the search string syntax

Table 7: Search string syntax for MEDLINE via PubMed and Psych INFO via EBSCO (own representation based on Nordhausen and Hirt (2022))

| **Search component** | **Search string MEDLINE via PubMed** | **Search string PsychINFO via EBSCO** |
| --- | --- | --- |
| **Population** | Physician* [TIAB]  OR  Doctor [TIAB]  OR  “Doctor of medicine” [TIAB]  OR  “Physician Assistant” [TIAB]  OR  Nurs* [TIAB]  OR  “Nursing staff” [TIAB]  OR  Caregiver* [TIAB]  OR  “Registered nurse” [TIAB]  OR  “Nurse practitioner” [TIAB]  OR  “Advanced practice nurse” [TIAB]  OR  “health care workers” [TIAB]  OR  “health workers” [TIAB]  OR  “health-care workers” [TIAB]  OR  “health care professionals” [TIAB]  OR  “frontline healthcare workers” [TIAB]  OR  Physicians [MH]  OR  medical staff, hospital [MH]  OR  Nurses [MH]  OR  Nurse Practitioner [MH]  OR  Nursing Staff [MH]  OR  Caregivers [MH]  OR  Nurse Clinicians [MH]  OR  Nurse Specialists [MH]  OR  Physician Assistants [MH]  OR  Nurse Specialists [MH]  OR  Nursing Staff, Hospital [MH]  OR  Health Personnel [MH] | (TI Physician* OR AB Physician* OR TI Doctor OR AB Doctor OR TI “Doctor of medicine” OR AB “Doctor of medicine” OR TI “Physician Assistant” OR AB “Physician Assistant” OR TI Nurs* OR AB Nurs* OR TI “Nursing staff” OR AB “Nursing staff” OR TI Caregiver* OR AB Caregiver* OR TI “Registered nurse” OR AB “Registered nurse” OR TI “Nurse practitioner” OR AB “Nurse practitioner” OR TI “Advanced practice nurse” OR AB “Advanced practice nurse” OR TI “health care workers” OR AB “health care workers” OR TI “health workers” OR AB “health workers” OR TI “health-care workers” OR AB “health-care workers” OR TI “health care professionals” OR AB “health care professionals” OR TI “frontline healthcare workers” OR AB “frontline healthcare workers” OR DE Health Personnel OR DE Physicians OR DE Medical Personnel OR DE Clinicians OR DE Nurses OR DE Caregivers OR DE Professional Personnel OR DE Medical Personnel OR DE Frontline Employees) |
|  | **AND** | **AND** |
|  | Intervention* [TIAB] | (TI intervention* OR AB intervention* OR DE intervention) |
|  | **AND** | **AND** |
| **Concept of interest** | “post-traumatic stress disorder” [TIAB]  OR  PTSD [TIAB]  OR  “Acute Stress Disorder” [TIAB]  OR  “Psychological Distress” [TIAB]  OR  “Stress Disorders, Post-Traumatic” [MH]  OR  “Psychological Distress” [MH]  OR  “Stress Disorders, Traumatic, Acute” [MH] | (TI “post-traumatic stress disorder” OR AB “post-traumatic stress disorder” OR TI PTSD OR AB PTSD OR TI “Acute Stress Disorder” OR AB “Acute Stress Disorder” OR TI “Psychological Distress” OR AB “Psychological Distress” OR DE “Posttraumatic Stress Disorder” OR DE “Posttraumatic Stress” OR DE Trauma OR DE “Acute Stress Disorder” OR DE “Stress and Trauma related disorders” OR DE “Caregiver burden”) |
| **Context** | Hospital* [TIAB]  OR  “acute hospital” [TIAB]  OR  clinic* [TIAB]  OR  “acute setting” [TIAB]  OR  “hospital setting” [TIAB]  OR  Hospitals [MH] | (TI Hospital* OR AB Hospital* OR TI “acute hospital” OR AB “acute hospital” OR TI clinic* OR AB clinic* OR TI “acute setting” OR AB “acute setting” OR TI “hospital setting” OR AB “hospital setting” OR DE Hospitals OR DE Clinics OR DE Treatment Facilities) |
|  | **AND** | **AND** |
|  | COVID-19 [TIAB]  OR  “COVID-19 pandemic” [TIAB]  OR  COVID-19 [MH] | (TI COVID-19 OR AB COVID-19 OR TI “Covid-19 pandemic” OR AB “COVID-19 pandemic” OR DE COVID-19) |

# Review search strings

The creation and review of the search strings for MEDLINE via PubMed and PsychINFO via EBSCO, is done using the Peer Review of Electronic Search Strategies (PRESS). This tool is recommended by Nordhausen and Hirt (2022) for reviewing search strings. The authors describe that not having an external person doing the review resulted in a less fineness. However, they also emphasize that with a high degree of (self-) reflection and conscientiousness, this consequence can be minimized to a large extent (Nordhausen & Hirt, 2022). In addition, the syntax was discussed with the supervisors.

At some points rationales are given to make the decisions behind the answers transparent and to show the reflective approach.

**Search string validate**

Datum: 14^th^ April 2023

Translation research question(s)

Does the search strategy fit my research question?

☒ Yes
 ☐ No

Are the search components clearly defined and delimited from each other?

☒ Yes
 ☐ No

Are there too few or too many search components?

☐ Yes
 ☒ No

*Rationale:*

In component **C**(onsept) it´s important to have to different components to the objective, respectively. Same in component **C**(ontext).

Are the search components too specific or too sensitive?

☐ Yes
 ☒ No

Are there too few or too many search hits?

☐ Yes
 ☒ No

*Rationale:*

With regard to the objective and the number of databases, 92 hits are generated in PubMed (via MEDLINE). The components are specific, but due to the research question.

Operators

Are the operators used correctly?

☒ Yes
 ☐ No
 ☐ Not applicable

Are the brackets in the search string placed correctly?

☐ Yes
 ☒ No
 ☐ Not applicable

*Rationale:*

The particulary search components were not be in brackets ( ).

Is it likely that operator NOT will lead to exclusion of potentially relevant publications?

☐ Yes
 ☐ No
 ☒ Not applicable

*Rationale:*

It was not necessary to use the operator NOT.

Could the use of word spacing operators lead to a more precise search result?

☐ Yes
 ☐ No
 ☒ Not applicable

*Rationale:*

Word spacing operators cannot be used in MEDLINE via PubMed and in PsychINFO via EBSCO lead the use of spacing operators to slightly number of hits.

Were word spacing operators used with an appropriate word spacing number?

☐ Yes
 ☐ No
 ☒ Not applicable

Keywords (MeSH-terms/ Index terms)

Are all keywords relevant?

☐ Yes
 ☒ No
 ☐ Not applicable

Are relevant keywords missing?

☐ Yes
 ☒ No
 ☐ Not applicable

Are subordinate or superordinate keywords too broad or too close?

☐ Yes
 ☒ No
 ☐ Not applicable

Were keywords properly expanded to include underlying keywords?

☒ Yes
 ☐ No
 ☐ Not applicable

*Rationale:*

In MEDLINE via PubMed with the “search command” [MH] all subheadings are included as well as underlying keywords. In PsychINFO via EBSCO every single keyword has to list separate with the “search command” [DE].

Were the subheadings correctly assigned to the keywords?

☒ Yes
 ☐ No
 ☐ Not applicable

Have keywords and catchwords been defined for each search component?

☒ Yes
 ☐ No
 ☐ Not applicable

Keywords

Do all keywords contain all **relevant** word combinations?

☒ Yes
 ☐ No
 ☐ Not applicable

Are all synonyms defined?

☒ Yes
 ☐ No
 ☐ Not applicable

Are placeholders inserted correctly?

☐ Yes
 ☒ No
 ☐ Not applicable

Is the truncation/ “Wildcard” placed too early or too late?

☐ Yes
 ☒ No
 ☐ Not applicable

Are acronyms and abbreviations used appropriately and are the spelled-out terms also integrated?

☒ Yes
 ☐ No
 ☐ Not applicable

Are the keywords too specific or too sensitive?

☐ Yes
 ☒ No
 ☐ Not applicable

Are the “search commands” set correctly?

☒ Yes
 ☐ No
 ☐ Not applicable

Notation, syntax and number of lines

Are there any spelling mistakes?

☐ Yes
 ☒ No

Are there any errors in the syntax?

☒ Yes
 ☐ No

*Rationale:*

Between two keywords the operator [OR] was missing.

Have all search components been integrated into the search string?

☒ Yes
 ☐ No

Limitations and search filters

Were limits and/ or search filters used appropriately and correctly?

☒ Yes

☐ No
 ☐ Not applicable

Are the limitations and/ or search filers indexed in the respective database?

☒ Yes
 ☐ No
 ☐ Not applicable

Are limits and/ or search filters missing?

☐ Yes
 ☒ No
 ☐ Not applicable

*Rationale:*

The criteria “source” leaves open, because of low number of resulted hits in the two databases.

# Conducting and documentation of the search

The search filters listed in the following table are applied for the search in the subject databases MEDLINE via PubMed and CINAHL, partly derived from the inclusion and exclusion criteria:

Table 8: Search filters (own representation based on Nordhausen and Hirt (2022))

| **Search filter** |  |
| --- | --- |
| **Publication Date** | 2019-2023 |
| **Language** | English, German, dutch |
| **Spezies** | Human |

Search in MEDLINE via PubMed conducted at 14^th^ April 2023 (search string is record online)

Table 9: Documentation of the search (own representation based on Nordhausen and Hirt (2022))

| Search component | Search number | Feed | Number of hits | Search string for MEDLINE via PubMed |
| --- | --- | --- | --- | --- |
| Population | 1 | **Physician* [TIAB]** | 455,043 | ((((((((((((((((((Physician* [TIAB]) OR (Doctor [TIAB])) OR ("Physician Assistant" [TIAB])) OR (Nurs* [TIAB])) OR ("Nursing staff" [TIAB])) OR (Caregiver* [TIAB])) OR ("Registered nurse" [TIAB])) OR ("Nurse practitioner" [TIAB])) OR ("Advanced practice nurse" [TIAB])) OR ("health care workers" [TIAB])) OR ("health workers" [TIAB])) OR ("health-care workers" [TIAB])) OR ("health care professionals" [TIAB])) OR ("frontline healthcare workers" [TIAB])) OR ("Nurse Clinicians" [MH])) OR ("Physician Assistants" [MH])) OR ("Nursing Staff, Hospital" [MH])) OR ("Health Personnel" [MH])) OR ("Nurse Specialists" [MH]) |
|  | 2 | **Doctor [TIAB]** | 66,984 |  |
|  | 3 | **"Physician Assistant" [TIAB]** | 1,953 |  |
|  | 4 | **Nurs* [TIAB]** | 527,820 |  |
|  | 5 | **"Nursing staff" [TIAB]** | 14,536 |  |
|  | 6 | **Caregiver* [TIAB]** | 89,622 |  |
|  | 7 | **"Registered nurse" [TIAB]** | 4,290 |  |
|  | 8 | **"Nurse practitioner" [TIAB]** | 6,987 |  |
|  | 9 | **"Advanced practice nurse" [TIAB]** | 1,124 |  |
|  | 10 | **"health care workers" [TIAB]** | 17,148 |  |
|  | 11 | **"health workers" [TIAB]** | 21,641 |  |
|  | 12 | **"health-care workers" [TIAB]** | 17,148 |  |
|  | 13 | **"health care professionals" [TIAB]** | 28,117 |  |
|  | 14 | **"frontline healthcare workers" [TIAB]** | 506 |  |
|  | 15 | **"Nurse Clinicians" [MH]** | 8,506 |  |
|  | 16 | **"Physician Assistants" [MH]** | 6,403 |  |
|  | 17 | **"Nursing Staff, Hospital" [MH]** | 47,860 |  |
|  | 18 | **"Health Personnel" [MH]** | 606,399 |  |
|  | 19 | **"Nurse Specialists" [MH]** | 19,488 |  |
|  | 20 | **#1 OR #2 OR #3 OR #4 OR #5 OR #6 OR #7 OR #8 OR #9 OR #10 OR #11 OR #12 OR #13 OR #14 OR #15 OR #16 OR #17 OR #18 OR #19** | 1,448,565 |  |

| Search component | Search number | Feed | Number of hits | Search string for MEDLINE via PubMed |
| --- | --- | --- | --- | --- |
|  | 21 | **Intervention* [TIAB]** | 1,303,632 |  |
| Concept of interest | 22 | **"post-traumatic stress disorder" [TIAB]** | 15,956 | (((((("post-traumatic stress disorder" [TIAB]) OR (PTSD [TIAB])) OR ("Acute Stress Disorder" [TIAB])) OR ("Psychological Distress" [TIAB])) OR ("Stress Disorders, Post-Traumatic" [MH])) OR ("Psychological Distress" [MH])) OR ("Stress Disorders, Traumatic, Acute" [MH]) |
|  | 23 | **PTSD [TIAB]** | 32,920 |  |
|  | 24 | **"Acute Stress Disorder" [TIAB]** | 779 |  |
|  | 25 | **"Psychological Distress" [TIAB]** | 27,502 |  |
|  | 26 | **"Stress Disorders, Post-Traumatic" [MH]** | 40,679 |  |
|  | 27 | **"Psychological Distress" [MH]** | 6,603 |  |
|  | 28 | **"Stress Disorders, Traumatic, Acute" [MH]** | 536 |  |
|  | 29 | #22 OR #23 OR #24 OR #25 OR #26 OR #27 OR #28 | 83,870 |  |
|  | 30 | #21 AND #29 | 17,259 | (Intervention* [TIAB]) AND ((((((("post-traumatic stress disorder" [TIAB]) OR (PTSD [TIAB])) OR ("Acute Stress Disorder" [TIAB])) OR ("Psychological Distress" [TIAB])) OR ("Stress Disorders, Post-Traumatic" [MH])) OR ("Psychological Distress" [MH])) OR ("Stress Disorders, Traumatic, Acute" [MH])) |

| Search components | Search number | feed | Number of hits | Search string for MEDLINE via PubMed |
| --- | --- | --- | --- | --- |
| Context | 31 | **Hospital* [TIAB]** | 1,605,107 | ((((((Hospital* [TIAB]) OR ("acute hospital" [TIAB])) OR (clinic* [TIAB])) OR ("acute setting" [TIAB])) OR ("hospital setting" [TIAB])) OR (Hospitals [MH])) OR ("Subacute care" [MH]) |
|  | 32 | **"acute hospital" [TIAB]** | 4,303 |  |
|  | 33 | **clinic* [TIAB]** | 5,160,437 |  |
|  | 34 | **"acute setting" [TIAB]** | 2,693 |  |
|  | 35 | **"hospital setting" [TIAB]** | 14,161 |  |
|  | 36 | **Hospitals [MH]** | 314,785 |  |
|  | 37 | **"Subacute care" [MH]** | 1,411 |  |
|  | 38 | **#31 OR #32 OR #33 OR #34 OR #35 OR #36 OR #37** | 6,264,657 |  |
|  | 39 | **COVID-19 [TIAB]** | 307,395 |  |
|  | 40 | **"COVID-19 pandemic" [TIAB]** | 115,894 |  |
|  | 41 | **COVID-19 [MH]** | 217,887 |  |
|  | 42 | **#39 OR #40 OR #41** | 335,265 | ((COVID-19 [TIAB]) OR ("COVID-19 pandemic" [TIAB])) OR (COVID-19 [MH]) |
|  | 43 | **#20 AND #30 AND #38 AND #42** | **192** | (((((((((((((((((((((Physician* [TIAB]) OR (Doctor [TIAB])) OR ("Physician Assistant" [TIAB])) OR (Nurs* [TIAB])) OR ("Nursing staff" [TIAB])) OR (Caregiver* [TIAB])) OR ("Registered nurse" [TIAB])) OR ("Nurse practitioner" [TIAB])) OR ("Advanced practice nurse" [TIAB])) OR ("health care workers" [TIAB])) OR ("health workers" [TIAB])) OR ("health-care workers" [TIAB])) OR ("health care professionals" [TIAB])) OR ("frontline healthcare workers" [TIAB])) OR ("Nurse Clinicians" [MH])) OR ("Physician Assistants" [MH])) OR ("Nursing Staff, Hospital" [MH])) OR ("Health Personnel" [MH])) OR ("Nurse Specialists" [MH])) AND ((Intervention* [TIAB]) AND ((((((("post-traumatic stress disorder" [TIAB]) OR (PTSD [TIAB])) OR ("Acute Stress Disorder" [TIAB])) OR ("Psychological Distress" [TIAB])) OR ("Stress Disorders, Post-Traumatic" [MH])) OR ("Psychological Distress" [MH])) OR ("Stress Disorders, Traumatic, Acute" [MH])))) AND (((((((Hospital* [TIAB]) OR ("acute hospital" [TIAB])) OR (clinic* [TIAB])) OR ("acute setting" [TIAB])) OR ("hospital setting" [TIAB])) OR (Hospitals [MH])) OR ("Subacute care" [MH]))) AND (((COVID-19 [TIAB]) OR ("COVID-19 pandemic" [TIAB])) OR (COVID-19 [MH])) |

| Search component | Search number | Feed | Number of hits | Search string for MEDLINE via PubMed |
| --- | --- | --- | --- | --- |
|  | 44 | ***#43 AND Filter: Humans, English, German*** | **131** | (((((((((((((((((((((Physician* [TIAB]) OR (Doctor [TIAB])) OR ("Physician Assistant" [TIAB])) OR (Nurs* [TIAB])) OR ("Nursing staff" [TIAB])) OR (Caregiver* [TIAB])) OR ("Registered nurse" [TIAB])) OR ("Nurse practitioner" [TIAB])) OR ("Advanced practice nurse" [TIAB])) OR ("health care workers" [TIAB])) OR ("health workers" [TIAB])) OR ("health-care workers" [TIAB])) OR ("health care professionals" [TIAB])) OR ("frontline healthcare workers" [TIAB])) OR ("Nurse Clinicians" [MH])) OR ("Physician Assistants" [MH])) OR ("Nursing Staff, Hospital" [MH])) OR ("Health Personnel" [MH])) OR ("Nurse Specialists" [MH])) AND ((Intervention* [TIAB]) AND ((((((("post-traumatic stress disorder" [TIAB]) OR (PTSD [TIAB])) OR ("Acute Stress Disorder" [TIAB])) OR ("Psychological Distress" [TIAB])) OR ("Stress Disorders, Post-Traumatic" [MH])) OR ("Psychological Distress" [MH])) OR ("Stress Disorders, Traumatic, Acute" [MH])))) AND (((((((Hospital* [TIAB]) OR ("acute hospital" [TIAB])) OR (clinic* [TIAB])) OR ("acute setting" [TIAB])) OR ("hospital setting" [TIAB])) OR (Hospitals [MH])) OR ("Subacute care" [MH]))) AND (((COVID-19 [TIAB]) OR ("COVID-19 pandemic" [TIAB])) OR (COVID-19 [MH])) Filters: English, German, Humans |

Search in PsychINFO via EBSCO conducted at 16^th^ April 2023 (search string is record online)

Table 10: Documentation of the search in PsychINFO via EBSCO (own representation based on Nordhausen and Hirt (2022))

| Search component | Search number | Feed | Number of hits | Search string for PsychINFO via EBSCO |
| --- | --- | --- | --- | --- |
| Population | 1 | TI Physician* | 12,438 |  |
|  | 2 | AB Physician* | 67,087 |  |
|  | 3 | TI Doctor | 4,694 |  |
|  | 4 | AB Doctor | 29,205 |  |
|  | 5 | TI “Doctor of medicine” | 8 |  |
|  | 6 | AB “Doctor of medicine” | 55 |  |
|  | 7 | TI “Physician Assistant” | 113 |  |
|  | 8 | AB “Physician Assistant” | 278 |  |
|  | 9 | TI Nurs* | 47,624 |  |
|  | 10 | AB Nurs* | 109,210 |  |
|  | 11 | TI “Nursing staff” | 577 |  |
|  | 12 | AB “Nursing staff” | 3,688 |  |
|  | 13 | TI Caregiver* | 17,046 |  |
|  | 14 | AB Caregiver* | 57,076 |  |
|  | 15 | TI “Registered nurse” | 246 |  |
|  | 16 | AB “Registered nurse” | 1,134 |  |
|  | 17 | TI “Nurse practitioner” | 506 |  |
|  | 18 | AB “Nurse practitioner” | 1,260 |  |
|  | 19 | TI "Advanced practice nurse” | 53 |  |
|  | 20 | AB “Advanced practice nurse” | 231 |  |
|  | 21 | TI “health care workers” | 539 |  |

| Search component | Search number | Feed | Number of hits | Search string for PsychINFO via EBSCO |
| --- | --- | --- | --- | --- |
| Population | 22 | AB “health care workers” | 2,438 | (TI Physician* OR AB Physician* OR TI Doctor OR AB Doctor OR TI “Doctor of medicine” OR AB “Doctor of medicine” OR TI “Physician Assistant” OR AB “Physician Assistant” OR TI Nurs* OR AB Nurs* OR TI “Nursing staff” OR AB “Nursing staff” OR TI Caregiver* OR AB Caregiver* OR TI “Registered nurse” OR AB “Registered nurse” OR TI “Nurse practitioner” OR AB “Nurse practitioner” OR TI “Advanced practice nurse” OR AB “Advanced practice nurse” OR TI “health care workers” OR AB “health care workers” OR TI “health workers” OR AB “health workers” OR TI “health-care workers” OR AB “health-care workers” OR TI “health care professionals” OR AB “health care professionals” OR DE Health Personnel OR DE Physicians OR DE Medical Personnel OR DE Clinicians OR DE Nurses OR DE Caregivers OR DE Professional Personnel OR DE Medical Personnel OR DE Frontline Employees) |
|  | 23 | TI “health workers” | 980 |  |
|  | 24 | AB “health workers” | 4,969 |  |
|  | 25 | TI “health-care workers” | 539 |  |
|  | 26 | AB “health-care workers” | 2,438 |  |
|  | 27 | TI “health care professionals” | 944 |  |
|  | 28 | AB “health care professionals” | 9,251 |  |
|  | 29 | TI “frontline healthcare workers” | 41 |  |
|  | 30 | AB “frontline healthcare workers” | 84 |  |
|  | 31 | DE Health Personnel | 26,167 |  |
|  | 32 | DE Physicians | 27,111 |  |
|  | 33 | DE Medical Personnel | 6,579 |  |
|  | 34 | DE Clinicians | 13,184 |  |
|  | 35 | DE Nurses | 33,523 |  |
|  | 36 | DE Caregivers | 41,950 |  |
|  | 37 | DE Professional Personnel | 5,534 |  |
|  | 38 | DE Medical Personnel | 6,579 |  |
|  | 39 | DE Frontline Employees | 208 |  |
|  | 40 | #1 OR #2 OR #3 OR #4 Or #5 Or #6 Or #7 OR #8 Or #9 OR #10 OR #11 OR #12 OR #13 OR #14 Or #15 Or #16 Or #17 OR #18 OR #19 Or #20 OR #21 OR #22 OR #23 OR #24 OR #25 OR #26 OR #27 OR #28 OR #29 OR #30 OR #31 OR #32 OR #33 OR #34 OR #35 OR #36 OR #37 OR 38 OR #39 | 307,441 |  |

| Search component | Search number | Feed | Number of hits | Search string for PsychINFO via EBSCO |
| --- | --- | --- | --- | --- |
| Concept of interest | 41 | TI intervention* | 86,159 | (TI intervention* OR AB intervention* OR DE Intervention) |
|  | 42 | AB intervention* | 449,618 |  |
|  | 43 | DE Intervention | 86,100 |  |
|  | 44 | #41 OR #42 OR #43 | 466,676 |  |
|  | 45 | TI “post-traumatic stress disorder” | 4,037 | (TI “post-traumatic stress disorder” OR AB “post-traumatic stress disorder” OR TI PTSD OR AB PTSD OR TI “Acute Stress Disorder” OR AB “Acute Stress Disorder” OR TI “Psychological Distress” OR AB “Psychological Distress” OR DE “Posttraumatic Stress Disorder” OR DE “Posttraumatic Stress” OR DE Trauma OR DE “Acute Stress Disorder” OR DE “Stress and Trauma related disorders” OR DE “Caregiver burden”) |
|  | 46 | AB “post-traumatic stress disorder” | 11,121 |  |
|  | 47 | TI PTSD | 8,833 |  |
|  | 48 | AB PTSD | 37,612 |  |
|  | 49 | TI “Acute Stress Disorder” | 270 |  |
|  | 50 | AB “Acute Stress Disorder” | 831 |  |
|  | 51 | TI “Psychological Distress” | 5,815 |  |
|  | 52 | AB “Psychological Distress” | 21,977 |  |
|  | 53 | DE “Posttraumatic Stress Disorder” | 39,317 |  |
|  | 54 | DE “Posttraumatic Stress” | 1,649 |  |
|  | 55 | DE Trauma | 28,905 |  |
|  | 56 | DE “Acute Stress Disorder” | 676 |  |
|  | 57 | DE “Stress and Trauma related disorders” | 48 |  |
|  | 58 | DE “Caregiver burden” | 7,090 |  |
|  | 59 | #45 OR #46 OR #47 OR #48 OR #49 OR #50 OR #51 OR #52 OR #53 OR #54 OR #55 OR #56 OR #57 OR #58 | **99,933** |  |

| Search component | Search number | Feed | Number of hits | Search string for PsychINFO via EBSCO |
| --- | --- | --- | --- | --- |
|  | 60 | #44 AND #59 | **20,547** | ((TI intervention* OR AB intervention* OR DE intervention) AND (TI “post-traumatic stress disorder” OR AB “post-traumatic stress disorder” OR TI PTSD OR AB PTSD OR TI “Acute Stress Disorder” OR AB “Acute Stress Disorder” OR TI “Psychological Distress” OR AB “Psychological Distress” OR DE “Posttraumatic Stress Disorder” OR DE “Posttraumatic Stress” OR DE Trauma OR DE “Acute Stress Disorder” OR DE “Stress and Trauma related disorders” OR DE “Caregiver burden”)) |
| Context | 61 | TI Hospital* | 36,240 | (TI Hospital* OR AB Hospital* OR TI “acute hospital” OR AB “acute hospital” OR TI clinic* OR AB clinic* OR TI “acute setting” OR AB “acute setting” OR TI “hospital setting” OR AB “hospital setting” OR DE Hospitals OR DE Clinics OR DE Treatment Facilities) |
|  | 62 | AB Hospital* | 169,250 |  |
|  | 63 | TI “acute hospital” | 223 |  |
|  | 64 | AB “acute hospital” | 830 |  |
|  | 65 | TI clinic* | 112,740 |  |
|  | 66 | AB clinic* | 675,499 |  |
|  | 67 | TI “acute setting” | 31 |  |
|  | 68 | AB “acute setting” | 263 |  |
|  | 69 | TI “hospital setting” | 676 |  |
|  | 70 | AB “hospital setting” | 2,632 |  |
|  | 71 | DE Hospitals | 19,101 |  |
|  | 72 | DE Clinics | 6,912 |  |
|  | 73 | DE Treatment Facilities | 2,132 |  |
|  | 74 | #61 OR #62 OR #63 OR #64 OR #65 OR #66 OR #67 #OR #68 OR #69 OR #70 OR #71 OR #72 OR #73 | **822,818** |  |
| Context | 78 | TI COVID-19 | 21,000 | (TI COVID-19 OR AB COVID-19 OR TI “COVID-19 pandemic” OR AB “COVID-19 pandemic” OR DE COVID-19) |
|  | 79 | AB COVID-19 | 28,509 |  |
|  | 80 | TI “COVID-19 pandemic” | 7,952 |  |
|  | 81 | AB “COVID-19 pandemic” | 16,646 |  |
|  | 82 | DE COVID-19 | 21,216 |  |
|  | 83 | #78 OR #79 OR #80 OR #81 OR #82 | **30,402** |  |
| Search component | **Search number** | **Feed** | **Number of hits** | **Search string for PsychINFO via EBSCO** |
|  | 84 | #40 AND #60 AND #74 AND #83 | **83** | ((TI Physician* OR AB Physician* OR TI Doctor OR AB Doctor OR TI “Doctor of medicine” OR AB “Doctor of medicine” OR TI “Physician Assistant” OR AB “Physician Assistant” OR TI Nurs* OR AB Nurs* OR TI “Nursing staff” OR AB “Nursing staff” OR TI Caregiver* OR AB Caregiver* OR TI “Registered nurse” OR AB “Registered nurse” OR TI “Nurse practitioner” OR AB “Nurse practitioner” OR TI “Advanced practice nurse” OR AB “Advanced practice nurse” OR TI “health care workers” OR AB “health care workers” OR TI “health workers” OR AB “health workers” OR TI “health-care workers” OR AB “health-care workers” OR TI “health care professionals” OR AB “health care professionals” OR DE Health Personnel OR DE Physicians OR DE Medical Personnel OR DE Clinicians OR DE Nurses OR DE Caregivers OR DE Professional Personnel OR DE Medical Personnel OR DE Frontline Employees) AND ((TI intervention* OR AB intervention* OR DE intervention) AND (TI “post-traumatic stress disorder” OR AB “post-traumatic stress disorder” OR TI PTSD OR AB PTSD OR TI “Acute Stress Disorder” OR AB “Acute Stress Disorder” OR TI “Psychological Distress” OR AB “Psychological Distress” OR DE “Posttraumatic Stress Disorder” OR DE “Posttraumatic Stress” OR DE Trauma OR DE “Acute Stress Disorder” OR DE “Stress and Trauma related disorders” OR DE “Caregiver burden”)) AND (TI Hospital* OR AB Hospital* OR TI “acute hospital” OR AB “acute hospital” OR TI clinic* OR AB clinic* OR TI “acute setting” OR AB “acute setting” OR TI “hospital setting” OR AB “hospital setting” OR DE Hospitals OR DE Clinics OR DE Treatment Facilities) AND (TI COVID-19 OR AB COVID-19 OR TI “COVID-19 pandemic” OR AB “COVID-19 pandemic” OR DE COVID-19)) |
|  | 85 | AND *Filter: 2020-2023, Humans, English, German* | **83** |  |

# Supplementary search options

This literature research is sensitive and aimed to identify preferably all possible references regarding the research question. Therefore Cooper et al. (2017) conclude, that several supplementary search option should be conducted after the systematic research in databases.

This research focused on the topic of which interventions exist for symptoms of PTSD among hospitals-based nurses and physicians during the COVID-19 pandemic. First a limited systematic search is conducted in CINHAL via EBSCO, because this database contains literature specifically for the field literature from nursing science and related topics from the health care sector (see Table 10). Furthermore, the publications exported to EndNote 20 and searched for duplicates before the additional Title-Abstract-Screening is conducted in Rayyan (Ouzzani et al., 2016). After that a total of two studies were included in full text screening (see Supplement I).

Secondly, during the full text-screening of all included studies, the backwards citation tracking was conducted to search in the reference list of each included study for potential publications (e.g., study protocols) regarding the research question.

Thirdly, a search in study register (PROSEPRO, OSF, World Health Organization International Clinical Trials, EU Clinical Trials Register, German Register clinical trial, study register from U.S. National Institute of Health and ICTRN-study register for clinical trials in all fields of health care).

24^th^ April 2023 the following findings resulted from this research in the study register from the U.S. National Institute of Health and ICTRN-study register for clinical trials in all fields of health care:

Table 11: Presentation of research findings from the supplementary search in study register (own visualization).

| **Title** | **Researchers** | **Duration** | **Content** | **Status** |
| --- | --- | --- | --- | --- |
| **Study register from the U.S. National Institute of Health** | | | | |
| Reducing Posttraumatic Stress Disorder (PTSD) Symptoms in Frontline Health Care Workers | Garlick, James, Hemphill, Naomi | April 2023 until March 2026 | Study address symptoms of PTSD in health care workers after the COVID-19 pandemic.  Hypothesis is, that the Prolonged Exposure for Primary Care (PE-PC) will reduce PTSD symptoms and improve functioning, compared to EAP Treatment as Usual (TAU). | Recruiting phase |
| Health cAre woRkers exposed to COVID-19 (HARD COVID 19) | Responsible: University Hospital, Tours | From November 2020 until? | Evaluation of an intervention adapted to the exceptional circumstances of the crisis, like Depression, Burnout, Post Traumatic Stress Disorder. It´s designed as Trial(s) within Cohort design (TWIC). Intervention: Desensitization and neuro-emotional integration by eye movements (EMDR). | Active, not recruiting |
| Beyond Silence: Advancing E-mental Health Solutions to Support Canadian Healthcare Workers | Responsible: McMaster University, Canada | August 2022, estimated Study Completion: August 2023 | Purpose is to scale implementation and evaluation of an m-Health app dsigned to promote early intervention and mental health support for frontline healthcare workers to reduce the risk of PTSD and/ or the mental health impact of the COVID-19 pandemic. | Not yet recruiting |
| Creative Arts Program to Reduce Burnout in Healthcare Professionals (CORAL) | Responsible: University of Colorado, Denver | March 2020 until March 2025 | Question is, if creative arts programs that include visual, musical, written, or physical expression can reduce symptoms of burnout syndrome, Post Traumatic Stress Disorder (PTSD), depression, and anxiety in critical care healthcare professionals. | Recruiting |
| **ICTRN-study register for clinical trials in all fields of health care** | | | | |
| Supporting healthcare workers with cognitive and behavioral coaching for posttraumatic stress disorder and depression. | Oxford Centre for Anxiety Disorders and Trauma | October 2022 until March 2025 | Intervention: SHAPE is a brief, accessible, remotely delivered intervention that targets cognitive processes and behaviors that maintain PTSD and depression. Aim is to evaluate SHAPE. Participants: healthcare workers (e.g. doctors and nurses) | Recruiting |

Results from the backward citation tracking

Table 12: Presentation of results from the backward citation tracking (own visualization).

| **In…** | **Result** |
| --- | --- |
| Buselli et al. (2021) | Morina et al. (2021)  Mellins et al. (2020)  Albott et al. (2020)  Lefevre et al. (2021) |
| Hooper et al. (2021) | Blake et al. (2020) |
| Serrano-Ripoll (2020) | Fiol-DeRoque et al. (2021) |

Other research options

Results from a systematic review by Hooper et al. (2021) show, that some of the presented early psychological interventions were specifically developed to prevent or reduce symptoms of post-traumatic stress disorder (PTSD). For example, the ‘Eye Movement and desensitization and reprocessing intervention’ (EMDR) or the ‘Psychological First Aid’(PFA).

In this case, the backwards citation tracking did not lead to any useful studies related to the research objectives. So, the researchers decided to conduct a web search via Google scholar with a combination of the following search terms:

- *name of the psychological intervention* mentioned in the systematic review by Hooper et al. (2021)
- *PTSD*
- *health-care workers*
- *COVID-19*

For these psychological interventions, the following studies met the inclusion criteria:

Table 13: Results from a 'hand search' in Google scholar based on resulted psychological interventions from a systematic review by Hooper et al. (2021) (own visualization).

| *Psychological First Aid (PFA)* | Wang et al. (2022)  Sulaiman et al. (2020) |
| --- | --- |
| *Eye Movement Desensitization and Processing (EMDR)* | Fogliato et al. (2022)  Sagaltici et al. (2022) |

Search in CINAHL via EBSCO conducted at 18^th^ April 2023 (search string is record online)

Table 14: Documentation of the search in CINAHL via EBSCO (own representation based on Nordhausen and Hirt (2022))

| Search component | Search number | Feed | Number of hits | Search string for CINAHL via EBSCO |
| --- | --- | --- | --- | --- |
| Population | 1 | TI Physician* | 45,665 |  |
|  | 2 | AB Physician* | 146,113 |  |
|  | 3 | TI Doctor | 22,055 |  |
|  | 4 | AB Doctor | 49,049 |  |
|  | 5 | TI “Doctor of medicine” | 7 |  |
|  | 6 | AB “Doctor of medicine” | 212 |  |
|  | 7 | TI “Physician Assistant” | 1,085 |  |
|  | 8 | AB “Physician Assistant” | 1,272 |  |
|  | 9 | TI Nurs* | 368,585 |  |
|  | 10 | AB Nurs* | 387,874 |  |
|  | 11 | TI “Nursing staff” | 2,201 |  |
|  | 12 | AB “Nursing staff” | 11,345 |  |
|  | 13 | TI Caregiver* | 23,889 |  |
|  | 14 | AB Caregiver* | 54,817 |  |
|  | 15 | TI “Registered nurse” | 1,648 |  |
|  | 16 | AB “Registered nurse” | 4,886 |  |
|  | 17 | TI “Nurse practitioner” | 4,428 |  |
|  | 18 | AB “Nurse practitioner” | 5,606 |  |
|  | 19 | TI "Advanced practice nurse” | 469 |  |
|  | 20 | AB “Advanced practice nurse” | 879 |  |
|  | 21 | TI “health care workers” | 2,597 |  |

| Search component | Search number | Feed | Number of hits | Search string for CINAHL via EBSCO |
| --- | --- | --- | --- | --- |
|  | 22 | AB “health care workers” | 6,691 | (TI Physician* OR AB Physician* OR TI Doctor OR AB Doctor OR TI “Doctor of medicine” OR AB “Doctor of medicine” OR TI “Physician Assistant” OR AB “Physician Assistant” OR TI Nurs* OR AB Nurs* OR TI “Nursing staff” OR AB “Nursing staff” OR TI Caregiver* OR AB Caregiver* OR TI “Registered nurse” OR AB “Registered nurse” OR TI “Nurse practitioner” OR AB “Nurse practitioner” OR TI “Advanced practice nurse” OR AB “Advanced practice nurse” OR TI “health care workers” OR AB “health care workers” OR TI “health workers” OR AB “health workers” OR TI “health-care workers” OR AB “health-care workers” OR TI “health care professionals” OR AB “health care professionals” OR TI “frontline healthcare workers” OR AB “frontline healthcare workers” OR MH Physicians OR MH “Physician Assistants” OR MH Nurses OR MH "Practical Nurses" OR MH "Nursing Staff, Hospital" OR MH "Staff Nurses" OR MH Caregivers OR MH "Registered Nurses" OR MH "Practical Nurses" OR MH "Nurse Practitioners" OR MH "Advanced Practice Nurses" OR MH "Health Personnel") |
|  | 23 | TI “health workers” | 2,735 |  |
|  | 24 | AB “health workers” | 9,058 |  |
|  | 25 | TI “health-care workers” | 2,597 |  |
|  | 26 | AB “health-care workers” | 6,691 |  |
|  | 27 | TI “health care professionals” | 2,308 |  |
|  | 28 | AB “health care professionals” | 17,584 |  |
|  | 29 | TI “frontline healthcare workers” | 50 |  |
|  | 30 | AB “frontline healthcare workers” | 143 |  |
|  | 31 | MH Physicians | 66,776 |  |
|  | 32 | MH "Physician Assistants" | 5,675 |  |
|  | 33 | MH Nurses | 69,672 |  |
|  | 34 | MH "Practical Nurses" | 5,857 |  |
|  | 35 | MH "Nursing Staff, Hospital" | 24,553 |  |
|  | 36 | MH "Staff Nurses" | 8,213 |  |
|  | 37 | MH Caregivers | 42,752 |  |
|  | 38 | MH "Registered Nurses" | 36,065 |  |
|  | 39 | MH "Practical Nurses" | 5,857 |  |
|  | 40 | MH "Nurse Practitioners" | 20,139 |  |
|  | 41 | MH "Advanced Practice Nurses" | 1,989 |  |
|  | 42 | MH "Health Personnel" | 51,350 |  |
|  | 43 | #1 OR #2 OR #3 OR #4 Or #5 Or #6 Or #7 OR #8 Or #9 OR #10 OR #11 OR #12 OR #13 OR #14 Or #15 Or #16 Or #17 OR #18 OR #19 Or #20 OR #21 OR #22 OR #23 OR #24 OR #25 OR #26 OR #27 OR #28 OR #29 OR #30 OR #31 OR #32 OR #33 OR #34 OR #35 OR #36 OR #37 OR 38 OR #39 OR #40 OR #41 OR #42 | **997,988** |  |

| Search component | Search number | Feed | Number of hits | Search string for CINAHL via EBSCO |
| --- | --- | --- | --- | --- |
| Concept of interest | 44 | TI intervention* | 118,385 | (TI intervention* OR AB intervention*) |
|  | 45 | AB intervention* | 504,651 |  |
|  | 46 | #44 OR #45 | 551,649 |  |
|  | 47 | TI “post-traumatic stress disorder” | 2,250 | (TI “post-traumatic stress disorder” OR AB “post-traumatic stress disorder” OR TI PTSD OR AB PTSD OR TI “Acute Stress Disorder” OR AB “Acute Stress Disorder” OR TI “Psychological Distress” OR AB “Psychological Distress” OR MH "Stress Disorders, Post-Traumatic" OR MH "Psychological Distress") |
|  | 48 | AB “post-traumatic stress disorder” | 5,845 |  |
|  | 49 | TI PTSD | 4,332 |  |
|  | 50 | AB PTSD | 12,387 |  |
|  | 51 | TI “Acute Stress Disorder” | 124 |  |
|  | 52 | AB “Acute Stress Disorder” | 288 |  |
|  | 53 | TI “Psychological Distress” | 4,224 |  |
|  | 54 | AB “Psychological Distress” | 13,142 |  |
|  | 55 | MH "Stress Disorders, Post-Traumatic" | 26,951 |  |
|  | 56 | MH "Psychological Distress" | 4,783 |  |
|  | 57 | #47 OR #48 OR #49 OR #50 OR #51 OR #52 OR #53 OR #54 OR #55 OR #56 OR | **47,113** |  |
|  | 58 | #46 AND #57 | **9,678** |  |

| Search component | Search number | Feed | Number of hits | Search string for CINAHL via EBSCO |
| --- | --- | --- | --- | --- |
| Context | 59 | TI Hospital* | 161,045 |  |
|  | 60 | AB Hospital* | 478,899 |  |
|  | 61 | TI “acute hospital” | 872 |  |
|  | 62 | AB “acute hospital” | 2,677 |  |
|  | 63 | TI clinic* | 339,738 |  |
|  | 64 | AB clinic* | 1,135,481 |  |
|  | 65 | TI “acute setting” | 169 |  |
|  | 66 | AB “acute setting” | 966 |  |
|  | 67 | TI “hospital setting” | 1,876 |  |
|  | 68 | AB “hospital setting” | 5,872 |  |
|  | 69 | MH Hospitals | 67,009 |  |
|  | 70 | OR #59 OR #60 OR #61 OR #62 OR #63 OR #64 OR #65 OR #66 OR #67 #OR #68 OR #69 | **1,715,286** | (TI Hospital* OR AB Hospital* OR TI “acute hospital” OR AB “acute hospital” OR TI clinic* OR AB clinic* OR TI “acute setting” OR AB “acute setting” OR TI “hospital setting” OR AB “hospital setting” OR MH Hospitals) |
| Context | 71 | TI COVID-19 | 84,312 | (TI COVID-19 OR AB COVID-19 OR TI “COVID-19 pandemic” OR AB “COVID-19 pandemic” OR MH “COVID-19”) |
|  | 72 | AB COVID-19 | 69,800 |  |
|  | 73 | TI “COVID-19 pandemic” | 20,575 |  |
|  | 74 | AB “COVID-19 pandemic” | 31,900 |  |
|  | 75 | MH „COVID-19“ | 40,295 |  |
|  | 76 | #71 OR #72 OR #73 OR #74 OR #75 | **115,144** |  |
|  | 77 | #43 AND #58 AND #70 AND #76 | **61** |  |
|  | 78 | AND *Filter: 2020-2023, Humans, English, German* | **59** |  |

# Title-Abstract- and full-text-screening

All included references were export to EndNote 20 and after a screening for duplicates, the references were transferred to Rayyan (Ouzzani et al., 2016). There, the title-abstract-, as well as the full-text-screening took place by the first author herself.

To establish methodological quality, four studies were independently reviewed MR and DH, each. On 18^th^ April 2023 the results were discussed and one reference, which was set on “maybe” by DK was included after a discussion.

From 19^th^ until 21^th^April 2023 the full-text-screening was conducted also in Rayyan by DK and three references were screened by MR and DH, independently.

The Discussion took place on 4^th^ April 2023, as well as the alignment with the MR and DH.

# Supplement I Flowchart

Figure 1: PRISMA-Flow Chart of the systematic literature search in databases according to Page et al., 2020.

# 13 Supplement II Bibliographic information of the articles included for data extraction

1. Albott, C. S., Wozniak, J. R., McGlinch, B. P., Wall, M. H., Gold, B. S., & Vinogradov, S. (2020). Battle Buddies: Rapid Deployment of a Psychological Resilience Intervention for Health Care Workers During the COVID-19 Pandemic. *Anesth Analg*, *131*(1), 43-54. <https://doi.org/10.1213/ANE.0000000000004912>
2. Blake, H., Bermingham, F., Johnson, G., & Tabner, A. (2020). Mitigating the Psychological Impact of COVID-19 on Healthcare Workers: A Digital Learning Package. *Int J Environ Res Public Health*, *17*(9). <https://doi.org/10.3390/ijerph17092997>
3. Bureau, R., Bemmouna, D., Faria, C. G. F., Goethals, A.-A. C., Douhet, F., Mengin, A. C., Fritsch, A., Zinetti Bertschy, A., Frey, I., & Weiner, L. (2021). My health too: Investigating the feasibility and the acceptability of an internet-based cognitive-behavioral therapy program developed for healthcare workers. *Frontiers in Psychology*, *12*. <https://doi.org/10.3389/fpsyg.2021.760678>
4. Dong, L., Meredith, L. S., Farmer, C. M., Ahluwalia, S. C., Chen, P. G., Bouskill, K., Han, B., Qureshi, N., Dalton, S., Watson, P., Schnurr, P. P., Davis, K., Tobin, J. N., Cassells, A., & Gidengil, C. A. (2022). Protecting the mental and physical well-being of frontline health care workers during COVID-19: Study protocol of a cluster randomized controlled trial. *Contemp Clin Trials*, *117*, 106768. <https://doi.org/10.1016/j.cct.2022.106768>
5. Dumarkaite, A., Truskauskaite, I., Andersson, G., Jovarauskaite, L., Jovaisiene, I., Nomeikaite, A., & Kazlauskas, E. (2023). The efficacy of the internet-based stress recovery intervention FOREST for nurses amid the COVID-19 pandemic: A randomized controlled trial. Int J Nurs Stud, 138, 104408. https://doi.org/doi:10.1016/j.ijnurstu.2022.104408
6. Fiol-DeRoque, M. A., Serrano-Ripoll, M. J., Jimenez, R., Zamanillo-Campos, R., Yanez-Juan, A. M., Bennasar-Veny, M., Leiva, A., Gervilla, E., Garcia-Buades, M. E., Garcia-Toro, M., Alonso-Coello, P., Pastor-Moreno, G., Ruiz-Perez, I., Sitges, C., Garcia-Campayo, J., Llobera-Canaves, J., & Ricci-Cabello, I. (2021). A Mobile Phone-Based Intervention to Reduce Mental Health Problems in Health Care Workers During the COVID-19 Pandemic (PsyCovidApp): Randomized Controlled Trial. JMIR Mhealth Uhealth, 9(5), e27039. https://doi.org/10.2196/27039
7. Fogliato, E., Invernizzi, R., Maslovaric, G., Fernandez, I., Rigamonti, V., Lora, A., Frisone, E., & Pagani, M. (2022). Promoting Mental Health in Healthcare Workers in Hospitals Through Psychological Group Support With Eye Movement Desensitization and Reprocessing During COVID-19 Pandemic: An Observational Study. *Front Psychol*, *12*, 794178. <https://doi.org/10.3389/fpsyg.2021.794178>
8. Hannig, C., Lotzin, A., Milin, S., & Schäfer, I. (2021). Stress- und Traumaprävention für Beschäftigte im Gesundheitsbereich = Stress and trauma prevention for employees in the health sector. *Trauma & Gewalt*, *15*(3), 232-242. <https://doi.org/10.21706/tg-15-3-232>
9. Jovarauskaite, L., Dumarkaite, A., Truskauskaite-Kuneviciene, I., Jovaisiene, I., Andersson, G., & Kazlauskas, E. (2021). Internet-based stress recovery intervention FOREST for healthcare staff amid COVID-19 pandemic: study protocol for a randomized controlled trial. *Trials*, *22*(1), 559. <https://doi.org/10.1186/s13063-021-05512-1>
10. Kanellopoulos, D., Solomonov, N., Ritholtz, S., Wilkins, V., Goldman, R., Schier, M., Oberlin, L., Bueno-Castellano, C., Dargis, M., Cherestal, S., & Gunning, F. (2021). The CopeNYP program: A model for brief treatment of psychological distress among healthcare workers and hospital staff. *Gen Hosp Psychiatry*, *73*, 24-29. <https://doi.org/10.1016/j.genhosppsych.2021.09.002>
11. Lefevre, H., Stheneur, C., Cardin, C., Fourcade, L., Fourmaux, C., Tordjman, E., Touati, M., Voisard, F., Minassian, S., Chaste, P., Moro, M. R., & Lachal, J. (2021). The Bulle: Support and Prevention of Psychological Decompensation of Health Care Workers During the Trauma of the COVID-19 Epidemic. *J Pain Symptom Manage*, *61*(2), 416-422. <https://doi.org/10.1016/j.jpainsymman.2020.09.023>
12. Mellins, C. A., Mayer, L. E. S., Glasofer, D. R., Devlin, M. J., Albano, A. M., Nash, S. S., Engle, E., Cullen, C., Ng, W. Y. K., Allmann, A. E., Fitelson, E. M., Vieira, A., Remien, R. H., Malone, P., Wainberg, M. L., & Baptista-Neto, L. (2020). Supporting the well-being of health care providers during the COVID-19 pandemic: The CopeColumbia response. *Gen Hosp Psychiatry*, *67*, 62-69. <https://doi.org/10.1016/j.genhosppsych.2020.08.013>
13. Morina, N., Weilenmann, S., Dawson, K. S., Ernst, J., Zanitti, Z., von Känel, R., Schick, M., Spiller, T. R., & Bryant, R. A. (2021). RECHARGE - A Brief Psychological Intervention to Build Resilience in Health Care Workers During the COVID-19 Pandemic: Study Protocol for a Randomized Controlled Trial *Preprint* <https://doi.org/10.21203/rs.3.rs-212942/v1>
14. Sagaltici, E., Saydam, R. B., Cetinkaya, M., Şahin Ş, K., Küçük, S. H., & Müslümanoğlu, A. Y. (2022). Burnout and psychological symptoms in healthcare workers during the COVID-19 pandemic: Comparisons of different medical professions in a regional hospital in Turkey. *Work*, *72*(3), 1077-1085. <https://doi.org/10.3233/wor-210517>
15. Serrano-Ripoll, M. J., Ricci-Cabello, I., Jiménez, R., Zamanillo-Campos, R., Yañez-Juan, A. M., Bennasar-Veny, M., Sitges, C., Gervilla, E., Leiva, A., García-Campayo, J., García-Buades, M. E., García-Toro, M., Pastor-Moreno, G., Ruiz-Perez, I., Alonso-Coello, P., Llobera, J., & Fiol-deRoque, M. A. (2021). Effect of a mobile-based intervention on mental health in frontline healthcare workers against COVID-19: Protocol for a randomized controlled trial. *J Adv Nurs*, *77*(6), 2898-2907. <https://doi.org/10.1111/jan.14813>
16. Solomonov, N., Kanellopoulos, D., Grosenick, L., Wilkins, V., Goldman, R., Ritholtz, S., Falk, A., & Gunning, F. M. (2022). CopeNYP: A brief remote psychological intervention reduces health care workers’ depression and anxiety symptoms during COVID‐19 pandemic. *World Psychiatry*, *21*(1), 155-156. <https://doi.org/10.1002/wps.20946>
17. Sulaiman, A. H., Ahmad Sabki, Z., Jaafa, M. J., Francis, B., Razali, K. A., Juares Rizal, A., Mokhtar, N. H., Juhari, J. A., Zainal, S., & Ng, C. G. (2020). Development of a Remote Psychological First Aid Protocol for Healthcare Workers Following the COVID-19 Pandemic in a University Teaching Hospital, Malaysia. *Healthcare (Basel)*, *8*(3). <https://doi.org/10.3390/healthcare8030228> (Sulaiman et al., 2020)
18. Trottier, K., Monson, C. M., Kaysen, D., Wagner, A. C., Liebman, R. E., & Abbey, S. E. (2022). Initial findings on RESTORE for healthcare workers: an internet-delivered intervention for COVID-19-related mental health symptoms. *Transl Psychiatry*, *12*(1), 222. <https://doi.org/10.1038/s41398-022-01965-3>
19. Trottier, K., Monson, C. M., Kaysen, D., Wagner, A. C., Pun, C., & Abbey, S. E. (2021). Development of RESTORE: an online intervention to improve mental health symptoms associated with COVID-19-related traumatic and extreme stressors. *Eur J Psychotraumatol*, *12*(1), 1984049. <https://doi.org/doi:10.1080/20008198.2021.1984049>
20. Wang, L., Norman, I., Xiao, T., Li, Y., Li, X., & Leamy, M. (2022). Evaluating a Psychological First Aid Training Intervention (Preparing Me) to Support the Mental Health and Wellbeing of Chinese Healthcare Workers During Healthcare Emergencies: Protocol for a Randomized Controlled Feasibility Trial. *Front Psychiatry*, *12*, 809679. <https://doi.org/10.3389/fpsyt.2021.809679>
21. Weiner, L., Berna, F., Nourry, N., Severac, F., Vidailhet, P., & Mengin, A. C. (2020). Efficacy of an online cognitive behavioral therapy program developed for healthcare workers during the COVID-19 pandemic: the REduction of STress (REST) study protocol for a randomized controlled trial. *Trials*, *21*(1), 870. <https://doi.org/10.1186/s13063-020-04772-7>

# 14. Research update

The research update of the systematic literature search I in MEDLINE via PubMed and PsychINFO and CINAHL via EBSCO took place on 3^th^ May 2024. Therefore, the publication date of the search filter were adapted according to the period, between May 2023 and May 2024.

Table 15.Search filters (own representation based on Nordhausen and Hirt (2022))

| **Search filter** |  |
| --- | --- |
| **Publication Date** | May 2023 – May 2024 |
| **Language** | English, German |
| **Spezies** | Human |

In total, 31 articles could be included via PubMed and 20 via PsychINFO. After removing 8 duplicates, 7 were screened within the full-text-screening, based on not retrieved articles (n = 2).

The screening resulted in five articles for data extraction and -analysis from the two databases. The limited systematic literature search in CINAHL resulted in zero articles. Whereas in the backward citation tracking, two other articles could be identified. Finally, with the research update, a total of 28 articles could be included based on the eligibility criteria for data analysis. The flow-chart including the research update can be seen in Figure 1.

Figure 2: PRISMA-Flow Chart including the research update according to Page et al., 2020.

**Bibliographic information of included articles form the research update**

1. Ahmed Pihlgren, S., Johansson, L., Holmes, E. A., & Kanstrup, M. (2024). Exploring healthcare workers' experiences of a simple intervention to reduce their intrusive memories of psychological trauma: an interpretative phenomenological analysis. *Eur J Psychotraumatol*, *15*(1), 2328956. <https://doi.org/10.1080/20008066.2024.2328956>
2. Iyadurai L.; Highfield J.; Kanstrup M.; Markham, A. R. V. G. B. J. T. K. J. G. G. S. C. B. M. (2023). Reducing instrusive memories after trauma via an imagery-competing task intervention in COVID-19 intensive care staff: a randomised controlled trial *Transl Psychiatry*, *13*(290), 1-15. <https://doi.org/10:1038/s41398-023-02578-0>
3. Mediavilla, R., Felez-Nobrega, M., McGreevy, K. R., Monistrol-Mula, A., Bravo-Ortiz, M. F., Bayón, C., Giné-Vázquez, I., Villaescusa, R., Muñoz-Sanjosé, A., Aguilar-Ortiz, S., Figueiredo, N., Nicaise, P., Park, A. L., Petri-Romão, P., Purgato, M., Witteveen, A. B., Underhill, J., Barbui, C., Bryant, R., . . . Ayuso-Mateos, J. L. (2023). Effectiveness of a mental health stepped-care programme for healthcare workers with psychological distress in crisis settings: a multicentre randomised controlled trial. *BMJ Ment Health*, *26*(1). <https://doi.org/10.1136/bmjment-2023-300697>
4. Meredith, L. S., Ahluwalia, S., Chen, P. G., Dong, L., Farmer, C. M., Bouskill, K. E., Dalton, S., Qureshi, N., Blagg, T., Timmins, G., Schulson, L. B., Huilgol, S. S., Han, B., Williamson, S., Watson, P., Schnurr, P. P., Martineau, M., Davis, K., Cassells, A., & Tobin, J. N. (2024). Testing an Intervention to Improve Health Care Worker Well-Being During the COVID-19 Pandemic: A Cluster Randomized Clinical Trial. *JAMA Network Open*, *7*(4), e244192-e244192. <https://doi.org/10.1001/jamanetworkopen.2024.4192>
5. Morina, N., Weilenmann, S., Dawson, K. S., Möckli, U., Ernst, J., Zanitti, Z., von Känel, R., Schick, M., Spiller, T. R., & Bryant, R. A. (2023). Efficacy of a Brief Psychological Intervention to Reduce Distress in Healthcare Workers During the COVID-19 Pandemic: A Randomized Controlled Trial. *Psychological Trauma: Theory, Research, Practice & Policy*, *15*, S371-S383. <https://doi.org/10.1037/tra0001524>
6. Pratt, E. H., Hall, L., Jennings, C., Olsen, M. K., Jan, A., Parish, A., Porter, L. S., & Cox, C. E. (2023). Mobile Mindfulness for Psychological Distress and Burnout among Frontline COVID-19 Nurses: A Pilot Randomized Trial. *Ann Am Thorac Soc*, *20*(10), 1475-1482. <https://doi.org/10.1513/AnnalsATS.202301-025OC>
7. Singh, L., Kanstrup, M., Gamble, B., Geranmayeh, A., Goransson, K. E., Rudman, A., Dahl, O., Lindstrom, V., Horberg, A., Holmes, E. A., & Moulds, M. L. (2022). A first remotely-delivered guided brief intervention to reduce intrusive memories of psychological trauma for healthcare staff working during the ongoing COVID-19 pandemic: Study protocol for a randomised controlled trial. *Contemp Clin Trials Commun*, *26*, 100884. <https://doi.org/10.1016/j.conctc.2022.100884>

# References

Ahmed Pihlgren, S., Johansson, L., Holmes, E. A., & Kanstrup, M. (2024). Exploring healthcare workers' experiences of a simple intervention to reduce their intrusive memories of psychological trauma: an interpretative phenomenological analysis. *Eur J Psychotraumatol*, *15*(1), 2328956. <https://doi.org/10.1080/20008066.2024.2328956>

Bohart, S., Merete Møller, A., & Forsyth Herling, S. (2019). Do health care professionals worry about delirium? Relatives' experience of delirium in the intensive care unit: A qualitative interview study. *Intensive Crit Care Nurs*, *53*, 84-91. <https://doi.org/10.1016/j.iccn.2019.04.010>

Cooper, C., Booth, A., Britten, N., & Garside, R. (2017). A comparison of results of empirical studies of supplementary search techniques and recommendations in review methodology handbooks: a methodological review. *Syst Rev*, *6*(1), 234. <https://doi.org/10.1186/s13643-017-0625-1>

Couarraze, S., Delamarre, L., Marhar, F., Quach, B., Jiao, J., Avilés Dorlhiac, R., Saadaoui, F., Liu, A. S.-I., Dubuis, B., Antunes, S., Andant, N., Pereira, B., Ugbolue, U. C., Baker, J. S., Clinchamps, M., & Dutheil, F. (2021). The major worldwide stress of healthcare professionals during the first wave of the COVID-19 pandemic – the international COVISTRESS survey. *PLOS ONE*, *16*(10), e0257840. <https://doi.org/10.1371/journal.pone.0257840>

Evanoff, B. A., Strickland, J. R., Dale, A. M., Hayibor, L., Page, E., Duncan, J. G., Kannampallil, T., & Gray, D. L. (2020). Work-Related and Personal Factors Associated With Mental Well-Being During the COVID-19 Response: Survey of Health Care and Other Workers. *Journal of Medical Internet Research*, *22*(8), e21366. <https://doi.org/10.2196/21366>

Hooper, J. J., Saulsman, L., Hall, T., & Waters, F. (2021). Addressing the psychological impact of COVID-19 on healthcare workers: learning from a systematic review of early interventions for frontline responders. *BMJ Open*, *11*(5), e044134. <https://doi.org/10.1136/bmjopen-2020-044134>

Iyadurai L.; Highfield J.; Kanstrup M.; Markham, A. R. V. G. B. J. T. K. J. G. G. S. C. B. M. (2023). Reducing instrusive memories after trauma via an imagery-competing task intervention in COVID-19 intensive care staff: a randomised controlled trial *Transl Psychiatry*, *13*(290), 1-15. <https://doi.org/10:1038/s41398-023-02578-0>

Kanstrup, M., Singh, L., Goransson, K. E., Gamble, B., Taylor, R. S., Iyadurai, L., Moulds, M. L., & Holmes, E. A. (2021). A simple cognitive task intervention to prevent intrusive memories after trauma in patients in the Emergency Department: A randomized controlled trial terminated due to COVID-19. *BMC Res Notes*, *14*(1), 176. <https://doi.org/10.1186/s13104-021-05572-1>

Liang, L., Gao, T., Ren, H., Cao, R., Qin, Z., Hu, Y., Li, C., & Mei, S. (2020). Post-traumatic stress disorder and psychological distress in Chinese youths following the COVID-19 emergency. *J Health Psychol*, *25*(9), 1164-1175. <https://doi.org/10.1177/1359105320937057>

McGowan, J., Sampson, M., Salzwedel, D. M., Cogo, E., Foerster, V., & Lefebvre, C. (2016). PRESS Peer Review of Elektronic Search Strategies: 2015 Guideline Statement *Journal of Clinical Epidemiology*, *75*. <https://doi.org/10.1016/j.jclinepi.2016.01.021>

Mediavilla, R., Felez-Nobrega, M., McGreevy, K. R., Monistrol-Mula, A., Bravo-Ortiz, M. F., Bayón, C., Giné-Vázquez, I., Villaescusa, R., Muñoz-Sanjosé, A., Aguilar-Ortiz, S., Figueiredo, N., Nicaise, P., Park, A. L., Petri-Romão, P., Purgato, M., Witteveen, A. B., Underhill, J., Barbui, C., Bryant, R., . . . Ayuso-Mateos, J. L. (2023). Effectiveness of a mental health stepped-care programme for healthcare workers with psychological distress in crisis settings: a multicentre randomised controlled trial. *BMJ Ment Health*, *26*(1). <https://doi.org/10.1136/bmjment-2023-300697>

Meredith, L. S., Ahluwalia, S., Chen, P. G., Dong, L., Farmer, C. M., Bouskill, K. E., Dalton, S., Qureshi, N., Blagg, T., Timmins, G., Schulson, L. B., Huilgol, S. S., Han, B., Williamson, S., Watson, P., Schnurr, P. P., Martineau, M., Davis, K., Cassells, A., & Tobin, J. N. (2024). Testing an Intervention to Improve Health Care Worker Well-Being During the COVID-19 Pandemic: A Cluster Randomized Clinical Trial. *JAMA Network Open*, *7*(4), e244192-e244192. <https://doi.org/10.1001/jamanetworkopen.2024.4192>

Morina, N., Weilenmann, S., Dawson, K. S., Möckli, U., Ernst, J., Zanitti, Z., von Känel, R., Schick, M., Spiller, T. R., & Bryant, R. A. (2023). Efficacy of a Brief Psychological Intervention to Reduce Distress in Healthcare Workers During the COVID-19 Pandemic: A Randomized Controlled Trial. *Psychological Trauma: Theory, Research, Practice & Policy*, *15*, S371-S383. <https://doi.org/10.1037/tra0001524>

Nordhausen, T., & Hirt, J. (2022). RefHunter im neuen Webformat: Eine Plattform zur systematischen Literaturrecherche *GMS Medizin 22*(2). <https://doi.org/10.3205/mbi000549>

Ouzzani, M., Hammady, H., Fedorowicz, Z., & Elmagarmid, A. (2016). Rayyan—a web and mobile app for systematic reviews. *Systematic Reviews*, *5*(1). <https://doi.org/10.1186/s13643-016-0384-4>

Peters, M. D. J., Marnie, C., Tricco, A. C., Pollock, D., Munn, Z., Alexander, L., McInerney, P., Godfrey, C. M., & Khalil, H. (2020). Updated methodological guidance for the conduct of scoping reviews. *JBI Evid Synth*, *18*(10), 2119-2126. <https://doi.org/10.11124/JBIES-20-00167>

Pratt, E. H., Hall, L., Jennings, C., Olsen, M. K., Jan, A., Parish, A., Porter, L. S., & Cox, C. E. (2023). Mobile Mindfulness for Psychological Distress and Burnout among Frontline COVID-19 Nurses: A Pilot Randomized Trial. *Ann Am Thorac Soc*, *20*(10), 1475-1482. <https://doi.org/10.1513/AnnalsATS.202301-025OC>

Saragih, I. D., Tonapa, S. I., Saragih, I. S., Advani, S., Batubara, S. O., Suarilah, I., & Lin, C. J. (2021). Global prevalence of mental health problems among healthcare workers during the Covid-19 pandemic: A systematic review and meta-analysis. *Int J Nurs Stud*, *121*, 104002. <https://doi.org/10.1016/j.ijnurstu.2021.104002>

Singh, L., Kanstrup, M., Gamble, B., Geranmayeh, A., Goransson, K. E., Rudman, A., Dahl, O., Lindstrom, V., Horberg, A., Holmes, E. A., & Moulds, M. L. (2022). A first remotely-delivered guided brief intervention to reduce intrusive memories of psychological trauma for healthcare staff working during the ongoing COVID-19 pandemic: Study protocol for a randomised controlled trial. *Contemp Clin Trials Commun*, *26*, 100884. <https://doi.org/10.1016/j.conctc.2022.100884>

Stuijfzand, S., Deforges, C., Sandoz, V., Sajin, C.-T., Jaques, C., Elmers, J., & Horsch, A. (2020). Psychological impact of an epidemic/pandemic on the mental health of healthcare professionals: a rapid review. *BMC Public Health*, *20*(1). <https://doi.org/10.1186/s12889-020-09322-z>

Taylor-Desir, M. (2022). *What is Posttraumatic Stress Disorder (PTSD)* Amercian Psychiatric Association <https://www.psychiatry.org/patients-families/ptsd/what-is-ptsd>

WHO. (2019). *International Statistical Classification of Diseases and Related Health Problems 10th Revision (ICD-10)-WHO Version for 2019-covid-expanded*

*Chapter V Mental and behavioral disorders (F00-F99)* World Health Organization <https://icd.who.int/browse10/2019/en#/F43.2>
