# Supplementary material for: Factors influencing the implementation of interventions for symptoms of posttraumatic stress disorder among hospital-based nurses and physicians during the COVID-19 pandemic: a scoping review
Source: BMC Health Serv Res. 2025 Jul 2;25:885. doi: 10.1186/s12913-025-13005-z (PMC12225526; doi:10.1186/s12913-025-13005-z)
Supplement: Supplementary file 3 — Additional File 3. Research protocol_Influencing factors. [file 12913_2025_13005_MOESM3_ESM.docx]

***Additional File 3: Research protocol II***

**Research protocol**

**Analyzing factors that influence the implementation of interventions treating symptoms of post-traumatic stress disorder among hospital-based nurses and physicians during the COVID-19 pandemic**

Content

[List of tables III](#_Toc173658806)

[List of figures III](#_Toc173658807)

[1 Research question 4](#_Toc173658808)

[2 Inclusion and exclusion criteria 4](#_Toc173658809)

[3 Search strategies 6](#_Toc173658810)

[4 Identification of synonym search terms 6](#_Toc173658811)

[5 Identification of keywords 8](#_Toc173658812)

[6 Search string development 11](#_Toc173658813)

[7 Review search strings 27](#_Toc173658814)

[8 Conducting and documentation of the search 31](#_Toc173658815)

[9 Supplementary search options 49](#_Toc173658816)

[10 Title-Abstract- and full-text-screening 59](#_Toc173658817)

[12 Supplement I Flowchart 60](#_Toc173658818)

[13 Supplement II Bibliographic information of the articles included for the data synthesis 61](#_Toc173658819)

[14 Research update 64](#_Toc173658820)

[*15* References 68](#_Toc173658821)

# List of tables

[Table 1: Inclusion and exclusion criteria (own visualization) 4](#_Toc173658250)

[Table 2: Synonym search terms (own visualization based on Nordhausen and Hirt (2022)) 6](#_Toc173658251)

[Table 3: Identified key words (own visualization based on Nordhausen and Hirt (2022)) 8](#_Toc173658252)

[Table 4: Search string for the database MEDLINE via PubMed (own visualization based on Nordhausen and Hirt (2022)) 11](#_Toc173658253)

[Table 5: Search filters (own visualization based on Nordhausen and Hirt (2022)) 17](#_Toc173658254)

[Table 6: Rehearsal with the generated search string performed in MEDLINE via PubMed based on Nordhausen and Hirt (2022)) 17](#_Toc173658255)

[Table 7: Search string syntax for MEDLINE via PubMed and Psych INFO via EBSCO (own visualization based on Nordhausen and Hirt (2022)) 21](#_Toc173658256)

[Table 8: Documentation of the search (own visualization based on Nordhausen and Hirt (2022)) 31](#_Toc173658257)

[Table 9: Documentation of the search (own visualization based on Nordhausen and Hirt (2022)) 32](#_Toc173658258)

[Table 10: Documentation of the search in PsychINFO via EBSCO (own visualization based on Nordhausen and Hirt (2022)) 39](#_Toc173658259)

[Table 11: Documentation of the search in CINAHL via EBSCO (own visualization based on Nordhausen and Hirt (2022)) 50](#_Toc173658260)

[Table 15. Search filters (own representation based on Nordhausen and Hirt (2022)) 64](#_Toc173658261)

# List of figures

[Figure 1: PRISMA-Flowchart of the systematic literature searches in databases according to Page et al., 2020 60](#_Toc173658262)

[Figure 2: PRISMA-Flow Chart including the research update according to Page et al., 2020 65](#_Toc173658263)

# 1 Research question

The aim of this systematic literature research is to identify studies, which describe the implementation of interventions treating PTSD in nurses and physicians working in an acute hospital setting.

The central research question guiding this systematic literature search is:

*What are the barriers/facilitators in the implementation of PTSD-related interventions for nurses and physicians working in an acute hospital setting during the COVID-19 pandemic?*

# 2 Inclusion and exclusion criteria

Based on the **PCC**-elements (**P**opulation, **C**oncept of interest, **C**ontext) the in- and exclusion criteria are defined in table 1 below:

Table 1: Inclusion and exclusion criteria (own visualization)

| Criteria | Definition (inclusion) | Definition (exclusion) |
| --- | --- | --- |
| Population | - Nurses and physicians affect by symptoms of post-traumatic stress disorder (PTSD) as target population for the interventions - Symptoms of PTSD^1^:   - **Intrusion** (e.g., intrusive thoughts, involuntary memories, distressing dreams, flashbacks)   - **Avoidance** (e.g., avoiding people, places, activities, objects and situations)   - **Alterations in cognition and mood** (e.g., feeling detached or estranged from others, unable to experience positive emotions)   - **Alterations in arousal and reactivity** (e.g., insomnia)   - Distress (psychological)   - Anhedonia   - Anxiety   - Depression   - Suicidal ideations   - Acute stress (disorder) | - Other professions (e.g., Community Health Nurses, physiotherapist, respiratory therapist) |
| Concept of interest | - Implementation of interventions treating symptoms of PTSD | - Non-PTSD related interventions |
| Context | - Studies in acute somatic hospitals during the COVID-19 pandemic period | - Specialized clinics such as mental/ psychiatric hospital |
| Types of evidence sources | - Any kind of study that describes or evaluate interventions addressing symptoms of PTSD: - Evaluation studies - Implementation studies - Study protocol - Feasibility studies - Qualitive studies - Concept article | - Reviews |
| Other | - Languages: German and English - Year: 2020- 2023 | - Not published before 2020 |

^1^ (Taylor-Desir, 2022; WHO, 2019)

Rationale for in- and exclusion criteria:

*Types of participants*

The focus is on health-care workers (HCWs) specifically on physicians and nurses who were working/ work during the COVID-19 pandemic in period from May 2020 until December 2022. Couarraze et al. (2021) stated in an international survey that the levels of stress from healthcare professionals was 25.8% higher during the pandemic than the general population.

Saragih et al. (2021) ascertain that during the pandemic globally the prevalence of PTSD in physicians and nurses is as a result from a pooled analysis 49%. The researchers included 38 studies in the year 2020, which provided a comprehensive overview of the effects of the pandemic, globally. Across all studies, a total of 53,784 participants were included. Of it, 27,9% were doctors and 43,7% nurses.

*Concept*

Implementing interventions into routine practice faces pandemic-specific challenges. For instance, decision makers came to radical decisions, a rapid implementation without an appropriate implementation approach, or changes in interventions as external modifying conditions (e.g.: policy orders) (Wensing et al., 2020).

By means of identifying barriers and facilitators at the beginning of the implementation process, interventions are more likely to be used in practice in a sustainable way. (Damschroder et al., 2009; Damschroder et al., 2022; Stetler et al., 2006).

*Context*

Context of interest is the COVID-19 pandemic because this and any kind of disease outbreak could increase risk of mental health problems among direct healthcare workers in short or long term (Stuijfzand et al., 2020). Only acute somatic hospital-based physicians and nurses are the focus of this study, because they have been caring to a larger extent for patients infected with COVID-19. This kind of environment is associated with a higher prevalence of stress, anxiety and burnout among staff (Evanoff et al., 2020).

# 3 Search strategies

Based on the PCC-elements (Peters et al., 2022), MEDLINE (via PubMed) is chosen because of the wide range of topics in the field of health and the use of different research methods. In addition, PsychINFO (via EBSCO) is specialized in psychiatric/ mental health issues, which is necessary for getting relevant literature to answer the research question. A preceding search was conducted to identify existing or developed evidence-based interventions based on the phenomenon. Components of previously identified interventions will be included in the planned systematic literature search.

Initially, a limited search will be conducted in two databases to identify synonym search terms and key words for each. Second, search strings for each database will be developed to conduct systematic literature research. Third, the search string for MEDLINE (via PubMed), will be verify using the Peer Review of Electronic Search Strategies (PRESS) (McGowan et al., 2016). Fourth, search string for PsychINFO (via EBSCO) will be developed. Finally, further search possibilities as a supplemental limited search in CINAHL (via EBSCO), citation tracking will be conducted (Peters et al., 2020).

# 4 Identification of synonym search terms

Table 2: Synonym search terms (own visualization based on (Nordhausen & Hirt, 2022)).

| **Criteria/ search component** | **Search terms** | |
| --- | --- | --- |
| **Population** | Physician/Physicians  Medical Doctor  Nurse/Nurses  Nursing staff  Medical staff  Professional Caregivers  Registered nurse | Nurse practitioner  Advanced practice nurse  health care workers  health workers  health-care workers  health care professionals  frontline healthcare workers |
| **Concept of interest** | Intervention  post-traumatic stress disorder  PTSD  Acute stress disorder  Psychological Distress | |
|  | Implementation  Implementation Science  Quality Improvement  Diffusion  Diffusion of innovation  Knowledge translation  Knowledge exchange  Knowledge circulation  Facilitators  Barriers  Process evaluation  Formative evaluation  Summative evaluation  Qualitative evaluation  Resilience  Peer Support  Anticipate-Plan-Deter  Social Support  Self-Care  Manage Emotions  Psychoeducation  Cognitive Behavioral Therapy-Program  Coping strategies  Mindfulness  Psychological hotline  Stress First Aid  Eye Movement Desensitization and Preprocessing Therapy  Adaptive Information Processing model  Bilateral self-stimulation  Stress recovery  Life-work balance  Behavioral activation  Emotion regulation skills  Relaxation  Physical Activity  Problem Management Plus  Self-stabilization exercises  Psychological First Aid | Cognitive Processing Therapy  Sustainability  Practicability  Feasibility  Fidelity  Maintenance  Adoption  Dissemination  Promotion  Adaptation |
| **Context** | hospital  acute hospital  clinic  acute setting  hospital setting | COVID-19  COVID-19 pandemic |

# 5 Identification of keywords

Table 3: Identified key words (own visualization based on Nordhausen and Hirt (2022))

| **Search terms** | **Search component** | **keyword (MEDLINE via PubMed)** | **keyword (Psych INFO via EBSCO)** |
| --- | --- | --- | --- |
| Physician  Physicians  Doctor  Physician Assistant  Nurse  Nurses  Nursing staff  Caregivers  Registered nurse  Nurse practitioner  Advanced practice nurse  health care workers  health workers  health-care workers  health care professionals  frontline healthcare workers | **Population** | Nurse Clinicians  Nurse Specialists  Physician Assistants  Nurse Specialists  Nursing Staff, Hospital  Health Personnel | Physicians  Medical Personnel  Clinicians  Nurses  Caregivers  Professional Personnel  Medical Personnel  Frontline Employees  Health Personnel |
| Intervention  post-traumatic stress disorder  PTSD  Acute stress disorder  Psychological Distress  Resilience  Peer Support  Anticipate-Plan-Deter  Social Support  Self-Care  Manage Emotions  Psychoeducation  Cognitive Behavioral Therapy  Coping strategy  Mindfulness  Psychological hotline  Eye Movement Desensitization and Preprocessing Therapy  Adaptive Information Processing model  Bilateral self-stimulation  Stress recovery  Life-work balance  Behavioral activation  Emotion regulation  Relaxation  Physical Activity  Self-stabilization  Psychological First Aid  Cognitive Processing Therapy  Implementation  Implementation Science  Quality Improvement  Diffusion  Diffusion of innovation  Knowledge translation  Knowledge exchange  Knowledge circulation  Facilitators  Barriers  Process evaluation  Formative evaluation  Summative evaluation  Qualitative evaluation  Sustainability  Practicability  Feasibility  Fidelity  Maintenance  Dissemination  Promotion | **Concept of interest** | Stress Disorders, Post-Traumatic Psychological Distress  Stress Disorders, Traumatic, Acute  Resilience, Psychological  Social Support  Self-Care  Cognitive Behavioral Therapy  Adaptation, Psychological  Mindfulness  Eye Movement Desensitization Reprocessing  Work-Life Balance  Emotional Regulation  Relaxation  Exercise  Psychological First Aid  Health Plan Implementation  Implementation Science  Quality improvement  Diffusion of Innovation | Intervention  Posttraumatic Stress Disorder  Postraumatic Stress  Trauma  Acute Stress Disorder  Stress and Trauma related disorders  Caregiver burden  Resilience (Psychological)  Peers  Social Support  Self-Care  Psychoeducation  Coping Style  Mindfulness  Mindfulness-Based Interventions  Eye Movement Desensitization Therapy  Relaxation  Physical Activity  Psychological First Aid  Cognitive Processing Therapy  Cognitive Behavior Therapy  Sustainability |
| hospital  hospitals  acute hospital  clinic  acute setting  hospital setting  COVID-19  COVID-19 pandemic | **Context** | Hospitals  Subacute care  COVID-19 | Hospitals  Clinics  Treatment Facilities  COVID-19 |

Rationale for keywords in MEDLINE via PubMed

*Concept*

*Adaptation, Psychological* was identified as a keyword for “Coping-strategy” with the following description: “A **state of harmony** between **internal needs and external demands** and the processes used in achieving this condition. (From APA Thesaurus of Psychological Index Terms, 8th ed).”

# 6 Search string development

According to the recommendation of Nordhausen and Hirt (2022) first the search string for the database MEDLINE via PubMed will be developed. In the subsequent step, this will be tested in a trial run following the recommendation so that an adjustment can be made and thus the effort for the PsychINFO via EBSCO database is minimized.

Table 4: Search string for the database MEDLINE via PubMed (own visualization based on Nordhausen and Hirt (2022))

| **Search components** | **Searchstring MEDLINE via PubMed** | **Searchstring PsychINFO via EBSCO** |
| --- | --- | --- |
| **Population** | Physician* [TIAB]  OR  Doctor [TIAB]  OR  “Physician Assistant” [TIAB]  OR  Nurs* [TIAB]  OR  “Nursing staff” [TIAB]  OR  Caregiver* [TIAB]  OR  “Registered nurse” [TIAB]  OR  “Nurse practitioner” [TIAB]  OR  “Advanced practice nurse” [TIAB]  OR  “health care workers” [TIAB]  OR  “health workers” [TIAB]  OR  “health-care workers” [TIAB]  OR  “health care professionals” [TIAB]  OR  “frontline healthcare workers” [TIAB]  OR  “Nurse Clinicians” [MH]  OR  “Nurse Specialists” [MH]  OR  “Physician Assistants” [MH]  OR  “Nurse Specialists” [MH]  OR  “Nursing Staff, Hospital” [MH]  OR  “Health Personnel” [MH] |  |
|  | **AND** | **AND** |
| **Concept of interest** | Intervention* [TIAB] |  |
|  | **AND** | **AND** |
|  | “post-traumatic stress disorder” [TIAB]  OR  PTSD [TIAB]  OR  “Acute Stress Disorder” [TIAB]  OR  “Psychological Distress” [TIAB]  OR  “Stress Disorders, Post-Traumatic” [MH]  OR  “Psychological Distress” [MH]  OR  “Stress Disorders, Traumatic, Acute” [MH] |  |
|  | **AND** | **AND** |
|  | Resilience [TIAB]  OR  “Peer Support” [TIAB]  OR  “Anticipate-Plan-Deter” [TIAB]  OR  “Social Support” [TIAB]  OR  “Self-Care” [TIAB]  OR  “Manage Emotions” [TIAB]  OR  Psychoeducation [TIAB]  OR  “Cognitive Behavioral Therapy” [TIAB]  OR  “Coping strategy” [TIAB]  OR  Mindfulness [TIAB]  OR  “Psychological hotline” [TIAB]  OR  “Eye Movement Desensitization and Preprocessing Therapy” [TIAB]  OR  “Adaptive Information Processing model” [TIAB]  OR  “Bilateral self-stimulation” [TIAB]  OR  “Stress recovery” [TIAB]  OR  “Life-work balance” [TIAB]  OR  “Behavioral activation” [TIAB]  OR  “Emotion regulation” [TIAB]  OR  Relaxation [TIAB]  OR  “Physical Activity” [TIAB]  OR  “Self-stabilization” [TIAB]  OR  “Psychological First Aid” [TIAB]  OR  “Cognitive Processing Therapy” [TIAB]  OR  “Resilience, Psychological” [MH]  OR  “Social Support” [MH]  OR  “Self-Care” [MH]  OR  “Cognitive Behavioral Therapy” [MH]  OR  “Adaptation, Psychological” [MH]  OR  Mindfulness [MH]  OR  “Eye Movement Desensitization Reprocessing” [MH]  OR  “Work-Life Balance” [MH]  OR  “Emotional Regulation” [MH]  OR  Relaxation [MH]  OR  Exercise [MH]  OR  “Psychological First Aid” [MH] |  |
|  | **AND** |  |
|  | Implement* [TIAB]  OR  “Implementation Science” [TIAB]  OR  “Quality Improvement*” [TIAB]  OR  Diffusion [TIAB]  OR  “Diffusion of innovation” [TIAB]  OR  “Knowledge translation” [TIAB]  OR  “Knowledge exchange” [TIAB]  OR  “Knowledge circulation” [TIAB]  OR  Facilitators [TIAB]  OR  Barriers [TIAB]  OR  “Process evaluation*” [TIAB]  OR  “Formative evaluation*” [TIAB]  OR  “Summative evaluation*” [TIAB]  OR  “Qualitative evaluation*” [TIAB]  OR  Sustainability [TIAB]  OR  Practicability [TIAB]  OR  Feasibility [TIAB]  OR  Fidelity [TIAB]  OR  Maintenance [TIAB]  OR  Disseminat* [TIAB]  OR  Promot* [TIAB]  OR  “Health Plan Implementation” [MH]  OR  “Implementation Science” [MH]  OR  “Quality improvement” [MH]  OR  “Diffusion of Innovation” [MH] |  |
|  | **AND** | **AND** |
| **Context** | Hospital* [TIAB]  OR  “acute hospital” [TIAB]  OR  clinic* [TIAB]  OR  “acute setting” [TIAB]  OR  “hospital setting” [TIAB]  OR  Hospitals [MH]  OR  “subacute care” [MH] |  |
|  | **AND** | **AND** |
|  | COVID-19 [TIAB]  OR  “COVID-19 pandemic” [TIAB]  OR  COVID-19 [MH] |  |

The rehearsal with the generated search string was performed on 11^th^May 2023 in MEDLINE via PubMed (the search string was stored online).

The following search filters were used:

Table 5: Search filters (own visualization based on Nordhausen and Hirt (2022))

| **Search filters** |  |
| --- | --- |
| **Publication Date** | 2020-2023 |
| **Language** | German, English |
| **Spezies** | Humans |

Table 6: Rehearsal with the generated search string performed in MEDLINE via PubMed based on Nordhausen and Hirt (2022))

| Search number | Input | Number of hits |
| --- | --- | --- |
| 1 | Physician* [TIAB] | 456,586 |
| 2 | Doctor [TIAB] | 67,202 |
| 3 | "Physician Assistant" [TIAB] | 1,969 |
| 4 | Nurs* [TIAB] | 529,425 |
| 5 | "Nursing staff" [TIAB] | 14,588 |
| 6 | Caregiver* [TIAB] | 90,233 |
| 7 | "Registered nurse" [TIAB] | 4,310 |
| 8 | "Nurse practitioner" [TIAB] | 7,021 |
| 9 | "Advanced practice nurse" [TIAB] | 1,126 |
| 10 | "health care workers" [TIAB] | 17,229 |
| 11 | "health workers" [TIAB] | 21,773 |
| 12 | "health-care workers" [TIAB] | 17,229 |
| 13 | "health care professionals" [TIAB] | 28,263 |
| 14 | “frontline healthcare workers” [TIAB] | 512 |
| 15 | "Nurse Clinicians" [MH] | 8,510 |
| 16 | "Physician Assistants" [MH] | 6,405 |
| 17 | "Nursing Staff, Hospital" [MH] | 47,902 |
| 18 | "Health Personnel" [MH] | 608,086 |
| 19 | #1 OR #2 OR #3 OR #4 OR #5 OR #6 OR #7 OR #8 OR #9 OR #10 OR #11 OR #12 OR #13 OR #14 OR #15 OR #16 OR #17 OR #18 OR #18 | 1,453,521 |

| Search number | Input | Number of hits |
| --- | --- | --- |
| 20 | Intervention* [TIAB] | 1,311,222 |
| 21 | "post-traumatic stress disorder" [TIAB] | 16,071 |
| 22 | PTSD [TIAB] | 33,106 |
| 23 | "Acute Stress Disorder" [TIAB] | 780 |
| 24 | “Psychological Distress” [TIAB] | 27,675 |
| 25 | “Stress Disorders, Post-Traumatic” [MH] | 40,842 |
| 26 | “Psychological Distress” [MH] | 6,659 |
| 27 | “Stress Disorders, Traumatic, Acute” [MH] | 536 |
| 28 | #20 OR #21 OR #22 OR #23 OR #24 OR #25 OR #26 OR #27 | 84,316 |
| 29 | **Resilience [TIAB]** | 44,849 |
| 30 | **"Peer Support" [TIAB]** | 6,998 |
| 31 | **"Anticipate-Plan-Deter" [TIAB]** | 1 |
| 32 | **"Social Support" [TIAB]** | 52,876 |
| 33 | **"Self-Care" [TIAB]** | 24,458 |
| 34 | **"Manage Emotions" [TIAB] - Schema: all** | 0 |
| 35 | **Psychoeducation [TIAB]** | 5,025 |
| 36 | **"Cognitive Behavioral Therapy" [TIAB]** | 13,150 |
| 37 | **"Coping strategy" [TIAB]** | 2,963 |
| 38 | **Mindfulness [TIAB]** | 12,532 |
| 39 | **"Psychological hotline" [TIAB]** | 5 |
| 40 | **"Eye Movement Desensitization and Preprocessing Therapy" [TIAB] - Schema: all** | 0 |
| 41 | **"Adaptive Information Processing model" [TIAB]** | 6 |
| 42 | **"Bilateral self-stimulation" [TIAB] - Schema: all** | 0 |
| 43 | **"Stress recovery" [TIAB]** | 665 |
| 44 | **"Life-work balance" [TIAB]** | 25 |
| 45 | **"Behavioral activation" [TIAB]** | 1,811 |
| 46 | **"Emotion regulation" [TIAB]** | 11,626 |
| 47 | **Relaxation [TIAB]** | 133,956 |
| 48 | **"Physical Activity" [TIAB]** | 146,176 |
| 49 | **"Self-stabilization" [TIAB]** | 103 |
| 50 | **"Psychological First Aid" [TIAB]** | 319 |
| 51 | **"Cognitive Processing Therapy" [TIAB]** | 513 |
| 52 | **"Resilience, Psychological" [MH]** | 8,288 |
| 53 | **"Social Support" [MH]** | 79,510 |
| 54 | **"Self-Care" [MH]** | 61,913 |
| 55 | **"Cognitive Behavioral Therapy" [MH]** | 36,174 |
| 56 | **"Adaptation, Psychological" [MH]** | 139,252 |
| 57 | **Mindfulness [MH]** | 6,327 |
| 58 | **"Eye Movement Desensitization Reprocessing" [MH]** | 276 |
| 59 | **"Emotional Regulation" [MH]** | 2,233 |
| 60 | **Relaxation [MH]** | 21,794 |
| 61 | **Exercise [MH]** | 244,275 |
| 62 | **"Psychological First Aid" [MH]** | 31 |
| 63 | **#29 OR #30 OR #31 OR #32 OR #33 OR #34 OR #35 OR #36 OR #37 OR #38 OR #39 OR #40 OR #41 OR #42 OR #43 OR #44 OR #45 OR #46 OR #47 OR #48 OR #49 OR #50 OR #51 OR #52 OR #53 OR #54 OR #55 OR #56 OR #57 OR #58 OR #59 OR #60 OR #61 OR #62** | 862,771 |
| 64 | **Implement* [TIAB]** | 683,068 |
| 65 | **"Implementation Science" [TIAB]** | 5,223 |
| 66 | **"Quality Improvement*" [TIAB]** | 53,603 |
| 67 | **Diffusion [TIAB]** | 242,221 |
| 68 | **"Diffusion of innovation" [TIAB]** | 684 |
| 69 | **"Knowledge translation" [TIAB]** | 4,365 |
| 70 | **"Knowledge exchange" [TIAB]** | 896 |
| 71 | **"Knowledge circulation" [TIAB]** | 19 |
| 72 | **Facilitators [TIAB]** | 25,206 |
| 73 | **Barriers [TIAB]** | 189,992 |
| 74 | **"Process evaluation*" [TIAB]** | 5,534 |
| 75 | **"Formative evaluation*" [TIAB]** | 1,377 |
| 76 | **"Summative evaluation*" [TIAB]** | 436 |
| 77 | **"Qualitative evaluation*" [TIAB]** | 5,348 |
| 78 | **Sustainability [TIAB]** | 42,475 |
| 79 | **Practicability [TIAB]** | 5,719 |
| 80 | **Feasibility [TIAB]** | 239,457 |
| 81 | **Fidelity [TIAB]** | 37,141 |
| 82 | **Maintenance [TIAB]** | 321,797 |
| 83 | **Disseminat* [TIAB]** | 160,976 |
| 84 | **Promot* [TIAB]** | 1,315,047 |
| 85 | **"Health Plan Implementation" [MH]** | 6,629 |
| 86 | **"Implementation Science" [MH]** | 1,252 |
| 87 | **"Quality improvement" [MH]** | 33,358 |
| 88 | **"Diffusion of Innovation" [MH]** | 21,473 |
| 89 | **#64 OR #65 OR #66 OR #67 OR #68 OR #69 OR #70 OR #71 OR #72 OR #73 OR #74 OR #75 OR #76 OR #77 OR #78 OR #79 OR #80 OR #81 OR #82 OR #83 OR #84 OR #85 OR #86 OR #87 OR #88** | 3,044,346 |
| 90 | #20 AND #28 AND #63 AND #89 | 1,973 |
| 91 | Hospital* [TIAB] | 1,612,584 |
| 92 | "acute hospital" [TIAB] | 4,324 |
| 93 | clinic* [TIAB] | 5,183,831 |
| 94 | "acute setting" [TIAB] | 2,704 |
| 95 | "hospital setting" [TIAB] | 14,229 |
| 96 | Hospitals [MH] | 315,577 |
| 97 | “Subacute care” [MH] | 1,414 |
| 98 | #91 OR #92 OR #93 OR #94 OR #95 OR #96 OR #97 | 6,292,050 |
| 99 | COVID-19 [TIAB] | 312,446 |
| 100 | "COVID-19 pandemic" [TIAB] | 118,085 |
| 101 | COVID-19 [MH] | 221,666 |
| 102 | #99 OR #100 OR #101 | 340,757 |
| 103 | #19 AND #90 AND #98 AND #102 | 29 |
| *Filter* | *Humans, English, German* | **20** |

**Results**

First, the search component *“intervention”* was removed from the search syntax because the search syntax already included components of interventions.

Secondly, the following search terms were excluded after a simple Title-Abstract-Screening:

- Mange emotions
- Bilateral self-stimulation
- Life-work balance
- Eye Movement and Desensitization and Preprocessing Therapy
- Knowledge circulation
- Psychological hotline
- Self-stabilization

Instead of the full name and/ or both e.g., Eye Movement and Desensitization and Preprocessing Therapy, the abbreviation was used (EMDR or PFA).

**Adjustment of the search string syntax**

Table 7: Search string syntax for MEDLINE via PubMed and Psych INFO via EBSCO (own visualization based on Nordhausen and Hirt (2022))

| **Search component** | **Search string MEDLINE via PubMed** | **Search string PsychINFO via EBSCO** |
| --- | --- | --- |
| **Population** | Physician* [TIAB]  OR  Doctor [TIAB]  OR  “Doctor of medicine” [TIAB]  OR  “Physician Assistant” [TIAB]  OR  Nurs* [TIAB]  OR  “Nursing staff” [TIAB]  OR  Caregiver* [TIAB]  OR  “Registered nurse” [TIAB]  OR  “Nurse practitioner” [TIAB]  OR  “Advanced practice nurse” [TIAB]  OR  “health care workers” [TIAB]  OR  “health workers” [TIAB]  OR  “health-care workers” [TIAB]  OR  “health care professionals” [TIAB]  OR  “frontline healthcare workers” [TIAB]  OR  Physicians [MH]  OR  medical staff, hospital [MH]  OR  Nurses [MH]  OR  Nurse Practitioner [MH]  OR  Nursing Staff [MH]  OR  Caregivers [MH]  OR  Nurse Clinicians [MH]  OR  Nurse Specialists [MH]  OR  Physician Assistants [MH]  OR  Nurse Specialists [MH]  OR  Nursing Staff, Hospital [MH]  OR  Health Personnel [MH] | (TI Physician* OR AB Physician* OR TI Doctor OR AB Doctor OR TI “Doctor of medicine” OR AB “Doctor of medicine” OR TI “Physician Assistant” OR AB “Physician Assistant” OR TI Nurs* OR AB Nurs* OR TI “Nursing staff” OR AB “Nursing staff” OR TI Caregiver* OR AB Caregiver* OR TI “Registered nurse” OR AB “Registered nurse” OR TI “Nurse practitioner” OR AB “Nurse practitioner” OR TI “Advanced practice nurse” OR AB “Advanced practice nurse” OR TI “health care workers” OR AB “health care workers” OR TI “health workers” OR AB “health workers” OR TI “health-care workers” OR AB “health-care workers” OR TI “health care professionals” OR AB “health care professionals” OR TI “frontline healthcare workers” OR AB “frontline healthcare workers” OR DE Health Personnel OR DE Physicians OR DE Medical Personnel OR DE Clinicians OR DE Nurses OR DE Caregivers OR DE Professional Personnel OR DE Medical Personnel OR DE Frontline Employees) |
|  | **AND** | **AND** |
| **Concept of interest** | “post-traumatic stress disorder” [TIAB]  OR  PTSD [TIAB]  OR  “Acute Stress Disorder” [TIAB]  OR  “Psychological Distress” [TIAB]  OR  “Stress Disorders, Post-Traumatic” [MH]  OR  “Psychological Distress” [MH]  OR  “Stress Disorders, Traumatic, Acute” [MH] | (TI “post-traumatic stress disorder” OR AB “post-traumatic stress disorder” OR TI PTSD OR AB PTSD OR TI “Acute Stress Disorder” OR AB “Acute Stress Disorder” OR TI “Psychological Distress” OR AB “Psychological Distress” OR DE “Posttraumatic Stress Disorder” OR DE “Posttraumatic Stress” OR DE Trauma OR DE “Acute Stress Disorder” OR DE “Stress and Trauma related disorders” OR DE “Caregiver burden”) |
|  | **AND** |  |
|  | Resilience [TIAB]  OR  “Peer Support” [TIAB]  OR  “Anticipate-Plan-Deter” [TIAB]  OR  “Social Support” [TIAB]  OR  “Self-Care” [TIAB]  OR  Psychoeducation [TIAB]  OR  “Cognitive Behavioral Therapy” [TIAB]  OR  “Coping strategy” [TIAB]  OR  Mindfulness [TIAB]  OR  EMDR [TIAB]  OR  “Stress recovery” [TIAB]  OR  “Behavioral activation” [TIAB]  OR  “Emotion regulation” [TIAB]  OR  Relaxation [TIAB]  OR  “Physical Activity” [TIAB]  OR  “Psychological First Aid” [TIAB]  OR  “Cognitive Processing Therapy” [TIAB]  OR  PFA [TIAB]  OR  “Resilience, Psychological” [MH]  OR  “Social Support” [MH]  OR  “Self-Care” [MH]  OR  “Cognitive Behavioral Therapy” [MH]  OR  “Adaptation, Psychological” [MH]  OR  Mindfulness [MH]  OR  “Eye Movement Desensitization Reprocessing” [MH]  OR  “Work-Life Balance” [MH]  OR  “Emotional Regulation” [MH]  OR  Relaxation [MH]  OR  Exercise [MH]  OR  “Psychological First Aid” [MH] | (TI Resilience OR AB Resilience OR TI “Peer Support” OR AB “Peer Support” OR TI “Anticipate-Plan-Deter” OR AB “Anticipate-Plan-Deter” OR TI “Social Support” OR AB “Social Support” OR TI “Self-Care” OR AB “Self-Care OR TI Psychoeducation OR AB Psychoeducation OR TI “Cognitive Behavioral Therapy” OR AB “Cognitive Behavioral Therapy” OR TI “Coping strategy” OR AB “Coping strategy” OR TI Mindfulness OR AB Mindfulness OR TI EMDR OR AB EMDR OR TI “Stress recovery” OR AB “Stress recovery” OR TI “Behavioral activation” OR AB “Behavioral activation” OR TI “Emotion regulation” OR AB “Emotion regulation” OR TI Relaxation OR AB Relaxation OR TI “Physical Activity” OR AB “Physical Activity” OR TI “Psychological First Aid” OR AB “Psychological First Aid” OR TI “Cognitive Processing Therapy” OR AB “Cognitive Processing Therapy” OR TI EMDR OR AB EMDR OR TI PFA OR AB PFA OR DE “Resilience (Psychological)” OR DE “Social Support” OR DE “Self-Care” OR DE Psychoeducation OR DE “Coping Style” OR DE Mindfulness OR DE “Mindfulness-Based Interventions” OR DE “Eye Movement Desensitization Therapy” OR DE Relaxation OR DE “Physical Activity” OR DE “Psychological First Aid” OR DE “Cognitive Processing Therapy” OR DE “Cognitive Behavior Therapy”) |
|  | **AND** |  |
|  | Implement* [TIAB]  OR  “Implementation Science” [TIAB]  OR  “Quality Improvement*” [TIAB]  OR  Diffusion [TIAB]  OR  “Diffusion of innovation” [TIAB]  OR  “Knowledge translation” [TIAB]  OR  “Knowledge exchange” [TIAB]  OR  Facilitators [TIAB]  OR  Barriers [TIAB]  OR  “Process evaluation*” [TIAB]  OR  “Formative evaluation*” [TIAB]  OR  “Summative evaluation*” [TIAB]  OR  “Qualitative evaluation*” [TIAB]  OR  Sustainability [TIAB]  OR  Practicability [TIAB]  OR  Feasibility [TIAB]  OR  Fidelity [TIAB]  OR  Maintenance [TIAB]  OR  Disseminat* [TIAB]  OR  Promot* [TIAB]  OR  “Health Plan Implementation” [MH]  OR  “Implementation Science” [MH]  OR  “Quality improvement” [MH]  OR  “Diffusion of Innovation” [MH] | (TI Implement* OR AB Implement* OR TI “Implementation Science” OR AB “Implementation Science” OR TI “Quality Improvement*” OR AB “Quality Improvement* OR TI Diffusion OR AB Diffusion OR TI “Diffusion of innovation” OR AB “Diffusion of innovation” OR TI “Knowledge translation” OR AB “Knowledge translation” OR TI “Knowledge exchange” OR AB “Knowledge exchange” OR TI Facilitators OR AB Facilitators OR TI Barriers OR AB Barriers OR TI “Process evaluation*” OR AB “Process evaluation*” OR TI “Formative evaluation*” OR AB “Formative evaluation*” OR TI “Summative evaluation*” OR AB “Summative evaluation*” OR TI “Qualitative evaluation*” OR AB “Qualitative evaluation*” OR TI Sustainability OR AB Sustainability OR TI Practicability OR AB Practicability OR TI Feasibility OR AB Feasibility OR TI Fidelity OR AB Fidelity OR TI Maintenance OR AB Maintenance OR TI Disseminat* OR AB Dissemina* OR TI Promot* OR AB Promot* OR DE Sustainability) |
| **Context** | Hospital* [TIAB]  OR  “acute hospital” [TIAB]  OR  clinic* [TIAB]  OR  “acute setting” [TIAB]  OR  “hospital setting” [TIAB]  OR  Hospitals [MH] | (TI Hospital* OR AB Hospital* OR TI “acute hospital” OR AB “acute hospital” OR TI clinic* OR AB clinic* OR TI “acute setting” OR AB “acute setting” OR TI “hospital setting” OR AB “hospital setting” OR DE Hospitals OR DE Clinics OR DE Treatment Facilities) |
|  | **AND** | **AND** |
|  | COVID-19 [TIAB]  OR  “COVID-19 pandemic” [TIAB]  OR  COVID-19 [MH] | (TI COVID-19 OR AB COVID-19 OR TI “Covid-19 pandemic” OR AB “COVID-19 pandemic” OR DE COVID-19) |

# 7 Review search strings

The development and review of the search strings for MEDLINE via PubMed and PsychINFO via EBSCO, is done using the Peer Review of Electronic Search Strategies (PRESS) (McGowan et al., 2016). This tool is recommended by Nordhausen and Hirt (2022) for reviewing search strings. The authors describe that not having an external person doing the review resulted in a less fineness. However, they also emphasize that with a high degree of (self-) reflection and conscientiousness, this consequence can be minimized to a large extent (Nordhausen & Hirt, 2022). In addition, the syntax was discussed with the first supervisor (MR) and second supervisor (DH).

At some points rationales are given to make the decisions behind the answers transparent and to show the reflective approach.

The search strings were discussed with the first and second supervisor (MR/ DH).

**Search string validate**

Datum: 15^th^May 2023

Translation research question(s)

Does the search strategy fit my research question?

Yes
  No

Are the search components clearly defined and delimited from each other?

Yes
  No

Are there too few or too many search components?

Yes
  No

*Rationale* In component **C**(oncept) it´s important to have to different components to the objective, respectively. Same in component **C**(ontext).

Are the search components too specific or too sensitive?

Yes
  No

Are there too few or too many search hits?

Yes
  No

*Rationale* The rehearsal led to 20 hits in MEDLINE via PubMed. With the background information as results of the initially systematic literature search to identify evidence-based interventions for symptoms of PTSD in context of the COVID-19 pandemic, this low number of hits is not particular. In addition, the specific type of search strategy was also a reason.

Operators

Are the operators used correctly?

Yes
  No
  Not applicable

Are the brackets in the search string placed correctly?

Yes
  No
  Not applicable

Is it likely that operator NOT will lead to exclusion of potentially relevant publications?

Yes
  No
  Not applicable

*Rationale* It was not necessary to use the operator NOT.

Could the use of word spacing operators lead to a more precise search result?

Yes
  No
  Not applicable

*Rationale* Word spacing operators cannot be used in MEDLINE via PubMed and in PsychINFO via EBSCO lead the use of spacing operators to slightly number of hits.

Were word spacing operators used with an appropriate word spacing number?

Yes
  No
  Not applicable

Keywords (MeSH-terms/ Index terms)

Are all keywords relevant?

Yes
  No
  Not applicable

Are relevant keywords missing?

Yes
  No
  Not applicable

Are subordinate or superordinate keywords too broad or too close?

Yes
  No
  Not applicable

Were keywords properly expanded to include underlying keywords?

Yes
  No
  Not applicable

*Rationale* In MEDLINE via PubMed with the “search command” [MH] all subheadings are included as well as underlying keywords. In PsychINFO via EBSCO every single keyword has to list separate with the “search command” [DE].

Were the subheadings correctly assigned to the keywords?

Yes
  No
  Not applicable

Have keywords and catchwords been defined for each search component?

Yes
  No
  Not applicable

Keywords

Do all keywords contain all **relevant** word combinations?

Yes
  No
  Not applicable

Are all synonyms defined?

Yes
  No
  Not applicable

Are placeholders inserted correctly?

Yes
  No
  Not applicable

Is the truncation/ “Wildcard” placed too early or too late?

Yes
  No
  Not applicable

Are acronyms and abbreviations used appropriately and are the spelled-out terms also integrated?

Yes
  No
  Not applicable

*Rationale* In the specific case of the search term “Eye Movement Desensitization and Preprocessing Therapy” this spelled-out term led to zero hits. As a result, this search term was excluded, but included as a keyword (MeSH-term). The abbreviations and the keyword led to many hits, so these were included in the syntax.

Are the keywords too specific or too sensitive?

Yes
  No
  Not applicable

Are the “search commands” set correctly?

Yes
  No
  Not applicable

Notation, syntax and number of lines

Are there any spelling mistakes?

Yes
  No

Are there any errors in the syntax?

Yes
  No

Have all search components been integrated into the search string?

Yes
  No

Limitations and search filters

Were limits and/ or search filters used appropriately and correctly?

Yes

No
  Not applicable

Are the limitations and/ or search filers indexed in the respective database?

Yes
  No
  Not applicable

Are limits and/ or search filters missing?

Yes
  No
  Not applicable

*Rationale* The criteria “source” leaves open, because of low number of resulted hits in the two databases. This criterion was considered in the Title-Abstract- and full-text-screening.

Results from the validation

After single validation by the first author herself, on 17^th^May 2023, by MR validates the search as well. As one of the main points of the discussion, was removing search terms related to the components of identified interventions. For reason, that already search terms related to the intervention and PTSD exist, it is not necessary to include single components of the identified interventions. Therefore, search terms based on the components were all removed.

# 8 Conducting and documentation of the search

The search filters listed in the following table are applied for the search in the subject databases MEDLINE via PubMed and CINAHL, partly derived from the inclusion and exclusion criteria:

Table 8: Documentation of the search (own visualization based on Nordhausen and Hirt (2022))

| **Search filter** |  |
| --- | --- |
| **Publication Date** | 2020-2023 |
| **Language** | English, German |
| **Spezies** | Human |

Search in MEDLINE via PubMed conducted at 18^th^May2023 (search string is record online)

Table 9: Documentation of the search (own visualization based on Nordhausen and Hirt (2022))

| Search component | Search number | Feed | Number of hits | Search string for MEDLINE via PubMed |
| --- | --- | --- | --- | --- |
| Population | 1 | **Physician* [TIAB]** | 457,052 | ((((((((((((((((((Physician* [TIAB]) OR (Doctor [TIAB])) OR ("Physician Assistant" [TIAB])) OR (Nurs* [TIAB])) OR ("Nursing staff" [TIAB])) OR (Caregiver* [TIAB])) OR ("Registered nurse" [TIAB])) OR ("Nurse practitioner" [TIAB])) OR ("Advanced practice nurse" [TIAB])) OR ("health care workers" [TIAB])) OR ("health workers" [TIAB])) OR ("health-care workers" [TIAB])) OR ("health care professionals" [TIAB])) OR ("frontline healthcare workers" [TIAB])) OR ("Nurse Clinicians" [MH])) OR ("Physician Assistants" [MH])) OR ("Nursing Staff, Hospital" [MH])) OR ("Health Personnel" [MH])) OR ("Nurse Specialists" [MH]) |
|  | 2 | **Doctor [TIAB]** | 67,261 |  |
|  | 3 | **"Physician Assistant" [TIAB]** | 1,969 |  |
|  | 4 | **Nurs* [TIAB]** | 529,910 |  |
|  | 5 | **"Nursing staff" [TIAB]** | 14,604 |  |
|  | 6 | **Caregiver* [TIAB]** | 90,423 |  |
|  | 7 | **"Registered nurse" [TIAB]** | 4,315 |  |
|  | 8 | **"Nurse practitioner" [TIAB]** | 7,031 |  |
|  | 9 | **"Advanced practice nurse" [TIAB]** | 1,127 |  |
|  | 10 | **"health care workers" [TIAB]** | 17,249 |  |
|  | 11 | **"health workers" [TIAB]** | 21,810 |  |
|  | 12 | **"health-care workers" [TIAB]** | 17,249 |  |
|  | 13 | **"health care professionals" [TIAB]** | 28,292 |  |
|  | 14 | **"frontline healthcare workers" [TIAB]** | 512 |  |
|  | 15 | **"Nurse Clinicians" [MH]** | 8,510 |  |
|  | 16 | **"Physician Assistants" [MH]** | 6,407 |  |
|  | 17 | **"Nursing Staff, Hospital" [MH]** | 47,910 |  |
|  | 18 | **"Health Personnel" [MH]** | 608,489 |  |
|  | 19 | **"Nurse Specialists" [MH]** | 19,505 |  |
|  | 20 | **#1 OR #2 OR #3 OR #4 OR #5 OR #6 OR #7 OR #8 OR #9 OR #10 OR #11 OR #12 OR #13 OR #14 OR #15 OR #16 OR #17 OR #18 OR #19** | **1,454,591** |  |

| Search component | Search number | Feed | Number of hits | Search string for MEDLINE via PubMed |
| --- | --- | --- | --- | --- |
| Concept of interest | 21 | **Intervention* [TIAB]** | 1,313,630 |  |
|  | 22 | **"post-traumatic stress disorder" [TIAB]** | 16,110 | (((((("post-traumatic stress disorder" [TIAB]) OR (PTSD [TIAB])) OR ("Acute Stress Disorder" [TIAB])) OR ("Psychological Distress" [TIAB])) OR ("Stress Disorders, Post-Traumatic" [MH])) OR ("Psychological Distress" [MH])) OR ("Stress Disorders, Traumatic, Acute" [MH]) |
|  | 23 | **PTSD [TIAB]** | 33,150 |  |
|  | 24 | **"Acute Stress Disorder" [TIAB]** | 780 |  |
|  | 25 | **"Psychological Distress" [TIAB]** | 27,735 |  |
|  | 26 | **"Stress Disorders, Post-Traumatic" [MH]** | 40,882 |  |
|  | 27 | **"Psychological Distress" [MH]** | 6,668 |  |
|  | 28 | **"Stress Disorders, Traumatic, Acute" [MH]** | 536 |  |
|  | 29 | #22 OR #23 OR #24 OR #25 OR #26 OR #27 OR #28 | **84,446** |  |
|  | 30 | #21 AND #29 | **17,425** | (Intervention* [TIAB]) AND ((((((("post-traumatic stress disorder" [TIAB]) OR (PTSD [TIAB])) OR ("Acute Stress Disorder" [TIAB])) OR ("Psychological Distress" [TIAB])) OR ("Stress Disorders, Post-Traumatic" [MH])) OR ("Psychological Distress" [MH])) OR ("Stress Disorders, Traumatic, Acute" [MH])) |

| Search components | Search number | feed | Number of hits | Search string for MEDLINE via PubMed |
| --- | --- | --- | --- | --- |
| Concept of interest | 31 | **Implement* [TIAB]** | 684,498 | (((((((((((((((((((((((Implement* [TIAB]) OR ("Implementation Science" [TIAB])) OR ("Quality Improvement*" [TIAB])) OR (Diffusion [TIAB])) OR ("Diffusion of innovation" [TIAB])) OR ("Knowledge translation" [TIAB])) OR ("Knowledge exchange" [TIAB])) OR (Facilitators [TIAB])) OR (Barriers [TIAB])) OR ("Process evaluation*" [TIAB])) OR ("Formative evaluation*" [TIAB])) OR ("Summative evaluation*" [TIAB])) OR ("Qualitative evaluation*" [TIAB])) OR (Sustainability [TIAB])) OR (Practicability [TIAB])) OR (Feasibility [TIAB])) OR (Fidelity [TIAB])) OR (Maintenance [TIAB])) OR (Disseminat* [TIAB])) OR (Promot* [TIAB])) OR ("Health Plan Implementation" [MH])) OR ("Implementation Science" [MH])) OR ("Quality improvement" [MH])) OR ("Diffusion of Innovation" [MH]) |
|  | 32 | **"Implementation Science" [TIAB]** | 5,245 |  |
|  | 33 | **"Quality Improvement*" [TIAB]** | 53,717 |  |
|  | 34 | **Diffusion [TIAB]** | 242,589 |  |
|  | 35 | **"Diffusion of innovation" [TIAB]** | 690 |  |
|  | 36 | **"Knowledge translation" [TIAB]** | 4,372 |  |
|  | 37 | **"Knowledge exchange" [TIAB]** | 898 |  |
|  | 38 | **Facilitators [TIAB]** | 25,284 |  |
|  | 39 | **Barriers [TIAB]** | 190,436 |  |
|  | 40 | **"Process evaluation*" [TIAB]** | 5,545 |  |
|  | 41 | **"Formative evaluation*" [TIAB]** | 1,379 |  |
|  | 42 | **"Summative evaluation*" [TIAB]** | 437 |  |
|  | 43 | **"Qualitative evaluation*" [TIAB]** | 5,356 |  |
|  | 44 | **Sustainability [TIAB]** | 42,618 |  |
|  | 45 | **Practicability [TIAB]** | 5,730 |  |
|  | 46 | **Feasibility [TIAB]** | 239,889 |  |
|  | 47 | **Fidelity [TIAB]** | 37,211 |  |
|  | 48 | **Maintenance [TIAB]** | 322,167 |  |
|  | 49 | **Disseminat* [TIAB]** | 161,229 |  |
|  | 50 | **Promot* [TIAB]** | 1,317,353 |  |
|  | 51 | **"Health Plan Implementation" [MH]** | 6,631 |  |
|  | 52 | **"Implementation Science" [MH]** | 1,257 |  |
|  | 53 | **"Quality improvement" [MH]** | 33,383 |  |
|  | 54 | **"Diffusion of Innovation" [MH]** | 21,478 |  |
|  | 55 | **#31 OR #32 OR #33 OR #34 OR #35 OR #36 OR #37 OR #38 OR #39 OR #40 OR #41 O #42 OR #43 OR #44 OR #45 OR #46 OR #47 OR #48 OR #49 OR #50 OR #51 OR #52 OR #53 OR #54** | **3,049,592** |  |

| Search components | Search number | Feed | Number of hits | Search string for MEDLINE via PubMed |
| --- | --- | --- | --- | --- |
|  | 56 | **#30 AND #55** | **4,150** | ((Intervention* [TIAB]) AND ((((((("post-traumatic stress disorder" [TIAB]) OR (PTSD [TIAB])) OR ("Acute Stress Disorder" [TIAB])) OR ("Psychological Distress" [TIAB])) OR ("Stress Disorders, Post-Traumatic" [MH])) OR ("Psychological Distress" [MH])) OR ("Stress Disorders, Traumatic, Acute" [MH]))) AND ((((((((((((((((((((((((Implement* [TIAB]) OR ("Implementation Science" [TIAB])) OR ("Quality Improvement*" [TIAB])) OR (Diffusion [TIAB])) OR ("Diffusion of innovation" [TIAB])) OR ("Knowledge translation" [TIAB])) OR ("Knowledge exchange" [TIAB])) OR (Facilitators [TIAB])) OR (Barriers [TIAB])) OR ("Process evaluation*" [TIAB])) OR ("Formative evaluation*" [TIAB])) OR ("Summative evaluation*" [TIAB])) OR ("Qualitative evaluation*" [TIAB])) OR (Sustainability [TIAB])) OR (Practicability [TIAB])) OR (Feasibility [TIAB])) OR (Fidelity [TIAB])) OR (Maintenance [TIAB])) OR (Disseminat* [TIAB])) OR (Promot* [TIAB])) OR ("Health Plan Implementation" [MH])) OR ("Implementation Science" [MH])) OR ("Quality improvement" [MH])) OR ("Diffusion of Innovation" [MH])) |

| Search components | Search number | Feed | Number of hits | Search string for MEDLINE via PubMed |
| --- | --- | --- | --- | --- |
| Context | 57 | **Hospital* [TIAB]** | 1,614,971 | ((((((Hospital* [TIAB]) OR ("acute hospital" [TIAB])) OR (clinic* [TIAB])) OR ("acute setting" [TIAB])) OR ("hospital setting" [TIAB])) OR (Hospitals [MH])) OR ("Subacute care" [MH]) |
|  | 58 | **"acute hospital" [TIAB]** | 4,330 |  |
|  | 59 | **clinic* [TIAB]** | 5,191,319 |  |
|  | 60 | **"acute setting" [TIAB]** | 2,708 |  |
|  | 61 | **"hospital setting" [TIAB]** | 14,247 |  |
|  | 62 | **Hospitals [MH]** | 315,762 |  |
|  | 63 | **"Subacute care" [MH]** | 1,414 |  |
|  | 64 | **#57 OR #58 OR #59 OR #60 OR #61 OR #62 OR #63** | **6,300,831** |  |
|  | 65 | **COVID-19 [TIAB]** | 313,947 | ((COVID-19 [TIAB]) OR ("COVID-19 pandemic" [TIAB])) OR (COVID-19 [MH]) |
|  | 66 | **"COVID-19 pandemic" [TIAB]** | 118,728 |  |
|  | 67 | **COVID-19 [MH]** | 222,576 |  |
|  | 68 | **#65 OR #66 OR #67** | **342,367** |  |
|  | 69 | **#64 AND #68** | 119,812 | (((((((Hospital* [TIAB]) OR ("acute hospital" [TIAB])) OR (clinic* [TIAB])) OR ("acute setting" [TIAB])) OR ("hospital setting" [TIAB])) OR (Hospitals [MH])) OR ("Subacute care" [MH])) AND (((COVID-19 [TIAB]) OR ("COVID-19 pandemic" [TIAB])) OR (COVID-19 [MH])) |

| Search component | Search number | Feed | Number of hits | Search string for MEDLINE via PubMed |
| --- | --- | --- | --- | --- |
|  | 70 | **#20 AND #56 AND #69** | **63** | ((((((((((((((((((((Physician* [TIAB]) OR (Doctor [TIAB])) OR ("Physician Assistant" [TIAB])) OR (Nurs* [TIAB])) OR ("Nursing staff" [TIAB])) OR (Caregiver* [TIAB])) OR ("Registered nurse" [TIAB])) OR ("Nurse practitioner" [TIAB])) OR ("Advanced practice nurse" [TIAB])) OR ("health care workers" [TIAB])) OR ("health workers" [TIAB])) OR ("health-care workers" [TIAB])) OR ("health care professionals" [TIAB])) OR ("frontline healthcare workers" [TIAB])) OR ("Nurse Clinicians" [MH])) OR ("Physician Assistants" [MH])) OR ("Nursing Staff, Hospital" [MH])) OR ("Health Personnel" [MH])) OR ("Nurse Specialists" [MH])) AND (((Intervention* [TIAB]) AND ((((((("post-traumatic stress disorder" [TIAB]) OR (PTSD [TIAB])) OR ("Acute Stress Disorder" [TIAB])) OR ("Psychological Distress" [TIAB])) OR ("Stress Disorders, Post-Traumatic" [MH])) OR ("Psychological Distress" [MH])) OR ("Stress Disorders, Traumatic, Acute" [MH]))) AND ((((((((((((((((((((((((Implement* [TIAB]) OR ("Implementation Science" [TIAB])) OR ("Quality Improvement*" [TIAB])) OR (Diffusion [TIAB])) OR ("Diffusion of innovation" [TIAB])) OR ("Knowledge translation" [TIAB])) OR ("Knowledge exchange" [TIAB])) OR (Facilitators [TIAB])) OR (Barriers [TIAB])) OR ("Process evaluation*" [TIAB])) OR ("Formative evaluation*" [TIAB])) OR ("Summative evaluation*" [TIAB])) OR ("Qualitative evaluation*" [TIAB])) OR (Sustainability [TIAB])) OR (Practicability [TIAB])) OR (Feasibility [TIAB])) OR (Fidelity [TIAB])) OR (Maintenance [TIAB])) OR (Disseminat* [TIAB])) OR (Promot* [TIAB])) OR ("Health Plan Implementation" [MH])) OR ("Implementation Science" [MH])) OR ("Quality improvement" [MH])) OR ("Diffusion of Innovation" [MH])))) AND ((((((((Hospital* [TIAB]) OR ("acute hospital" [TIAB])) OR (clinic* [TIAB])) OR ("acute setting" [TIAB])) OR ("hospital setting" [TIAB])) OR (Hospitals [MH])) OR ("Subacute care" [MH])) AND (((COVID-19 [TIAB]) OR ("COVID-19 pandemic" [TIAB])) OR (COVID-19 [MH]))) |

| Search component | Search number | Feed | Number of hits | Search string for MEDLINE via PubMed |
| --- | --- | --- | --- | --- |
|  | 71 | ***#70 AND Filter: Humans, English, German*** | **39** | ((((((((((((((((((((Physician* [TIAB]) OR (Doctor [TIAB])) OR ("Physician Assistant" [TIAB])) OR (Nurs* [TIAB])) OR ("Nursing staff" [TIAB])) OR (Caregiver* [TIAB])) OR ("Registered nurse" [TIAB])) OR ("Nurse practitioner" [TIAB])) OR ("Advanced practice nurse" [TIAB])) OR ("health care workers" [TIAB])) OR ("health workers" [TIAB])) OR ("health-care workers" [TIAB])) OR ("health care professionals" [TIAB])) OR ("frontline healthcare workers" [TIAB])) OR ("Nurse Clinicians" [MH])) OR ("Physician Assistants" [MH])) OR ("Nursing Staff, Hospital" [MH])) OR ("Health Personnel" [MH])) OR ("Nurse Specialists" [MH])) AND (((Intervention* [TIAB]) AND ((((((("post-traumatic stress disorder" [TIAB]) OR (PTSD [TIAB])) OR ("Acute Stress Disorder" [TIAB])) OR ("Psychological Distress" [TIAB])) OR ("Stress Disorders, Post-Traumatic" [MH])) OR ("Psychological Distress" [MH])) OR ("Stress Disorders, Traumatic, Acute" [MH]))) AND ((((((((((((((((((((((((Implement* [TIAB]) OR ("Implementation Science" [TIAB])) OR ("Quality Improvement*" [TIAB])) OR (Diffusion [TIAB])) OR ("Diffusion of innovation" [TIAB])) OR ("Knowledge translation" [TIAB])) OR ("Knowledge exchange" [TIAB])) OR (Facilitators [TIAB])) OR (Barriers [TIAB])) OR ("Process evaluation*" [TIAB])) OR ("Formative evaluation*" [TIAB])) OR ("Summative evaluation*" [TIAB])) OR ("Qualitative evaluation*" [TIAB])) OR (Sustainability [TIAB])) OR (Practicability [TIAB])) OR (Feasibility [TIAB])) OR (Fidelity [TIAB])) OR (Maintenance [TIAB])) OR (Disseminat* [TIAB])) OR (Promot* [TIAB])) OR ("Health Plan Implementation" [MH])) OR ("Implementation Science" [MH])) OR ("Quality improvement" [MH])) OR ("Diffusion of Innovation" [MH])))) AND ((((((((Hospital* [TIAB]) OR ("acute hospital" [TIAB])) OR (clinic* [TIAB])) OR ("acute setting" [TIAB])) OR ("hospital setting" [TIAB])) OR (Hospitals [MH])) OR ("Subacute care" [MH])) AND (((COVID-19 [TIAB]) OR ("COVID-19 pandemic" [TIAB])) OR (COVID-19 [MH]))) Filters: Humans, English, German |

Search in PsychINFO via EBSCO conducted at 18^th^May 2023 (search string is record online)

Table 10: Documentation of the search in PsychINFO via EBSCO (own visualization based on Nordhausen and Hirt (2022))

| Search component | Search number | Feed | Number of hits | Search string for PsychINFO via EBSCO |
| --- | --- | --- | --- | --- |
| Population | 1 | TI Physician* | 12,465 |  |
|  | 2 | AB Physician* | 67,236 |  |
|  | 3 | TI Doctor | 4,703 |  |
|  | 4 | AB Doctor | 29,270 |  |
|  | 5 | TI “Doctor of medicine” | 8 |  |
|  | 6 | AB “Doctor of medicine” | 56 |  |
|  | 7 | TI “Physician Assistant” | 114 |  |
|  | 8 | AB “Physician Assistant” | 279 |  |
|  | 9 | TI Nurs* | 47,874 |  |
|  | 10 | AB Nurs* | 109,678 |  |
|  | 11 | TI “Nursing staff” | 577 |  |
|  | 12 | AB “Nursing staff” | 3,696 |  |
|  | 13 | TI Caregiver* | 17,169 |  |
|  | 14 | AB Caregiver* | 57,470 |  |
|  | 15 | TI “Registered nurse” | 247 |  |
|  | 16 | AB “Registered nurse” | 1,140 |  |
|  | 17 | TI “Nurse practitioner” | 510 |  |
|  | 18 | AB “Nurse practitioner” | 1,265 |  |
|  | 19 | TI "Advanced practice nurse” | 53 |  |
|  | 20 | AB “Advanced practice nurse” | 231 |  |
|  | 21 | TI “health care workers” | 546 |  |

| Search component | Search number | Feed | Number of hits | Search string for PsychINFO via EBSCO |
| --- | --- | --- | --- | --- |
|  | 22 | AB “health care workers” | 2,453 | (TI Physician* OR AB Physician* OR TI Doctor OR AB Doctor OR TI “Doctor of medicine” OR AB “Doctor of medicine” OR TI “Physician Assistant” OR AB “Physician Assistant” OR TI Nurs* OR AB Nurs* OR TI “Nursing staff” OR AB “Nursing staff” OR TI Caregiver* OR AB Caregiver* OR TI “Registered nurse” OR AB “Registered nurse” OR TI “Nurse practitioner” OR AB “Nurse practitioner” OR TI “Advanced practice nurse” OR AB “Advanced practice nurse” OR TI “health care workers” OR AB “health care workers” OR TI “health workers” OR AB “health workers” OR TI “health-care workers” OR AB “health-care workers” OR TI “health care professionals” OR AB “health care professionals” OR TI “frontline healthcare workers” OR AB “frontline healthcare workers” OR DE Health Personnel OR DE Physicians OR DE Medical Personnel OR DE Clinicians OR DE Nurses OR DE Caregivers OR DE Professional Personnel OR DE Frontline Employees) |
|  | 23 | TI “health workers” | 987 |  |
|  | 24 | AB “health workers” | 4,991 |  |
|  | 25 | TI “health-care workers” | 546 |  |
|  | 26 | AB “health-care workers” | 2,453 |  |
|  | 27 | TI “health care professionals” | 948 |  |
|  | 28 | AB “health care professionals” | 9,288 |  |
|  | 29 | TI “frontline healthcare workers” | 42 |  |
|  | 30 | AB “frontline healthcare workers” | 86 |  |
|  | 31 | DE Health Personnel | 26,334 |  |
|  | 32 | DE Physicians | 27,192 |  |
|  | 33 | DE Medical Personnel | 6,595 |  |
|  | 34 | DE Clinicians | 13,271 |  |
|  | 35 | DE Nurses | 33,728 |  |
|  | 36 | DE Caregivers | 42,237 |  |
|  | 37 | DE Professional Personnel | 5,547 |  |
|  | 38 | DE Frontline Employees | 224 |  |
|  | 39 | #1 OR #2 OR #3 OR #4 Or #5 Or #6 Or #7 OR #8 Or #9 OR #10 OR #11 OR #12 OR #13 OR #14 Or #15 Or #16 Or #17 OR #18 OR #19 Or #20 OR #21 OR #22 OR #23 OR #24 OR #25 OR #26 OR #27 OR #28 OR #29 OR #30 OR #31 OR #32 OR #33 OR #34 OR #35 OR #36 OR #37 OR #38 | **308,729** |  |

| Search component | Search number | Feed | Number of hits | Search string for PsychINFO via EBSCO |
| --- | --- | --- | --- | --- |
| Concept of interest | 40 | TI intervention* | 86,567 | (TI intervention* OR AB intervention* OR DE Intervention) |
|  | 41 | AB intervention* | 451,961 |  |
|  | 42 | DE Intervention | 86,600 |  |
|  | 43 | #40 OR #41 OR #42 | **469,081** |  |
|  | 44 | TI “post-traumatic stress disorder” | 4,053 | (TI “post-traumatic stress disorder” OR AB “post-traumatic stress disorder” OR TI PTSD OR AB PTSD OR TI “Acute Stress Disorder” OR AB “Acute Stress Disorder” OR TI “Psychological Distress” OR AB “Psychological Distress” OR DE “Posttraumatic Stress Disorder” OR DE “Posttraumatic Stress” OR DE Trauma OR DE “Acute Stress Disorder” OR DE “Stress and Trauma related disorders” OR DE “Caregiver burden”) |
|  | 45 | AB “post-traumatic stress disorder” | 11,204 |  |
|  | 46 | TI PTSD | 8,879 |  |
|  | 47 | AB PTSD | 37,775 |  |
|  | 48 | TI “Acute Stress Disorder” | 270 |  |
|  | 49 | AB “Acute Stress Disorder” | 834 |  |
|  | 50 | TI “Psychological Distress” | 5,850 |  |
|  | 51 | AB “Psychological Distress” | 22,090 |  |
|  | 52 | DE “Posttraumatic Stress Disorder” | 39,502 |  |
|  | 53 | DE “Posttraumatic Stress” | 1,673 |  |
|  | 54 | DE Trauma | 29,146 |  |
|  | 55 | DE “Acute Stress Disorder” | 678 |  |
|  | 56 | DE “Stress and Trauma related disorders” | 48 |  |
|  | 57 | DE “Caregiver burden” | 7,131 |  |
|  | 58 | #44 OR #45 OR #46 OR #48 OR #49 OR #50 OR #51 OR #52 OR #53 OR #54 OR #55 OR #56 OR #57 | **100,491** |  |
|  | 59 | #43 AND #58 | **20,709** | ((TI intervention* OR AB intervention* OR DE Intervention) AND (TI “post-traumatic stress disorder” OR AB “post-traumatic stress disorder” OR TI PTSD OR AB PTSD OR TI “Acute Stress Disorder” OR AB “Acute Stress Disorder” OR TI “Psychological Distress” OR AB “Psychological Distress” OR DE “Posttraumatic Stress Disorder” OR DE “Posttraumatic Stress” OR DE Trauma OR DE “Acute Stress Disorder” OR DE “Stress and Trauma related disorders” OR DE “Caregiver burden”)) |
| Search component | **Search number** | **Feed** | **Number of hits** | **Search string for PsychINFO via EBSCO** |
| Concept of interest | 60 | TI Implement* | 23,995 |  |
|  | 61 | AB Implement* | 212,288 |  |
|  | 62 | TI “Implementation Science” | 250 |  |
|  | 63 | AB “Implementation Science” | 948 |  |
|  | 64 | TI “Quality Improvement*” | 1,287 |  |
|  | 65 | AB “Quality Improvement*" | 5,778 |  |
|  | 66 | TI Diffusion | 5,161 |  |
|  | 67 | AB Diffusion | 21,139 |  |
|  | 68 | TI “Diffusion of innovation” | 64 |  |
|  | 69 | AB “Diffusion of innovation” | 703 |  |
|  | 70 | TI “Knowledge translation” | 299 |  |
|  | 71 | AB “Knowledge translation” | 903 |  |
|  | 72 | TI “Knowledge exchange” | 134 |  |
|  | 73 | AB “Knowledge exchange” | 546 |  |
|  | 74 | TI Facilitators | 2,933 |  |
|  | 75 | AB Facilitators | 15,878 |  |
|  | 76 | TI Barriers | 14,274 |  |
|  | 77 | AB Barriers | 88,119 |  |
|  | 7 | TI “Process evaluation*” | 689 |  |
|  | 79 | AB “Process evaluation*” | 1,799 |  |
|  | 80 | TI “Formative evaluation*” | 338 |  |
|  | 81 | AB “Formative evaluation*” | 906 |  |
|  | 82 | TI “Summative evaluation*” | 45 |  |
|  | 83 | AB “Summative evaluation*” | 412 |  |
|  | 84 | TI “Qualitative evaluation*” | 466 |  |
|  | 85 | AB “Qualitative evaluation*” | 966 |  |
|  | 86 | TI Sustainability | 3,163 |  |
|  | 87 | AB Sustainability | 12,296 |  |
|  | 88 | TI Practicability | 34 |  |
|  | 89 | AB Practicability | 538 |  |
| Search component | **Seach number** | **Feed** | **Number of hits** | **Search string for PsychINFO via EBSCO** |
|  | 90 | TI Feasibility | 6,539 | (TI Implement* OR AB Implement* OR TI “Implementation Science” OR AB “Implementation Science” OR TI “Quality Improvement*” OR AB “Quality Improvement*" OR TI Diffusion OR AB Diffusion OR TI “Diffusion of innovation” OR AB “Diffusion of innovation” OR TI “Knowledge translation” OR AB “Knowledge translation” OR TI “Knowledge exchange” OR AB “Knowledge exchange” OR TI Facilitators OR AB Facilitators OR TI Barriers OR AB Barriers OR TI “Process evaluation*” OR AB “Process evaluation*” OR TI “Formative evaluation*” OR AB “Formative evaluation*” OR TI “Summative evaluation*” OR AB “Summative evaluation*” OR TI “Qualitative evaluation*” OR AB “Qualitative evaluation*” OR TI Sustainability OR AB Sustainability OR TI Practicability OR AB Practicability OR TI Feasibility OR AB Feasibility OR TI Fidelity OR AB Fidelity OR TI Maintenance OR AB Maintenance OR TI Disseminat* OR AB Disseminat* OR TI Promot* OR AB Promot* OR DE Sustainability) |
|  | 91 | AB Feasibility | 27,506 |  |
|  | 92 | TI Fidelity | 1,913 |  |
|  | 93 | AB Fidelity | 10,747 |  |
|  | 94 | TI Maintenance | 9,605 |  |
|  | 95 | AB Maintenance | 61,817 |  |
|  | 96 | TI Disseminat* | 1,912 |  |
|  | 97 | AB Disseminat* | 21,130 |  |
|  | 98 | TI Promot* | 29,657 |  |
|  | 99 | AB Promot* | 217,781 |  |
|  | 100 | DE Sustainability | 441 |  |
|  | 101 | #59 OR #60 OR #61 OR #62 OR #63 OR #64 OR #65 OR #66 OR #67 OR #68 OR #69 OR #70 OR #71 OR #72 OR #73 OR #74 OR #75 OR #76 OR #77 OR #78 OR #79 OR #80 OR #81 OR #82 OR #83 OR #84 OR #85 OR #86 OR #87 OR #88 OR #89 OR #90 OR #91 OR #92 OR #93 OR #94 OR #95 OR #96 OR #97 OR #98 OR #99 | **622,488** |  |
|  | 102 | #59 AND #101 | **4,601** | (((TI intervention* OR AB intervention* OR DE Intervention) AND (TI “post-traumatic stress disorder” OR AB “post-traumatic stress disorder” OR TI PTSD OR AB PTSD OR TI “Acute Stress Disorder” OR AB “Acute Stress Disorder” OR TI “Psychological Distress” OR AB “Psychological Distress” OR DE “Posttraumatic Stress Disorder” OR DE “Posttraumatic Stress” OR DE Trauma OR DE “Acute Stress Disorder” OR DE “Stress and Trauma related disorders” OR DE “Caregiver burden”)) AND ((TI Implement* OR AB Implement* OR TI “Implementation Science” OR AB “Implementation Science” OR TI “Quality Improvement*” OR AB “Quality Improvement*" OR TI Diffusion OR AB Diffusion OR TI “Diffusion of innovation” OR AB “Diffusion of innovation” OR TI “Knowledge translation” OR AB “Knowledge translation” OR TI “Knowledge exchange” OR AB “Knowledge exchange” OR TI Facilitators OR AB Facilitators OR TI Barriers OR AB Barriers OR TI “Process evaluation*” OR AB “Process evaluation*” OR TI “Formative evaluation*” OR AB “Formative evaluation*” OR TI “Summative evaluation*” OR AB “Summative evaluation*” OR TI “Qualitative evaluation*” OR AB “Qualitative evaluation*” OR TI Sustainability OR AB Sustainability OR TI Practicability OR AB Practicability OR TI Feasibility OR AB Feasibility OR TI Fidelity OR AB Fidelity OR TI Maintenance OR AB Maintenance OR TI Disseminat* OR AB Disseminat* OR TI Promot* OR AB Promot* OR DE Sustainability))) |

| Search component | | Seach number | Feed | Number of hits | Search string for PsychINFO via EBSCO |
| --- | --- | --- | --- | --- | --- |
| Context | | 103 | TI Hospital* | 36,398 | (TI Hospital* OR AB Hospital* OR TI “acute hospital” OR AB “acute hospital” OR TI clinic* OR AB clinic* OR TI “acute setting” OR AB “acute setting” OR TI “hospital setting” OR AB “hospital setting” OR DE Hospitals OR DE Clinics OR DE Treatment Facilities) |
|  |  | 104 | AB Hospital* | 169,897 |  |
|  |  | 105 | TI “acute hospital” | 224 |  |
|  |  | 106 | AB “acute hospital” | 837 |  |
|  |  | 107 | TI clinic* | 113,090 |  |
|  |  | 108 | AB clinic* | 677,898 |  |
|  |  | 109 | TI “acute setting” | 31 |  |
|  |  | 110 | AB “acute setting” | 264 |  |
|  |  | 111 | TI “hospital setting” | 679 |  |
|  |  | 112 | AB “hospital setting” | 2,647 |  |
|  |  | 113 | DE Hospitals | 19,196 |  |
|  |  | 114 | DE Clinics | 6,931 |  |
|  |  | 115 | DE Treatment Facilities | 2,139 |  |
|  |  | 116 | #103 OR #104 OR #105 OR #106 OR #107 OR #108 OR #109 OR #110 OR #111 OR #112 OR #113 OR #114 OR #115 | **825,702** |  |
|  |  | 117 | TI COVID-19 | 21,741 | (TI COVID-19 OR AB COVID-19 OR TI “COVID-19 pandemic” OR AB “COVID-19 pandemic” OR DE COVID-19) |
|  |  | 118 | AB COVID-19 | 29,551 |  |
|  |  | 119 | TI “COVID-19 pandemic” | 8,265 |  |
|  |  | 120 | AB “COVID-19 pandemic” | 17,319 |  |
|  |  | 121 | DE COVID-19 | 22,240 |  |
|  |  | 122 | #117 OR #118 OR #119 OR #120 OR #121 | **31,517** |  |
|  | | 123 | #116 AND #122 | **6,888** | ((TI Hospital* OR AB Hospital* OR TI “acute hospital” OR AB “acute hospital” OR TI clinic* OR AB clinic* OR TI “acute setting” OR AB “acute setting” OR TI “hospital setting” OR AB “hospital setting” OR DE Hospitals OR DE Clinics OR DE Treatment Facilities) AND (TI COVID-19 OR AB COVID-19 OR TI “COVID-19 pandemic” OR AB “COVID-19 pandemic” OR DE COVID-19)) |
|  | 124 | | #39 AND #102 AND #123 | 29 | ((((TI Physician* OR AB Physician* OR TI Doctor OR AB Doctor OR TI “Doctor of medicine” OR AB “Doctor of medicine” OR TI “Physician Assistant” OR AB “Physician Assistant” OR TI Nurs* OR AB Nurs* OR TI “Nursing staff” OR AB “Nursing staff” OR TI Caregiver* OR AB Caregiver* OR TI “Registered nurse” OR AB “Registered nurse” OR TI “Nurse practitioner” OR AB “Nurse practitioner” OR TI “Advanced practice nurse” OR AB “Advanced practice nurse” OR TI “health care workers” OR AB “health care workers” OR TI “health workers” OR AB “health workers” OR TI “health-care workers” OR AB “health-care workers” OR TI “health care professionals” OR AB “health care professionals” OR TI “frontline healthcare workers” OR AB “frontline healthcare workers” OR DE Health Personnel OR DE Physicians OR DE Medical Personnel OR DE Clinicians OR DE Nurses OR DE Caregivers OR DE Professional Personnel OR DE Frontline Employees) AND (((TI intervention* OR AB intervention* OR DE Intervention) AND (TI “post-traumatic stress disorder” OR AB “post-traumatic stress disorder” OR TI PTSD OR AB PTSD OR TI “Acute Stress Disorder” OR AB “Acute Stress Disorder” OR TI “Psychological Distress” OR AB “Psychological Distress” OR DE “Posttraumatic Stress Disorder” OR DE “Posttraumatic Stress” OR DE Trauma OR DE “Acute Stress Disorder” OR DE “Stress and Trauma related disorders” OR DE “Caregiver burden”)) AND ((TI Implement* OR AB Implement* OR TI “Implementation Science” OR AB “Implementation Science” OR TI “Quality Improvement*” OR AB “Quality Improvement*" OR TI Diffusion OR AB Diffusion OR TI “Diffusion of innovation” OR AB “Diffusion of innovation” OR TI “Knowledge translation” OR AB “Knowledge translation” OR TI “Knowledge exchange” OR AB “Knowledge exchange” OR TI Facilitators OR AB Facilitators OR TI Barriers OR AB Barriers OR TI “Process evaluation*” OR AB “Process evaluation*” OR TI “Formative evaluation*” OR AB “Formative evaluation*” OR TI “Summative evaluation*” OR AB “Summative evaluation*” OR TI “Qualitative evaluation*” OR AB “Qualitative evaluation*” OR TI Sustainability OR AB Sustainability OR TI Practicability OR AB Practicability OR TI Feasibility OR AB Feasibility OR TI Fidelity OR AB Fidelity OR TI Maintenance OR AB Maintenance OR TI Disseminat* OR AB Disseminat* OR TI Promot* OR AB Promot* OR DE Sustainability))) AND ((TI Hospital* OR AB Hospital* OR TI “acute hospital” OR AB “acute hospital” OR TI clinic* OR AB clinic* OR TI “acute setting” OR AB “acute setting” OR TI “hospital setting” OR AB “hospital setting” OR DE Hospitals OR DE Clinics OR DE Treatment Facilities) AND (TI COVID-19 OR AB COVID-19 OR TI “COVID-19 pandemic” OR AB “COVID-19 pandemic” OR DE COVID-19))) |
|  | 125 | | #124 AND *Filter: 2020-2023, Humans, English, German* | **29** |  |

# 9 Supplementary search options

This additional literature research is specific and aimed to identify preferably all possible references regarding the research question. Therefore Cooper et al. (2017) conclude, that several supplementary search option should be conducted after the systematic research in databases.

This research focused on the topic identifying barriers and facilitators that influence the implementation of evidence-based interventions for symptoms of PTSD among hospital-based nurses and physicians during the COVID-19 pandemic.

First a limited systematic search is conducted in CINHAL via EBSCO, because this database contains literature specifically for the field literature from nursing science and related topics from the health care sector (see Table 10). Furthermore, the publications exported to EndNote 20 and searched for duplicates before the additional Title-Abstract-Screening is conducted in Rayyan (Ouzzani et al., 2016). After that a total of four studies were included in full text screening. For detailed description of the literature search, see the flow chart in Supplement 1.

Secondly, during the full text-screening of all included studies, backward was conducted to search in the reference list of each included study for potential publications (e.g., study protocols) and the forward citation tracking was performed in Google scholar.

Search in CINAHL via EBSCO conducted at 6^th^June 2023 (search string is record online)

Table 11: Documentation of the search in CINAHL via EBSCO (own visualization based on Nordhausen and Hirt (2022))

| Search component | Search number | Feed | Number of hits | Search string for PsychINFO via EBSCO |
| --- | --- | --- | --- | --- |
| Population | 1 | TI Physician* | 45,156 |  |
|  | 2 | AB Physician* | 146,164 |  |
|  | 3 | TI Doctor | 21,200 |  |
|  | 4 | AB Doctor | 49,069 |  |
|  | 5 | TI “Doctor of medicine” | 7 |  |
|  | 6 | AB “Doctor of medicine” | 215 |  |
|  | 7 | TI “Physician Assistant” | 1,084 |  |
|  | 8 | AB “Physician Assistant” | 1,272 |  |
|  | 9 | TI Nurs* | 360,218 |  |
|  | 10 | AB Nurs* | 388,341 |  |
|  | 11 | TI “Nursing staff” | 2,178 |  |
|  | 12 | AB “Nursing staff” | 11,362 |  |
|  | 13 | TI Caregiver* | 23,861 |  |
|  | 14 | AB Caregiver* | 55,079 |  |
|  | 15 | TI “Registered nurse” | 1,578 |  |
|  | 16 | AB “Registered nurse” | 4,870 |  |
|  | 17 | TI “Nurse practitioner” | 4,379 |  |
|  | 18 | AB “Nurse practitioner” | 5,646 |  |
|  | 19 | TI "Advanced practice nurse” | 462 |  |
|  | 20 | AB “Advanced practice nurse” | 876 |  |
|  | 21 | TI “health care workers” | 2,591 |  |

| Search component | Search number | Feed | Number of hits | Search string for PsychINFO via EBSCO |
| --- | --- | --- | --- | --- |
|  | 22 | AB “health care workers” | 6,687 | (TI Physician* OR AB Physician* OR TI Doctor OR AB Doctor OR TI “Doctor of medicine” OR AB “Doctor of medicine” OR TI “Physician Assistant” OR AB “Physician Assistant” OR TI Nurs* OR AB Nurs* OR TI “Nursing staff” OR AB “Nursing staff” OR TI Caregiver* OR AB Caregiver* OR TI “Registered nurse” OR AB “Registered nurse” OR TI “Nurse practitioner” OR AB “Nurse practitioner” OR TI “Advanced practice nurse” OR AB “Advanced practice nurse” OR TI “health care workers” OR AB “health care workers” OR TI “health workers” OR AB “health workers” OR TI “health-care workers” OR AB “health-care workers” OR TI “health care professionals” OR AB “health care professionals” OR TI “frontline healthcare workers” OR AB “frontline healthcare workers” OR MH Physicians OR MH “Physician Assistants” OR MH Nurses OR MH "Practical Nurses" OR MH "Nursing Staff, Hospital" OR MH "Staff Nurses" OR MH Caregivers OR MH "Registered Nurses" OR MH "Practical Nurses" OR MH "Nurse Practitioners" OR MH "Advanced Practice Nurses" OR MH "Health Personnel") |
|  | 23 | TI “health workers” | 2,718 |  |
|  | 24 | AB “health workers” | 9,065 |  |
|  | 25 | TI “health-care workers” | 2,591 |  |
|  | 26 | AB “health-care workers” | 6,687 |  |
|  | 27 | TI “health care professionals” | 2,291 |  |
|  | 28 | AB “health care professionals” | 17,589 |  |
|  | 29 | TI “frontline healthcare workers” | 52 |  |
|  | 30 | AB “frontline healthcare workers” | 147 |  |
|  | 31 | MH Physicians | 64,033 |  |
|  | 32 | MH "Physician Assistants" | 5,519 |  |
|  | 33 | MH Nurses | 67,001 |  |
|  | 34 | MH "Practical Nurses" | 5,640 |  |
|  | 35 | MH "Nursing Staff, Hospital" | 24,275 |  |
|  | 36 | MH "Staff Nurses" | 24,275 |  |
|  | 37 | MH Caregivers | 42,321 |  |
|  | 38 | MH "Registered Nurses" | 34,348 |  |
|  | 39 | MH "Practical Nurses" | 5,640 |  |
|  | 40 | MH "Nurse Practitioners" | 19,402 |  |
|  | 41 | MH "Advanced Practice Nurses" | 19,88 |  |
|  | 42 | MH "Health Personnel" | 50,663 |  |
|  | 43 | #1 OR #2 OR #3 OR #4 Or #5 Or #6 Or #7 OR #8 Or #9 OR #10 OR #11 OR #12 OR #13 OR #14 Or #15 Or #16 Or #17 OR #18 OR #19 Or #20 OR #21 OR #22 OR #23 OR #24 OR #25 OR #26 OR #27 OR #28 OR #29 OR #30 OR #31 OR #32 OR #33 OR #34 OR #35 OR #36 OR #37 OR #38 OR #39 OR #40 OR #41 OR #42 | **981,819** |  |

| Search component | Search number | Feed | Number of hits | Search string for PsychINFO via EBSCO |
| --- | --- | --- | --- | --- |
| Concept of interest | 44 | TI intervention* | 118,714 | (TI intervention* OR AB intervention*) |
|  | 45 | AB intervention* | 506,388 |  |
|  | 46 | #44 OR #45 | **553,331** |  |
|  | 47 | TI “post-traumatic stress disorder” | 2,248 | (TI “post-traumatic stress disorder” OR AB “post-traumatic stress disorder” OR TI PTSD OR AB PTSD OR TI “Acute Stress Disorder” OR AB “Acute Stress Disorder” OR TI “Psychological Distress” OR AB “Psychological Distress” OR MH "Stress Disorders, Post-Traumatic" OR MH "Psychological Distress") |
|  | 48 | AB “post-traumatic stress disorder” | 5,886 |  |
|  | 49 | TI PTSD | 4,303 |  |
|  | 50 | AB PTSD | 12,467 |  |
|  | 51 | TI “Acute Stress Disorder” | 124 |  |
|  | 52 | AB “Acute Stress Disorder” | 287 |  |
|  | 53 | TI “Psychological Distress” | 4,265 |  |
|  | 54 | AB “Psychological Distress” | 13,254 |  |
|  | 55 | MH "Stress Disorders, Post-Traumatic" | 26,656 |  |
|  | 56 | MH "Psychological Distress" | 5,099 |  |
|  | 57 | #47 OR #48 OR #49 OR #50 OR #51 OR #52 OR #53 OR #54 OR #55 OR #56 | **47,179** |  |
|  | 58 | #46 AND #57 | **9,799** | ((TI intervention* OR AB intervention* OR DE Intervention) AND (TI “post-traumatic stress disorder” OR AB “post-traumatic stress disorder” OR TI PTSD OR AB PTSD OR TI “Acute Stress Disorder” OR AB “Acute Stress Disorder” OR TI “Psychological Distress” OR AB “Psychological Distress” OR MH "Stress Disorders, Post-Traumatic" OR MH "Psychological Distress") |

| Search component | Search number | Feed | Number of hits | Search string for PsychINFO via EBSCO |
| --- | --- | --- | --- | --- |
| Concept of interest | 59 | TI Implement* | 45,692 |  |
|  | 60 | AB Implement* | 229,007 |  |
|  | 61 | TI “Implementation Science” | 652 |  |
|  | 62 | AB “Implementation Science” | 1,454 |  |
|  | 63 | TI “Quality Improvement*” | 9,931 |  |
|  | 64 | AB “Quality Improvement*" | 22,903 |  |
|  | 65 | TI Diffusion | 8,282 |  |
|  | 66 | AB Diffusion | 19,964 |  |
|  | 67 | TI “Diffusion of innovation” | 83 |  |
|  | 68 | AB “Diffusion of innovation” | 350 |  |
|  | 69 | TI “Knowledge translation” | 1,016 |  |
|  | 70 | AB “Knowledge translation” | 2,034 |  |
|  | 71 | TI “Knowledge exchange” | 120 |  |
|  | 72 | AB “Knowledge exchange” | 447 |  |
|  | 73 | TI Facilitators | 4,915 |  |
|  | 74 | AB Facilitators | 17,974 |  |
|  | 75 | TI Barriers | 26,184 |  |
|  | 76 | AB Barriers | 105,216 |  |
|  | 77 | TI “Process evaluation*” | 1,269 |  |
|  | 78 | AB “Process evaluation*” | 2,874 |  |
|  | 79 | TI “Formative evaluation*” | 249 |  |
|  | 80 | AB “Formative evaluation*” | 711 |  |
|  | 81 | TI “Summative evaluation*” | 29 |  |
|  | 82 | AB “Summative evaluation*” | 267 |  |
|  | 83 | TI “Qualitative evaluation*” | 855 |  |
|  | 84 | AB “Qualitative evaluation*” | 1,324 |  |
|  | 85 | TI Sustainability | 2,495 |  |
|  | 86 | AB Sustainability | 10,939 |  |
|  | 87 | TI Practicability | 65 |  |
|  | 88 | AB Practicability | 546 |  |
| Search component | **Seach number** | **Feed** | **Number of hits** | **Search string for PsychINFO via EBSCO** |
|  | 89 | TI Feasibility | 20,041 | (TI Implement* OR AB Implement* OR TI “Implementation Science” OR AB “Implementation Science” OR TI “Quality Improvement*” OR AB “Quality Improvement*" OR TI Diffusion OR AB Diffusion OR TI “Diffusion of innovation” OR AB “Diffusion of innovation” OR TI “Knowledge translation” OR AB “Knowledge translation” OR TI “Knowledge exchange” OR AB “Knowledge exchange” OR TI Facilitators OR AB Facilitators OR TI Barriers OR AB Barriers OR TI “Process evaluation*” OR AB “Process evaluation*” OR TI “Formative evaluation*” OR AB “Formative evaluation*” OR TI “Summative evaluation*” OR AB “Summative evaluation*” OR TI “Qualitative evaluation*” OR AB “Qualitative evaluation*” OR TI Sustainability OR AB Sustainability OR TI Practicability OR AB Practicability OR TI Feasibility OR AB Feasibility OR TI Fidelity OR AB Fidelity OR TI Maintenance OR AB Maintenance OR TI Disseminat* OR AB Disseminat* OR TI Promot* OR AB Promot* OR MH „Implementation Science“ OR MH „Diffusion of Innovation“ OR MH „Quality Improvement“ OR MH „Formative Evaluation Research“ OR MH „Summative Evaluation Research“ OR MH „Selective Dissemination of Information“) |
|  | 90 | AB Feasibility | 52,407 |  |
|  | 91 | TI Fidelity | 2,151 |  |
|  | 92 | AB Fidelity | 7,663 |  |
|  | 93 | TI Maintenance | 10,792 |  |
|  | 94 | AB Maintenance | 49,687 |  |
|  | 95 | TI Disseminat* | 5,674 |  |
|  | 96 | AB Disseminat* | 28,331 |  |
|  | 97 | TI Promot* | 50,242 |  |
|  | 98 | AB Promot* | 207,850 |  |
|  | 99 | MH „Implementation Science“ | 923 |  |
|  | 100 | MH „Diffusion of Innovation“ | 17,820 |  |
|  | 101 | MH „Quality Improvement“ | 65,470 |  |
|  | 102 | MH „Formative Evaluation Research“ | 539 |  |
|  | 103 | MH „Summative Evaluation Research“ | 116 |  |
|  | 104 | MH „Selective Dissemination of Information“ | 75 |  |
|  | 101 | #59 OR #60 OR #61 OR #62 OR #63 OR #64 OR #65 OR #66 OR #67 OR #68 OR #69 OR #70 OR #71 OR #72 OR #73 OR #74 OR #75 OR #76 OR #77 OR #78 OR #79 OR #80 OR #81 OR #82 OR #83 OR #84 OR #85 OR #86 OR #87 OR #88 OR #89 OR #90 OR #91 OR #92 OR #93 OR #94 OR #95 OR #96 OR #97 OR #98 OR #99 OR 100 OR #101 | **764,109** |  |
|  | 102 | #58 AND #101 | **2,720** | (((TI intervention* OR AB intervention* OR DE Intervention) AND (TI “post-traumatic stress disorder” OR AB “post-traumatic stress disorder” OR TI PTSD OR AB PTSD OR TI “Acute Stress Disorder” OR AB “Acute Stress Disorder” OR TI “Psychological Distress” OR AB “Psychological Distress” OR DE “Posttraumatic Stress Disorder” OR DE “Posttraumatic Stress” OR DE Trauma OR DE “Acute Stress Disorder” OR DE “Stress and Trauma related disorders” OR DE “Caregiver burden”)) AND ((TI Implement* OR AB Implement* OR TI “Implementation Science” OR AB “Implementation Science” OR TI “Quality Improvement*” OR AB “Quality Improvement*" OR TI Diffusion OR AB Diffusion OR TI “Diffusion of innovation” OR AB “Diffusion of innovation” OR TI “Knowledge translation” OR AB “Knowledge translation” OR TI “Knowledge exchange” OR AB “Knowledge exchange” OR TI Facilitators OR AB Facilitators OR TI Barriers OR AB Barriers OR TI “Process evaluation*” OR AB “Process evaluation*” OR TI “Formative evaluation*” OR AB “Formative evaluation*” OR TI “Summative evaluation*” OR AB “Summative evaluation*” OR TI “Qualitative evaluation*” OR AB “Qualitative evaluation*” OR TI Sustainability OR AB Sustainability OR TI Practicability OR AB Practicability OR TI Feasibility OR AB Feasibility OR TI Fidelity OR AB Fidelity OR TI Maintenance OR AB Maintenance OR TI Disseminat* OR AB Disseminat* OR TI Promot* OR AB Promot* OR MH „Implementation Science“ OR MH „Diffusion of Innovation“ OR MH „Quality Improvement“ OR MH „Formative Evaluation Research“ OR MH „Summative Evaluation Research“ OR MH „Selective Dissemination of Information“)) |

| Search component | Seach number | Feed | Number of hits | Search string for PsychINFO via EBSCO |
| --- | --- | --- | --- | --- |
| Context | 103 | TI Hospital* | 159,623 | (TI Hospital* OR AB Hospital* OR TI “acute hospital” OR AB “acute hospital” OR TI clinic* OR AB clinic* OR TI “acute setting” OR AB “acute setting” OR TI “hospital setting” OR AB “hospital setting” OR MH Hospitals) |
|  | 104 | AB Hospital* | 479,712 |  |
|  | 105 | TI “acute hospital” | 873 |  |
|  | 106 | AB “acute hospital” | 2,678 |  |
|  | 107 | TI clinic* | 338,967 |  |
|  | 108 | AB clinic* | 1,138,242 |  |
|  | 109 | TI “acute setting” | 165 |  |
|  | 110 | AB “acute setting” | 965 |  |
|  | 111 | TI “hospital setting” | 1,874 |  |
|  | 112 | AB “hospital setting” | 5,888 |  |
|  | 113 | MH Hospitals | 64,589 |  |
|  | 114 | #103 OR #104 OR #105 OR #106 OR #107 OR #108 OR #109 OR #110 OR #111 OR #112 OR #113 | **1,714,039** |  |
|  | 115 | TI COVID-19 | 86,852 | (TI COVID-19 OR AB COVID-19 OR TI “COVID-19 pandemic” OR AB “COVID-19 pandemic” OR MH COVID-19) |
|  | 116 | AB COVID-19 | 71,641 |  |
|  | 117 | TI “COVID-19 pandemic” | 21,373 |  |
|  | 118 | AB “COVID-19 pandemic” | 32,890 |  |
|  | 119 | MH COVID-19 | 41,343 |  |
|  | 120 | #115 OR #116 OR #117 OR #118 OR #119 | **31,517** |  |
|  | 121 | #114 AND #120 | **33,675** | ((TI Hospital* OR AB Hospital* OR TI “acute hospital” OR AB “acute hospital” OR TI clinic* OR AB clinic* OR TI “acute setting” OR AB “acute setting” OR TI “hospital setting” OR AB “hospital setting” OR MH Hospitals) AND (TI COVID-19 OR AB COVID-19 OR TI “COVID-19 pandemic” OR AB “COVID-19 pandemic” OR MH COVID-19)) |

|  | 122 | #43 AND #102 AND #121 | 20 | ((((TI Physician* OR AB Physician* OR TI Doctor OR AB Doctor OR TI “Doctor of medicine” OR AB “Doctor of medicine” OR TI “Physician Assistant” OR AB “Physician Assistant” OR TI Nurs* OR AB Nurs* OR TI “Nursing staff” OR AB “Nursing staff” OR TI Caregiver* OR AB Caregiver* OR TI “Registered nurse” OR AB “Registered nurse” OR TI “Nurse practitioner” OR AB “Nurse practitioner” OR TI “Advanced practice nurse” OR AB “Advanced practice nurse” OR TI “health care workers” OR AB “health care workers” OR TI “health workers” OR AB “health workers” OR TI “health-care workers” OR AB “health-care workers” OR TI “health care professionals” OR AB “health care professionals” OR TI “frontline healthcare workers” OR AB “frontline healthcare workers” OR MH Physicians OR MH “Physician Assistants” OR MH Nurses OR MH "Practical Nurses" OR MH "Nursing Staff, Hospital" OR MH "Staff Nurses" OR MH Caregivers OR MH "Registered Nurses" OR MH "Practical Nurses" OR MH "Nurse Practitioners" OR MH "Advanced Practice Nurses" OR MH "Health Personnel") AND (((TI intervention* OR AB intervention* OR DE Intervention) AND (TI “post-traumatic stress disorder” OR AB “post-traumatic stress disorder” OR TI PTSD OR AB PTSD OR TI “Acute Stress Disorder” OR AB “Acute Stress Disorder” OR TI “Psychological Distress” OR AB “Psychological Distress” OR DE “Posttraumatic Stress Disorder” OR DE “Posttraumatic Stress” OR DE Trauma OR DE “Acute Stress Disorder” OR DE “Stress and Trauma related disorders” OR DE “Caregiver burden”)) AND ((TI Implement* OR AB Implement* OR TI “Implementation Science” OR AB “Implementation Science” OR TI “Quality Improvement*” OR AB “Quality Improvement*" OR TI Diffusion OR AB Diffusion OR TI “Diffusion of innovation” OR AB “Diffusion of innovation” OR TI “Knowledge translation” OR AB “Knowledge translation” OR TI “Knowledge exchange” OR AB “Knowledge exchange” OR TI Facilitators OR AB Facilitators OR TI Barriers OR AB Barriers OR TI “Process evaluation*” OR AB “Process evaluation*” OR TI “Formative evaluation*” OR AB “Formative evaluation*” OR TI “Summative evaluation*” OR AB “Summative evaluation*” OR TI “Qualitative evaluation*” OR AB “Qualitative evaluation*” OR TI Sustainability OR AB Sustainability OR TI Practicability OR AB Practicability OR TI Feasibility OR AB Feasibility OR TI Fidelity OR AB Fidelity OR TI Maintenance OR AB Maintenance OR TI Disseminat* OR AB Disseminat* OR TI Promot* OR AB Promot* OR MH „Implementation Science“ OR MH „Diffusion of Innovation“ OR MH „Quality Improvement“ OR MH „Formative Evaluation Research“ OR MH „Summative Evaluation Research“ OR MH „Selective Dissemination of Information“))) AND (((TI Hospital* OR AB Hospital* OR TI “acute hospital” OR AB “acute hospital” OR TI clinic* OR AB clinic* OR TI “acute setting” OR AB “acute setting” OR TI “hospital setting” OR AB “hospital setting” OR MH Hospitals) AND (TI COVID-19 OR AB COVID-19 OR TI “COVID-19 pandemic” OR AB “COVID-19 pandemic” OR MH COVID-19))) |
| --- | --- | --- | --- | --- |
|  | 125 | #124 AND *Filter: 2020-2023, Humans, English, German* | **19** |  |

# 10 Title-Abstract- and full-text-screening

All included references were export to EndNote 20 and after screening for duplicates, they were transferred to Rayyan (Ouzzani et al., 2016). There, the title-abstract-, as well as the full-text-screening took place by DK.

To establish methodological quality, four studies were independently reviewed by MR and DH, each. On 23^th^May 2023 the results were discussed and one reference, which thematized a *medical-based intervention* was set on ‘maybe’ by the DH because of unclearly defined inclusion criteria. After the discussion with MR, this reference was included, because the definition of ‘interventions’ was not specified as part of the inclusion criteria. Also, two more references were different categorized. After discussion with the MR these references were included for screening them in full text. Two references (Kunkler, 2023; Zhang et al., 2023) could not provide by any source in English or in full text, either from the journal or the researchers itself.

From 29^th^May until 1^st^June 2023 the full-text-screening was conducted also in Rayyan by DK herself and three references were screened by MR and DH, independently.

The discussion took place on 1^st^June 2023, as well as the alignment with the MR and DH.

As can be seen from the results of the present literature search, that none references match the inclusion criteria. Nevertheless 7 references resulted from the first literature search are removed because of duplication. These correspond with the defined inclusion criteria and were included for the data analysis. Most of the references after the full-text screening were excluded because of lack of hints for influenced factors, such as barriers and facilitators or less information about in general the implementation process of the intervention. There are some hints of some outcomes, such as e.g., acceptability or that participants find the intervention useful. These descriptions could lead to further research focused on implementation matters.

# 12 Supplement I Flowchart


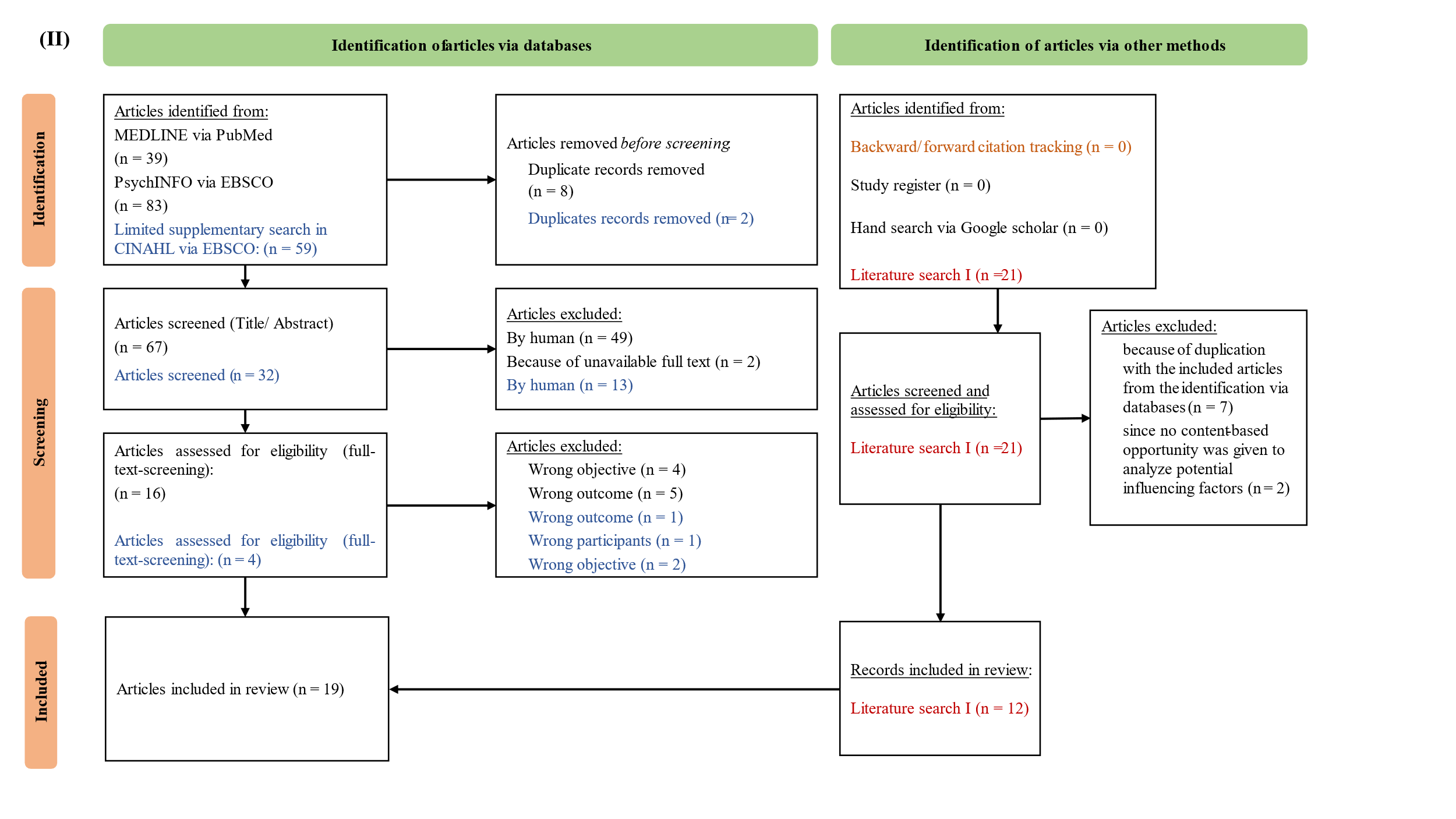


Figure 1: PRISMA-Flowchart of the systematic literature searches in databases according to Page et al., 2020

# 13 Supplement II Bibliographic information of the articles included for the data synthesis

1. Albott, C. S., Wozniak, J. R., McGlinch, B. P., Wall, M. H., Gold, B. S., & Vinogradov, S. (2020). Battle Buddies: Rapid Deployment of a Psychological Resilience Intervention for Health Care Workers During the COVID-19 Pandemic. *Anesth Analg*, *131*(1), 43-54. <https://doi.org/10.1213/ANE.0000000000004912>
2. Blake, H., Bermingham, F., Johnson, G., & Tabner, A. (2020). Mitigating the Psychological Impact of COVID-19 on Healthcare Workers: A Digital Learning Package. *Int J Environ Res Public Health*, *17*(9). <https://doi.org/10.3390/ijerph17092997>
3. Bureau, R., Bemmouna, D., Faria, C. G. F., Goethals, A.-A. C., Douhet, F., Mengin, A. C., Fritsch, A., Zinetti Bertschy, A., Frey, I., & Weiner, L. (2021). My health too: Investigating the feasibility and the acceptability of an internet-based cognitive-behavioral therapy program developed for healthcare workers. *Frontiers in Psychology*, *12*. <https://doi.org/10.3389/fpsyg.2021.760678>
4. Dong, L., Meredith, L. S., Farmer, C. M., Ahluwalia, S. C., Chen, P. G., Bouskill, K., Han, B., Qureshi, N., Dalton, S., Watson, P., Schnurr, P. P., Davis, K., Tobin, J. N., Cassells, A., & Gidengil, C. A. (2022). Protecting the mental and physical well-being of frontline health care workers during COVID-19: Study protocol of a cluster randomized controlled trial. *Contemp Clin Trials*, *117*, 106768. <https://doi.org/10.1016/j.cct.2022.106768>
5. Dumarkaite, A., Truskauskaite, I., Andersson, G., Jovarauskaite, L., Jovaisiene, I., Nomeikaite, A., & Kazlauskas, E. (2023). The efficacy of the internet-based stress recovery intervention FOREST for nurses amid the COVID-19 pandemic: A randomized controlled trial. Int J Nurs Stud, 138, 104408. https://doi.org/doi:10.1016/j.ijnurstu.2022.104408
6. Hannig, C., Lotzin, A., Milin, S., & Schäfer, I. (2021). Stress- und Traumaprävention für Beschäftigte im Gesundheitsbereich = Stress and trauma prevention for employees in the health sector. *Trauma & Gewalt*, *15*(3), 232-242. <https://doi.org/10.21706/tg-15-3-232>
7. Jovarauskaite, L., Dumarkaite, A., Truskauskaite-Kuneviciene, I., Jovaisiene, I., Andersson, G., & Kazlauskas, E. (2021). Internet-based stress recovery intervention FOREST for healthcare staff amid COVID-19 pandemic: study protocol for a randomized controlled trial. *Trials*, *22*(1), 559. <https://doi.org/10.1186/s13063-021-05512-1>
8. Kanellopoulos, D., Solomonov, N., Ritholtz, S., Wilkins, V., Goldman, R., Schier, M., Oberlin, L., Bueno-Castellano, C., Dargis, M., Cherestal, S., & Gunning, F. (2021). The CopeNYP program: A model for brief treatment of psychological distress among healthcare workers and hospital staff. *Gen Hosp Psychiatry*, *73*, 24-29. <https://doi.org/10.1016/j.genhosppsych.2021.09.002>
9. Lefevre, H., Stheneur, C., Cardin, C., Fourcade, L., Fourmaux, C., Tordjman, E., Touati, M., Voisard, F., Minassian, S., Chaste, P., Moro, M. R., & Lachal, J. (2021). The Bulle: Support and Prevention of Psychological Decompensation of Health Care Workers During the Trauma of the COVID-19 Epidemic. *J Pain Symptom Manage*, *61*(2), 416-422. <https://doi.org/10.1016/j.jpainsymman.2020.09.023>
10. Mellins, C. A., Mayer, L. E. S., Glasofer, D. R., Devlin, M. J., Albano, A. M., Nash, S. S., Engle, E., Cullen, C., Ng, W. Y. K., Allmann, A. E., Fitelson, E. M., Vieira, A., Remien, R. H., Malone, P., Wainberg, M. L., & Baptista-Neto, L. (2020). Supporting the well-being of health care providers during the COVID-19 pandemic: The CopeColumbia response. *Gen Hosp Psychiatry*, *67*, 62-69. <https://doi.org/10.1016/j.genhosppsych.2020.08.013>
11. Morina, N., Weilenmann, S., Dawson, K. S., Ernst, J., Zanitti, Z., von Känel, R., Schick, M., Spiller, T. R., & Bryant, R. A. (2021). RECHARGE - A Brief Psychological Intervention to Build Resilience in Health Care Workers During the COVID-19 Pandemic: Study Protocol for a Randomized Controlled Trial *Preprint* <https://doi.org/10.21203/rs.3.rs-212942/v1>
12. Sagaltici, E., Saydam, R. B., Cetinkaya, M., Şahin Ş, K., Küçük, S. H., & Müslümanoğlu, A. Y. (2022). Burnout and psychological symptoms in healthcare workers during the COVID-19 pandemic: Comparisons of different medical professions in a regional hospital in Turkey. *Work*, *72*(3), 1077-1085. <https://doi.org/10.3233/wor-210517>
13. Serrano-Ripoll, M. J., Ricci-Cabello, I., Jiménez, R., Zamanillo-Campos, R., Yañez-Juan, A. M., Bennasar-Veny, M., Sitges, C., Gervilla, E., Leiva, A., García-Campayo, J., García-Buades, M. E., García-Toro, M., Pastor-Moreno, G., Ruiz-Perez, I., Alonso-Coello, P., Llobera, J., & Fiol-deRoque, M. A. (2021). Effect of a mobile-based intervention on mental health in frontline healthcare workers against COVID-19: Protocol for a randomized controlled trial. *J Adv Nurs*, *77*(6), 2898-2907. <https://doi.org/10.1111/jan.14813>
14. Solomonov, N., Kanellopoulos, D., Grosenick, L., Wilkins, V., Goldman, R., Ritholtz, S., Falk, A., & Gunning, F. M. (2022). CopeNYP: A brief remote psychological intervention reduces health care workers’ depression and anxiety symptoms during COVID‐19 pandemic. *World Psychiatry*, *21*(1), 155-156. <https://doi.org/10.1002/wps.20946>
15. Sulaiman, A. H., Ahmad Sabki, Z., Jaafa, M. J., Francis, B., Razali, K. A., Juares Rizal, A., Mokhtar, N. H., Juhari, J. A., Zainal, S., & Ng, C. G. (2020). Development of a Remote Psychological First Aid Protocol for Healthcare Workers Following the COVID-19 Pandemic in a University Teaching Hospital, Malaysia. *Healthcare (Basel)*, *8*(3). <https://doi.org/10.3390/healthcare8030228> (Sulaiman et al., 2020)
16. Trottier, K., Monson, C. M., Kaysen, D., Wagner, A. C., Liebman, R. E., & Abbey, S. E. (2022). Initial findings on RESTORE for healthcare workers: an internet-delivered intervention for COVID-19-related mental health symptoms. *Transl Psychiatry*, *12*(1), 222. <https://doi.org/10.1038/s41398-022-01965-3>
17. Trottier, K., Monson, C. M., Kaysen, D., Wagner, A. C., Pun, C., & Abbey, S. E. (2021). Development of RESTORE: an online intervention to improve mental health symptoms associated with COVID-19-related traumatic and extreme stressors. *Eur J Psychotraumatol*, *12*(1), 1984049. <https://doi.org/doi:10.1080/20008198.2021.1984049>
18. Wang, L., Norman, I., Xiao, T., Li, Y., Li, X., & Leamy, M. (2022). Evaluating a Psychological First Aid Training Intervention (Preparing Me) to Support the Mental Health and Wellbeing of Chinese Healthcare Workers During Healthcare Emergencies: Protocol for a Randomized Controlled Feasibility Trial. *Front Psychiatry*, *12*, 809679. <https://doi.org/10.3389/fpsyt.2021.809679>
19. Weiner, L., Berna, F., Nourry, N., Severac, F., Vidailhet, P., & Mengin, A. C. (2020). Efficacy of an online cognitive behavioral therapy program developed for healthcare workers during the COVID-19 pandemic: the REduction of STress (REST) study protocol for a randomized controlled trial. *Trials*, *21*(1), 870. <https://doi.org/10.1186/s13063-020-04772-7>

# Research update

The research update of the systematic literature search II in MEDLINE via PubMed and PsychINFO and CINAHL via EBSCO took place on 26^th^ July 2024. Therefore, the publication date of the search filter was adapted according to the period, between May 2023 and July 2024.

Table 12. Search filters (own representation based on Nordhausen and Hirt (2022))

| **Search filter** |  |
| --- | --- |
| **Publication Date** | May 2023 – July 2024 |
| **Language** | English, German |
| **Spezies** | Human |

In total, 15 articles could be included via PubMed and 6 via PsychINFO. After removing 2 duplicates, 21 were screened within the full-text-screening, based on a not retrieved article (n = 1). The screening resulted in nine articles for data extraction and -analysis from the two databases. The limited systematic literature search in CINAHL resulted in 13 articles, whereas ten duplicates were removed, based on already identified articles from the other two databases. After the Title-/Abstract screening, none of the articles were eligible according to the prior defined inclusion criteria.

During the process of identifying articles via alternative methods, the seven articles resulting from the research update of the systematic literature search were screened for duplicates with the included articles from the current systematic literature search. Two articles were excluded, which resulted in five additional articles for analysis. With the backward citation tracking of included studies, another study could be identified coincide with the inclusion criteria.

Finally, with the research update, a total of 28 articles could be included based on the eligibility criteria for data analysis. The flow-chart including the research update can be seen in Figure 2.

Figure 2: PRISMA-Flow Chart including the research update according to Page et al., 2020

**Bibliographic informations of identified articles from the research update**

1. **Aragonès**, E., Rodoreda, S., Guitart, M., Garcia, E., Berenguera, A., Martin, F., Rambla, C., Aragonès, G., Calvo, A., Mas, A., & Basora, J. (2023). Study protocol: assessment of the usefulness and practicability of a psychoeducational intervention to prevent the negative psychological impact of the COVID-19 pandemic on primary care health workers. BMC Prim Care, 24(1), 231. <https://doi.org/10.1186/s12875-023-02187-2>
2. **Iyadurai** L.; Highfield J.; Kanstrup M.; Markham, A. R. V. G. B. J. T. K. J. G. G. S. C. B. M. (2023). Reducing instrusive memories after trauma via an imagery-competing task intervention in COVID-19 intensive care staff: a randomised controlled trial *Transl Psychiatry*, *13*(290), 1-15. <https://doi.org/10:1038/s41398-023-02578-0>
3. **Kanstrup** M, Singh L, Goransson KE, Gamble B, Taylor RS, Iyadurai L, et al. A simple cognitive task intervention to prevent intrusive memories after trauma in patients in the Emergency Department: A randomized controlled trial terminated due to COVID-19. BMC Res Notes. 2021;14(1):176.
4. **Kirykowicz**, K., Jaworski, B., Owen, J., Kirschbaum, C., Seedat, S., & van den Heuvel, L. L. (2023). Feasibility, acceptability and preliminary efficacy of a mental health self-management app in clinicians working during the COVID-19 pandemic: A pilot randomised controlled trial. Psychiatry Research, 329, 1-11. <https://doi.org/10.1016/j.psychres.2023.11549>
5. **Mediavilla**, R., Felez-Nobrega, M., McGreevy, K. R., Monistrol-Mula, A., Bravo-Ortiz, M. F., Bayón, C., Giné-Vázquez, I., Villaescusa, R., Muñoz-Sanjosé, A., Aguilar-Ortiz, S., Figueiredo, N., Nicaise, P., Park, A. L., Petri-Romão, P., Purgato, M., Witteveen, A. B., Underhill, J., Barbui, C., Bryant, R., . . . Ayuso-Mateos, J. L. (2023). Effectiveness of a mental health stepped-care programme for healthcare workers with psychological distress in crisis settings: a multicentre randomised controlled trial. *BMJ Ment Health*, *26*(1). <https://doi.org/10.1136/bmjment-2023-300697>
6. **Meredith**, L. S., Ahluwalia, S., Chen, P. G., Dong, L., Farmer, C. M., Bouskill, K. E., Dalton, S., Qureshi, N., Blagg, T., Timmins, G., Schulson, L. B., Huilgol, S. S., Han, B., Williamson, S., Watson, P., Schnurr, P. P., Martineau, M., Davis, K., Cassells, A., & Tobin, J. N. (2024). Testing an Intervention to Improve Health Care Worker Well-Being During the COVID-19 Pandemic: A Cluster Randomized Clinical Trial. *JAMA Network Open*, *7*(4), e244192-e244192. <https://doi.org/10.1001/jamanetworkopen.2024.4192>
7. **Morina**, N., Weilenmann, S., Dawson, K. S., Möckli, U., Ernst, J., Zanitti, Z., von Känel, R., Schick, M., Spiller, T. R., & Bryant, R. A. (2023). Efficacy of a Brief Psychological Intervention to Reduce Distress in Healthcare Workers During the COVID-19 Pandemic: A Randomized Controlled Trial. *Psychological Trauma: Theory, Research, Practice & Policy*, *15*, S371-S383. <https://doi.org/10.1037/tra0001524>
8. **Pratt**, E. H., Hall, L., Jennings, C., Olsen, M. K., Jan, A., Parish, A., Porter, L. S., & Cox, C. E. (2023). Mobile Mindfulness for Psychological Distress and Burnout among Frontline COVID-19 Nurses: A Pilot Randomized Trial. *Ann Am Thorac Soc*, *20*(10), 1475-1482. <https://doi.org/10.1513/AnnalsATS.202301-025OC>
9. **Singh**, L., Kanstrup, M., Gamble, B., Geranmayeh, A., Goransson, K. E., Rudman, A., Dahl, O., Lindstrom, V., Horberg, A., Holmes, E. A., & Moulds, M. L. (2022). A first remotely-delivered guided brief intervention to reduce intrusive memories of psychological trauma for healthcare staff working during the ongoing COVID-19 pandemic: Study protocol for a randomised controlled trial. *Contemp Clin Trials Commun*, *26*, 100884. <https://doi.org/10.1016/j.conctc.2022.100884>

# References

Aragonès, E., Rodoreda, S., Guitart, M., Garcia, E., Berenguera, A., Martin, F., Rambla, C., Aragonès, G., Calvo, A., Mas, A., & Basora, J. (2023). Study protocol: assessment of the usefulness and practicability of a psychoeducational intervention to prevent the negative psychological impact of the COVID-19 pandemic on primary care health workers. *BMC Prim Care*, *24*(1), 231. <https://doi.org/10.1186/s12875-023-02187-2>

Cooper, C., Booth, A., Britten, N., & Garside, R. (2017). A comparison of results of empirical studies of supplementary search techniques and recommendations in review methodology handbooks: a methodological review. *Syst Rev*, *6*(1), 234. <https://doi.org/10.1186/s13643-017-0625-1>

Damschroder, L. J., Aron, D. C., Keith, R. E., Kirsh, S. R., Alexander, J. A., & Lowery, J. C. (2009). Fostering implementation of health services research findings into practice: a consolidated framework for advancing implementation science. *Implement Sci*, *4*, 50. <https://doi.org/10.1186/1748-5908-4-50>

Damschroder, L. J., Reardon, C. M., Widerquist Opra, M. A., & Lowery, J. (2022). The updated Consolidated Framework for Implementation Research based on user feedback. *Implementation Science*, *17*(1). <https://doi.org/10.1186/s13012-022-01245-0>

Evanoff, B. A., Strickland, J. R., Dale, A. M., Hayibor, L., Page, E., Duncan, J. G., Kannampallil, T., & Gray, D. L. (2020). Work-Related and Personal Factors Associated With Mental Well-Being During the COVID-19 Response: Survey of Health Care and Other Workers. *Journal of Medical Internet Research*, *22*(8), e21366. <https://doi.org/10.2196/21366>

Kunkler, C. (2023). *Implementation of a clinical decision support system using virtual reality for psychiatric technician: A feasibility and acceptability project* ProQuest Information & Learning]. APA PsycInfo. <https://uni-wh.idm.oclc.org/login?url=https://search.ebscohost.com/login.aspx?direct=true&db=psyh&AN=2023-33087-170&site=ehost-live&scope=site>

McGowan, J., Sampson, M., Salzwedel, D. M., Cogo, E., Foerster, V., & Lefebvre, C. (2016). PRESS Peer Review of Elektronic Search Strategies: 2015 Guideline Statement *Journal of Clinical Epidemiology*, *75*. <https://doi.org/10.1016/j.jclinepi.2016.01.021>

Nordhausen, T., & Hirt, J. (2022). RefHunter im neuen Webformat: Eine Plattform zur systematischen Literaturrecherche *GMS Medizin 22*(2). <https://doi.org/10.3205/mbi000549>

Ouzzani, M., Hammady, H., Fedorowicz, Z., & Elmagarmid, A. (2016). Rayyan—a web and mobile app for systematic reviews. *Systematic Reviews*, *5*(1). <https://doi.org/10.1186/s13643-016-0384-4>

Peters, M. D. J., Marnie, C., Tricco, A. C., Pollock, D., Munn, Z., Alexander, L., McInerney, P., Godfrey, C. M., & Khalil, H. (2020). Updated methodological guidance for the conduct of scoping reviews. *JBI Evid Synth*, *18*(10), 2119-2126. <https://doi.org/10.11124/JBIES-20-00167>

Saragih, I. D., Tonapa, S. I., Saragih, I. S., Advani, S., Batubara, S. O., Suarilah, I., & Lin, C. J. (2021). Global prevalence of mental health problems among healthcare workers during the Covid-19 pandemic: A systematic review and meta-analysis. *Int J Nurs Stud*, *121*, 104002. <https://doi.org/10.1016/j.ijnurstu.2021.104002>

Stetler, C. B., Legro, M. W., Wallace, C. M., Bowman, C., Guihan, M., Hagedorn, H., Kimmel, B., Sharp, N. D., & Smith, J. L. (2006). The Role of Formative Evaluation in Implementation Research and the QUERI Experience. *Journal of General Internal Medicine*, *21*(S2), S1-S8. <https://doi.org/10.1111/j.1525-1497.2006.00355.x>

Stuijfzand, S., Deforges, C., Sandoz, V., Sajin, C.-T., Jaques, C., Elmers, J., & Horsch, A. (2020). Psychological impact of an epidemic/pandemic on the mental health of healthcare professionals: a rapid review. *BMC Public Health*, *20*(1). <https://doi.org/10.1186/s12889-020-09322-z>

Taylor-Desir, M. (2022). *What is Posttraumatic Stress Disorder (PTSD)* Amercian Psychiatric Association <https://www.psychiatry.org/patients-families/ptsd/what-is-ptsd>

Wensing, M., Sales, A., Armstrong, R., & Wilson, P. (2020). Implementation science in times of Covid-19. *Implement Sci*, *15*(1), 42. <https://doi.org/10.1186/s13012-020-01006-x>

WHO. (2019). *International Statistical Classification of Diseases and Related Health Problems 10th Revision (ICD-10)-WHO Version for 2019-covid-expanded*

*Chapter V Mental and behavioral disorders (F00-F99)* World Health Organization <https://icd.who.int/browse10/2019/en#/F43.2>

Zhang, L., Li, L., Zheng, W., Zhang, Y., Gao, X., Tan, L., Wang, X., Chen, Q., Xu, J., Tang, J., Luo, X., Chen, X., Zhang, X., He, L., Liu, J., Cheng, P., Xu, L., Tian, Y., Wen, C., & Li, W. (2023). Psychosocial crisis intervention for coronavirus disease 2019 patients and healthcare workers. *Zhong Nan Da Xue Xue Bao Yi Xue Ban*, *48*(1), 92-105. <https://doi.org/10.11817/j.issn.1672-7347.2023.210803> (新型冠状病毒肺炎患者和医务人员的心理社会危机干预（英文）.)
