# Supplementary material for: Factors influencing the implementation of interventions for symptoms of posttraumatic stress disorder among hospital-based nurses and physicians during the COVID-19 pandemic: a scoping review
Source: BMC Health Serv Res. 2025 Jul 2;25:885. doi: 10.1186/s12913-025-13005-z (PMC12225526; doi:10.1186/s12913-025-13005-z)
Supplement: Supplementary file 4 — Additional File 4. Influencing factors. [file 12913_2025_13005_MOESM4_ESM.docx]

## ***Additional File 4: Influencing factors in the implementation of PTSD-related interventions***

| **Outer Setting Domain** | | | | |
| --- | --- | --- | --- | --- |
| **Construct Name** | **Barrier** | **Facilitator** | **Neutral factor** | **Studies contributing to findings** |
| A. Critical Incidents |  |  | - The COVID-19 pandemic (crisis) causes increased hospitalization rate based on cases of COVID-19 and therefore an influencing factor for several reasons, e.g., less of time due to higher workload stressful conditions **^1,2,3,4,5,6,7,8,9, 10^** | Kanellopoulos et al. (2021)^1^; Morina et al. (2021)^2^; Trottier et al. (2022)^3^; Mellins et al. (2020)^4^; Weiner et al. (2020)^5^, Albott et al. (2020)^6^; Bureau et al. (2021)^7^; Jovarauskaite et al. (2021)^8^; Morina et al. (2023)**^9^**; Singh et al. (2022) **^10^** |
| B. Local Attitudes |  |  | - External stigma regarding HCWs seeking help for mental wellbeing may have an impact on the requirements for use those interventions. **^1, 2, 3, 4, 5,^** **^6^** | Trottier et al. (2022)**^1^**; Wang et al. (2022)**^2^**; Mellins et al. (2020)**^3^**; Trottier et al. (2021)**^4^**; Ahmed Pihlgren et al. (2024)**^5^**; Iyadurai et al. (2023)**^6^** |
| C. Local Conditions |  |  | - Financial and/ or logistical (e.g., functional technical infrastructure) conditions **^1, 2^** - Not specified **^3^** - During the pandemic (e.g. physical distancing rules) **^4^** | Trottier et al. (2021)**^1^**; Trottier et al. (2022)**^2^**; Mediavilla et al. (2023)**^3^**; Morina et al. (2023) **^4^** |
| D. Partnership & Connections |  | - Disseminate the intervention with an external professional network **^3^** | - A partnership with members from faculties at a university **^1, 2, 3,4^** - Collaboration with Society **^5^** | Albott et al. (2020)**^1^**, Kanellopoulos et al. (2021)**^2^**; Blake et al. (2021)**^3^**; Mellins et al. (2020)**^4^**; Iyadurai et al. (2023)**^5^** |
| E. Policies & Laws |  |  | - *Regulations* (e.g., Establishment of a health hygiene protocol for face-to-face interventions) **^1^** - Guideline development based on *guidelines* from Ministry of Health and WHO-standard protocol **^2^** | Lefevre et al. (2021)**^1^**; Sulaiman et al. (2020)**^2^** |
| F. Financing |  |  | - Funding through the health insurance (long-term) **^1^** - Costs must be calculated for the purpose of maintenance and long-term use **^2^** | Hannig et al. (2021)**^1^**; Blake et al. (2020)**^2^** |
| G. External Pressure |  |  | - In terms of urgency based on the needs of HCWs**^1^** - From a collaborative university as part of a study **^2^** | Hannig et al. (2021)**^1^**; Dong et al. (2022)**^2^** |
| **Inner Setting Domain** | | | | |
| **Construct Name** | **Barrier** | **Facilitator** | **Neutral factor** | **Studies contributing to findings** |
| A. Structural Characteristics |  |  | - Establishment of a medical rescue team as part of a designated emergency response hospital **^1^** - Strong administrative support infrastructure; national medical rescue team **^2^** | Wang et al. (2022)**^1^**; Kanellopoulos et al. (2021)**^2^** |
| A. Structural Characteristics  1. Physical Infrastructure |  |  | - Able to relocate space and/ or to generate some space **^1, 2^** | Sulaiman et al. (2020)**^1^**; Lefevre et al. (2021)**^2^** |
| A. Structural Characteristics  2. Information Technology Infrastructure |  |  | - Data storage **^1^** - Technological system for tele-communication (based on the type of innovation) **^2^** - Difficulty to find access to desktop computers **^3^** | Sulaiman et al. (2020)**^1^**; Kanellopoulos et al. (2021)**^2^**; Singh et al. (2022) **^3^** |
| A. Structural Characteristics  3. Work Infrastructure |  |  | - Regular critical incident response training **^1^** - A centralized response emergency system for employees **^1^** | Wang et al. (2022)**^1^** |
| B. Relational Connections |  |  | - Using formal/ informal relationship between fellows for dissemination/ recommendation **^1^** - Team-working **^1, 3^** - Intraprofessional collaboration **^2, 4^** - Network (outer setting) **^4, 5,^** | Kanellopoulos et al. (2021)**^1^**; Sulaiman et al. (2020)**^2^**; Lefevre et al. (2021)**^3^**; Albott et al. (2020)**^4^**; Wang et al. (2022)**^5^** |
| C. Communications |  | - Sharing the innovation within their clinical teams **^4^** | - Formal and informal communication in the inner setting (introduction/ announcement into/ about the innovation) **^1, 2, 3^** - Mentioning the intervention to colleagues **^5^** | Solomonov et al. (2022)**^1^**; Hannig et al. (2021)**^2^**; Kanellopoulos et al. (2021)**^3^**; Blake et al. (2020)**^4^**; Ahmed Pihlgren et al. (2024) **^5^** |
| D. Culture |  |  | - Active mobilization of teams **^1^** - Diversity (cultural/ demographically) **^2^** - Inner organization culture (not focus on emotions/ asking for help/ showing compassion to each other) **^3^** | Lefevre et al. (2021)**^1^**; Albott et al. (2020)**^2^**; Mellins et al. (2020)**^3^** |
| D. Culture  1. Human-Equality-Centeredness |  |  | - The providers must act with respect, dignity and in sense of the human-rights with fellows **^1^** | Sulaiman et al. (2020)**^1^** |
| D. Culture  2. Recipient-Centeredness |  |  | - Use of the innovation also for patients **^1^** | Bureau et al. (2021)**^1^** |
| D. Culture  3. Deliverer-Centeredness |  | - Providing support from the institution, demonstrate estimation **^5^** - The opportunity given as a workplace to claim the resource **^3^** | - Participation of employees **^1, 3^** - The organization listen to and understand the needs of employees **^2^** - Commitment of care about the mental health of employees **^4^** - Commitment to providing training means that decision-makers need to consider how shift the organizational culture toward an investment in HCWs well-being **^7^** | Albott et al. (2020)**^1^**; Dong et al. (2022)**^2^**; Hannig et al. (2021)**^3^**; Sulaiman et al. (2020)**^4^**; Blake et al. (2021)**^5^**; Lefevre et al. (2021)**^6^**; Meredith et al. (2024) **^7^** |
| D. Culture  2. Learning Centeredness |  |  | - Learning from another **^1^** - Opportunity for experimental teaching and training **^2^** - Improvement of capacity/ wellbeing of employees **^2^** - Advancement of further support-programs **^3^** | Albott et al. (2020)**^1^**; Wang et al. (2022)**^2^**; Hannig et al. (2021)**^3^** |
| E. Tension for Change |  | - The awareness of needs for support in terms of coping with the impact of the COVID-19 pandemic **^2^** | - Acknowledgement of the psychological impact from the COVID-19 pandemic on the psychological well-being of employees **^1^** | Sulaiman et al. (2020)**^1^**; Hannig et al. (2021)**^2^** |
| F. Compatibility |  | - Compatibility with the schedules (busy/ high flexible) **^8^** - The intervention is flexible for using it in different settings, which facilitate the implementation in terms of compatibility with the delivering context **^8, 10^** - The intervention is easily adaptable to everyday life **^11^** | - Working conditions (e.g., stressful/ “normal”) **^1, 2, 4, 7, 9, 12, 14^** - Long working day **^9, 12^** - Less time **^2, 8, 12, 16^** - Flexibility of the intervention for using when time is available and in everyday life **^2, 3, 5, 13, 15^** | Hannig et al. (2021)**^1^**; Morina et al. (2021)**^2^**; Dong et al. (2022)**^3^**; Albott et al. (2020)**^4^**; Sagaltici et al. (2022)**^5^**; Weiner et al. (2020)**^6^**; Lefevre et al. (2021)**^7^**; Bureau et al. (2021)**^8^**; Jovarauskaite et al. (2021)**^9^**; Blake et al. (2020)**^10^**; Ahmed Pihlgren et al. (2024) **^11^**; Iyadurai et al. (2023) **^12^**; Mediavilla et al. (2023) **^13^**; Morina et al. (2023) **^14^**; Pratt et al. (2023) **^15^**; Singh et al. (2022) **^16^** |
| H. Incentive Systems |  |  | - Weekly meetings for performance feedback **^1^** | Albott et al. (2020)**^1^** |
| J. Available Resources |  |  | - Human resource **^1, 2, 3, 4^** - Specialized and rapid access to resources **^2^** - Time **^2^** | Mellins et al. (2020)**^1^**; Albott et al. (2020)**^2^**; Lefevre et al. (2021)**^3^**; Morina et al. (2023) **^4^** |
| J. Available Resources  1. Funding |  |  | - Internal funding **^1^** | Hannig et al. (2021)**^1^** |
| J. Available Resources  2. Space |  |  | - Available space for the innovation (face-to-face-innovation) within in the inner setting (e.g., hospital) **^1, 2^** | Albott et al. (2020)**^1^**; Lefevre et al. (2021)**^2^** |
| J. Available Resources  3. Materials & Equipment |  |  | - Access to material and equipment for delivering the innovation **^1, 2^** | Albott et al. (2020)**^1^**; Bureau et al. (2021)**^2^** |
| K. Access to Knowledge & Information |  |  | - Coaches receive training in delivering the innovation **^1^** - Guidance for promoting engagement and increase adherence **^2^** - Concurrent training for providers**^3^** - Improvement of knowledge and skills from the trainers **^4^** - Access to guidance/ training **^5, 6^** | Morina et al. (2021)**^1^**; Trottier et al. (2021)**^2^**; Sulaiman et al. (2020)**^3^**; Wang et al. (2022)**^4^**; Kanellopoulos et al. (2021)**^5^**; Hannig et al. (2021)**^6^** |
| **Individuals Domain** | | | | |
| ***Roles Subdomain – Leaders*** | | | | |
| **Construct Name** | **Barrier** | **Facilitator** | **Neutral factor** | **Studies contributing to findings** |
| A. High-level Leaders | - High-level leaders may not need the intervention **^1^** |  | - Department (Heads) **^1^** - Site leader (e.g., Chief Medical Officer, Chief Nursing Officers) **^2^** - Hospital leadership **^3^** | Albott et al. (2020)**^1^**; Dong et al. (2022)**^2^**; Kanellopoulos et al. (2021)**^3^** |
| B. Mid-level Leaders |  | - Team leaders get the scope to fulfil the role, e.g., signpost some sections of the innovation **^1^** |  | Blake et al. (2020)**^1^** |
| C. Opinion Leaders |  | - Health-care workers (functioned as peers) **^4^** | - Health-care workers (functioned as peers) **^1,3, 5, 6^** - Innovation deliverer **^2^** | Morina et al. (2021)**^1^**; Trottier et al. (2022)**^2^**; Hannig et al. (2021) **^3^**; Ahmed Pihlgren et al. (2024) **^4^**, Meredith et al. (2024) **^5^**; Morina et al. (2023) **^6^** |
| D. Implementation Facilitators |  |  | - Mental health consultant, with subject expertise **^1^** - Skilled clinicians (with advanced experience in evidence-based psychological interventions) **^2^** - Site “Champion” **^3^** - Provider of the innovation **^4^** | Albott et al. (2020)**^1^**; Kanellopoulos et al. (2021)**^2^**; Dong et al. (2022)**^3^**; Mellins et al. (2020)**^4^** |
| E. Implementation Leads |  |  | - Peers **^1^** - “Champions” led the implementation of the innovation, locally **^2, 3^** - Mental health providers (psychiatry, clinical psychology and mental health nursing trainees) **^3^** | Hannig et al. (2021)**^1^**; Dong et al. (2022)**^2^**; Mediavilla et al. (2023) **^3^** |
| F. Implementation Team Members |  |  | - Included also the innovation delivers (clinical staff), as well as the innovation recipients (support staff) **^1^** - Implementation facilitators, as well as the innovation delivers **^2^** | Kanellopoulos et al. (2021)**^1^**; Mellins et al. (2020)**^2^** |
| H. Innovation Deliverers |  | - Individuals get the scope to fulfil this role (signpost some particular sections of the innovation) **^1, 9^** - Innovation recipients need deliverers to introduce into the intervention and to gain understanding of the intervention **^10^** | - Volunteering clinicians **^1^** - Guides by non-psychotherapists **^2^** - Trainer **^3, 11^** - Skilled clinicians (with advanced training in evidence-based psychological interventions) **^1, 7^** - Peers (e.g., nurses, physicians, psychiatrists) **^4, 5, 6^** - Health-care workers themselves **^8^** | Kanellopoulos et al. (2021)**^1^**; Trottier et al. (2022)**^2^**; Wang et al. (2022)**^3^**; Morina et al. (2021)**^4^**; Lefevre et al. (2021)**^5^**; Hannig et al. (2021)**^6^**; Sulaiman et al. (2020)**^7^**; Bureau et al. (2021)**^8^**, Blake et al. (2020)**^9^**; Ahmed Pihlgren et al. (2024) **^10^**; Mediavilla et al. (2023) **^11^** |
| I. Innovation Recipients | - Opportunity and the capability could be barriers regarding the delivery of the innovation **^1, 4, 5, 6^** - The intervention at one time, when they needed support was not a plausible option **^5^** | - Professionals act like facilitators and have time to fulfil the role **^1, 4, 5^** - Health care workers, when they are aware of what they needed when and how **^5^** | - Health-care workers (e.g., nurses, physicians) **^1, 2, 3, 7, 8, 9^** | Bureau et al. (2021)**^1^**; Kanellopoulos et al. (2021)**^2^**; Lefevre et al. (2021)**^3^**; Blake et al. (2020)**^4^**; Ahmed Pihlgren (2024) **^5^**; Kirykowisk et al. (2023) **^6^**; Meredith et al. (2024) **^7^**; Pratt et al. (2023) **^8^**; Singh et al. (2022) **^9^** |
| ***Characteristics Subdomain*** | | | | |
| **Construct Name** | **Barrier** | **Facilitator** | **Neutral factor** | **Studies contributing to findings** |
| A. Need | - High-level leaders may not feel the innovation is necessary **^4^** | - Innovation recipients were aware of their mental status and when they needed the intervention **^5^** | - Innovation-Delivers experienced similar stressors by providing the innovation to fellows as innovation-recipients **^1^** - Innovation-recipients have deficits in their well-being (e.g., stress from work, dysregulation of emotions) **^1, 2^** | Kanellopoulos et al. (2021)**^1^**; Bureau et al. (2021)**^2^**; Lefevre et al. (2021)**^3^**; Albott et al. (2020)**^4^**; Ahmed Pihlgren (2024) **^5^** |
| B. Capability | - Innovation-recipients mention concerns of not having the technical skills for using the innovation (digital innovation) **^8, 11^** - Innovation recipients were not receptive to use the intervention in times when they really needed it **^9^** | - Innovation delivers (e.g., clinicians) face challenges in delivery, but innovation is aligned with values, so the outcome is a meaningful experience **^1^** - Innovation-recipients evolve a high confidence in their capabilities (e.g., using the innovation in daily life and circumstances) **^7^** | - Skills of innovation delivers (e.g., clinicians, mental health providers) **^1, 3^** - Implementation facilitators **^1,^** **^2, 4^** - Implementation Leads (e.g., peers) **^4, 5^** - Opinion Leaders (e.g., peers) **^5, 6, 11^** - Innovation-Recipients (e.g., technical capabilities for online-delivered innovations) **^7, 11^** | Kanellopoulos et al. (2021)**^1^**; Mellins et al. (2020)**^2^**; Wang et al. (2022)**^3^**; Dong et al. (2022)**^4^**; Hannig et al. (2021)**^5^**; Albott et al. (2020)**^6^**; Bureau et al. (2021)**^7^**; Blake et al. (2020)**^8^**; Ahmed Pihlgren et al. (2024) **^9^**; Kirykowisk et al. (2023) **^10^**; Morina et al. (2023) **^11^** |
| C. Opportunity | - Innovation-recipients mention that they have not enough time to conduct the intervention completely (because of the workload and resulted time), so they have not opportunity in sense of scope to fulfil the role **^1, 9, 11^** | - Innovation-recipients have the sufficient time allocation to fulfil the role as innovation-recipients **^8, 10^** - Mid-leaders and concurrent innovation-deliverer have the scope to fulfil this role and could signpost some section of the innovation to their team members **^7^** - Innovation-delivers (e.g., clinicians) are familiar with the characteristics of the inner setting **^3^** | - Sufficient time allocation (for innovation-recipients) **^12, 13, 14^** - Scope **^1, 2^** - High-level leaders support the intervention **^3^** - Availability of implementation facilitator **^6^** | Bureau et al. (2021)**^1^**, Hannig et al. (2021)**^2^**; Kanellopoulos et al. (2021)**^3^**; Albott et al. (2020)**^6^**; Blake et al. (2021)**^7^**; Lefevre et al. (2021) **^8^**; Trottier et al. (2022) **^9^**; Ahmed Pihlgren et al. (2024) **^10^**; Kirykowisk et al. (2023) **^11^**; Meredith et al. (2024) **^12^**; Pratt et al. (2023) **^13^**; Singh et al. (2022) **^14^** |
| D. Motivation | - Innovation-recipients found, that the intervention was not a plausible option at the time where they needed support **^7^** - Innovation-recipients could not generated impacted engagement with the intervention **^8^** | - Innovation delivers have a positive attitude for using the innovation (digital) and could generate enthusiasm to motivate other fellows (recommendation) **^1, 2, 6, 7^** - Opinion leaders, like the health care workers themselves functioned as peers were highly attitude to use the intervention **^7^** - Innovation recipients have to trust in the persons who delivers the intervention **^7^** | - Department (Heads) **^1,^** - Innovation-Delivers (enthusiasm) - Opinion Leaders, e.g., peers **^2, 3, 4, 5^** - Innovation-Recipients (e.g., are enthusiastic) **^5^** | Albott et al. (2020)**^1^**; Bureau et al. (2021)**^2^**; Lefevre et al. (2021)**^3^**; Kanellopoulos et al. (2021)**^4^**; Dumarkaite et al. (2023)**^5^**; Blake et al. (2021)**^6^**; Ahmed Pihlgren et al. (2024) **^7^**; Kirykowisk et al. (2023) **^8^** |

Conceptual paper, Empirical paper, Study protocol
